# An Expanded Inventory of Conserved Meiotic Genes Provides Evidence for Sex in *Trichomonas vaginalis*

**DOI:** 10.1371/journal.pone.0002879

**Published:** 2008-08-06

**Authors:** Shehre-Banoo Malik, Arthur W. Pightling, Lauren M. Stefaniak, Andrew M. Schurko, John M. Logsdon

**Affiliations:** Department of Biology, Roy J. Carver Center for Comparative Genomics, University of Iowa, Iowa City, Iowa, United States of America; Indiana University, United States of America

## Abstract

Meiosis is a defining feature of eukaryotes but its phylogenetic distribution has not been broadly determined, especially among eukaryotic microorganisms (*i.e.* protists)—which represent the majority of eukaryotic ‘supergroups’. We surveyed genomes of animals, fungi, plants and protists for meiotic genes, focusing on the evolutionarily divergent parasitic protist *Trichomonas vaginalis*. We identified homologs of 29 components of the meiotic recombination machinery, as well as the synaptonemal and meiotic sister chromatid cohesion complexes. *T. vaginalis* has orthologs of 27 of 29 meiotic genes, including eight of nine genes that encode meiosis-specific proteins in model organisms. Although meiosis has not been observed in *T. vaginalis*, our findings suggest it is either currently sexual or a recent asexual, consistent with observed, albeit unusual, sexual cycles in their distant parabasalid relatives, the hypermastigotes. *T. vaginalis* may use meiotic gene homologs to mediate homologous recombination and genetic exchange. Overall, this expanded inventory of meiotic genes forms a useful “meiosis detection toolkit”. Our analyses indicate that these meiotic genes arose, or were already present, early in eukaryotic evolution; thus, the eukaryotic cenancestor contained most or all components of this set and was likely capable of performing meiotic recombination using near-universal meiotic machinery.

## Introduction

Meiosis is a necessary part of sexual reproduction and a hallmark of eukaryotes that distinguishes them from prokaryotes, yet we are only beginning to understand its origin and evolution. Recent work has revealed that many meiotic genes are conserved not only among animals, fungi and plants (AFP) and some eukaryotic microorganisms (protists), but also in the putatively early-diverged protist *Giardia intestinalis* [1] which is not known to be sexual *per se* but was recently shown to have genetic recombination [2], [3] and to use orthologs of meiosis-specific genes in putatively parasexual recombination processes [4]. The breadth of eukaryotic diversity lies among the protists [5], [6], yet much remains to be elucidated about their meiotic machinery [1], [7], [8]. Thus, we have continued and expanded our search for conserved meiotic genes in public databases and particularly in the recently completed genome of *Trichomonas vaginalis* [9], a member of the Parabasalia.

Parabasalids are a highly diverged eukaryotic lineage in which the molecular mechanisms of meiosis are unexamined; they are related (albeit distantly) to diplomonads (*e.g. Giardia*) [10]–[17]. Morphological and molecular phylogenetic data, while controversial in details, divide parabasalids into two groups, the hypermastigotes (symbionts of roaches and termites, *e.g.* Trichonymphida) and the trichomonads (parasitic and free-living flagellates, *e.g.* Trichomonadidae) [18]–[23]. *T. vaginalis* is sexually transmitted between people's urogenital tracts, and acute infections are associated with increased risk of pelvic inflammatory disease, HIV-1 infection, infertility and problems with pregnancy [24], [25]. *T. vaginalis* is estimated to cause 174 million new infections annually worldwide and is the most common non-viral sexually transmitted human pathogen [25], [26]. Metronidazole is commonly used to treat *T. vaginalis* infections, but resistance to the drug is increasing [27]. It is not known whether genetic exchange occurs in populations of *T. vaginalis*; however, genetic exchange could mediate the proliferation of drug-resistant mutations or increased virulence in populations of the parasite.

While neither meiosis nor sex has been observed in *Trichomonas* or other trichomonads, various observations suggest the presence of sexual processes in Parabasalids. Using light microscopy, Cleveland described insect-hormone-induced divisions in hypermastigotes as one-step meiosis, and suggested that this was a more primitive form of meiosis than typical two-step meiosis in AFP [28]–[30]. For trichomonads, the finding of six genetically identical strains of *T. vaginalis* with clonal population structure was taken as evidence against meiotic recombination [31]. However, recent phylogenetic analyses of 731 polymorphic molecular markers show genetic variation among 20 strains of *T. vaginalis* that may be sufficient to indicate meiotic recombination [32]. Similar analyses reveal that closely-related *T. vaginalis* strains shared the phenotype of resistance to metronidazole, but this pattern had no correlation with geographical origin [33], suggesting the genetic spread of resistance by recombination and strong selection. The recent identification of *mariner*, *Maverick* and other DNA transposons in high copy number [34]–[36] in the highly repetitive >160 Mb genome sequence of *T. vaginalis* strain G3 [9], [37] could be the result of a predominantly asexual mode of reproduction or the recent loss of sexual reproduction [38]. In contrast, the presence of intact retroposons and reverse transcriptase homologs in the *T. vaginalis* strain G3 genome sequence [9] is consistent with an expectation that such elements, that are predominantly vertically transmitted, are only maintained in sexual lineages [39]. Although meiosis was not observed in extensive cytological studies of cell division in *T. vaginalis* [40], [41], it has been noted that its six chromosomes may be synapsed in 0.1% of cells, suggesting that meiosis may occur transiently in lab populations [42]. Quadrinucleated cells transiently observed in lab populations were noted but dismissed as not indicating a developmental stage in *T. vaginalis* [43]. Thus, with little direct evidence for meiosis in *T. vaginalis*, an inventory of meiotic genes will be an informative tool with which to assess its ability to undergo sexual processes.

Meiosis remains to be described at the molecular level in parabasalids and in most other protist lineages. Since parabasalids could represent one of the earliest diverging lineages on the tree of eukaryotes [11]–[14] and they may employ non-canonical meiosis, their meiotic processes could represent an ancestral state. Thus, an understanding of the molecular mechanisms underlying meiosis in these protists is important. Comparative studies of meiotic machinery (i) may indicate the presence of sexual reproduction in recent ancestors of organisms that are sexual or truly asexual (*vs.* just facultatively sexual), (ii) could indicate the absence of sex, (iii) will be useful for studies of the evolutionary advantages of sex, and (iv) can provide data that are valuable for ecological and epidemiological studies [44]. Surveys of meiotic genes have not been performed in most protists, making a comparative analysis of meiosis incomplete due to the limited available gene sequence data from diverse protists [45]. Thus, the universality of meiotic machinery in eukaryotes remains an open question.

**Figure 1:**
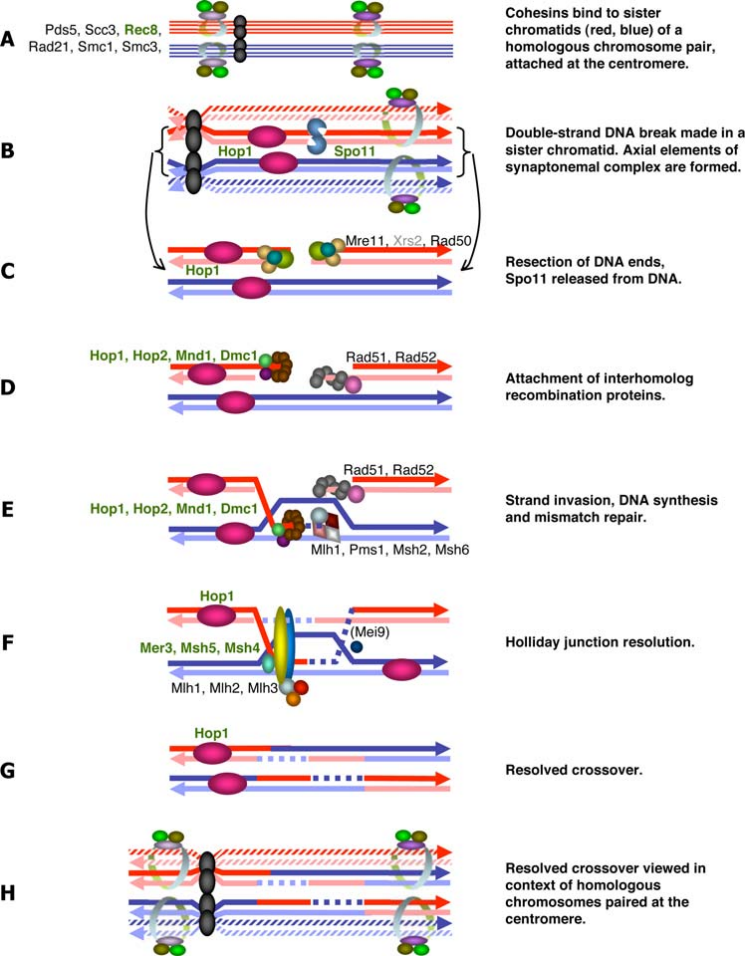
The double-strand break repair model of meiotic recombination, depicting interactions among proteins included in this study. The names of meiosis-specific proteins are highlighted in green. Exact stoichiometry is not implied. In meiosis I, cohesins bind to sister chromatids (A), after which double-strand DNA breaks are made by Spo11 (accessory proteins not shown) and the axial elements (Hop1) of the synaptonemal complex are formed (B). Double strand break repair is initiated (coupled with (B) in *S. cerevisiae*) and Hop1 forms lateral elements of the synaptonemal complex (C). Strand exchange proteins are attracted to the double-strand break (accessory proteins not shown) (D). The resulting heteroduplex (E) may be resolved by crossovers, which utilize meiosis-specific proteins (F), or by gene conversion, which does not (G, proteins not shown). This model is based primarily upon details from *S. cerevisiae*, but includes details from mammals for Msh4 and Msh5, and speculates on the role of *Drosophila* Mei-9 (Rad1) in (F) as reviewed by [54], [97]–[100]. Table 1 gives additional details and references.

We surveyed the genomes of *Trichomonas vaginalis* (strains G3 and NIH-C1) and other diverse eukaryotes for a previously-described set of 17 conserved meiotic genes [1] and 12 additional meiotic genes also conserved among eukaryotes (**Table 1** and **Figure 1**). Our search included 9 genes that are “meiosis-specific” since they are only known to function in meiosis in AFP and thus hypothesized to only be present in organisms with sexual ancestry (*Spo11*, *Hop1*, *Hop2*, *Mnd1*, *Dmc1*, *Msh4*, *Msh5*, *Mer3*, *Rec8*). We also surveyed 20 additional genes whose products are required for meiosis in AFP but also have general functions in DNA repair or mitosis (*Mre11*, *Rad50*, *Rad1*, *Rad52*, *Rad51*, *Msh2*, *Msh6*, *Mlh1–Mlh3*, *Pms1*, *Smc1–Smc6*, *Rad21*, *Scc3*, *Pds5*). We searched public databases to find homologs of meiotic proteins in diverse eukaryotes with complete (at least 7× coverage) genome sequence. Completed and near-complete genome sequences include AFP and representative apicomplexan, ciliate, chromist, amoebozoan, trypanosomatid and diplomonad protists, in addition to *T. vaginalis*. Meiotic gene sequences from *T. vaginalis* strain G3 found by this method were amplified by the polymerase chain reaction (PCR) from strain NIH-C1 and sequenced. Homology of the meiotic proteins was validated by phylogenetic analysis, by which we determined if the homologous genes were orthologs (related by speciation events) or paralogs (related by gene duplication). Our results indicate that homologs of a diverse set of meiotic genes are widespread among eukaryotes.

**Table 1:** Core meiotic genes and some key functions of their encoded proteins in meiosis.

Protein	Function
**Spo11**	Transesterase, creates DNA double-strand breaks (DSB) in homologous chromosomes [103]–[105].
Mre11	3′–5′ dsDNA exonuclease and ssDNA endonuclease, trims back broken DNA ends and hairpins [106]–[108].
Rad50	Dimer, holds broken DNA ends together while Mre11 trims. ATPase, has DNA-binding activity [105], [108].
Rad1 (Mei9)	Forms a heterodimer with Rad10 (Ercc1) [109]. 5′>3′ endonuclease, essential for nucleotide excision repair. Required for meiotic crossing over, normal meiotic chromosome disjunction, to repair mismatches in heteroduplex DNA and to resolve reciprocally exchanged recombination intermediates in *Drosophila* [110].
**Hop1**	Protein that binds DSBs and oligomerizes early during meiotic prophase, and forms axial and lateral elements of the synaptonemal complex [111].
**Hop2**	With **Mnd1**, ensures accurate and efficient homology searching, downstream of Rad51 and **Dmc1**, during pachytene stage of meiotic prophase [112].
**Mnd1**	With **Hop2**, functions after meiotic DSB formation, and required for stable heteroduplex DNA formation [113].
Rad52	Binds to the ssDNA ends of DSBs and initiates DSB repair by homologous recombination [114]. Stimulates Rad51-mediated strand invasion by interaction with Rad51 and RPA, and promotes single strand annealing (SSA) [115].
**Dmc1**	Meiosis-specific homolog of Rad51, has similar function but promotes interhomolog recombination [116]–[118].
Rad51	Forms helical filaments on single-stranded and double-stranded DNA and catalyzes homologous DNA pairing and strand exchange. (Intrahomologous recombination) [116], [118].
**Msh4**	Forms a heterodimer with **Msh5**, interacts with Mlh1/Mlh3 heterodimer. Directs Holliday junction resolution towards crossover with interference [119].
**Msh5**	Forms a heterodimer with **Msh4**, interacts with Mlh1/Mlh3 heterodimer. Directs Holliday junction resolution towards crossover with interference [119].
Msh2	Forms a heterodimer with Msh3 or Msh6 [119].
Msh6	Forms a heterodimer with Msh2, binds base-base mismatches [119].
Mlh1	Mismatch repair of dinucleotide and trinucleotide sequences, interacts with Msh2, forms heterodimers with Mlh2, Mlh3 and Pms1 [119].
Mlh2	Forms a heterodimer with Mlh1. Interacts with Msh2/3 or Msh2/6 for removal of cisplatin adducts [119].
Mlh3	Forms a heterodimer with Mlh1. Interacts with Msh2/3 or Msh2/6 for frameshift repair in mitosis or meiosis, or with **Msh4/5** to promote meiotic crossovers [119].
Pms1	Mismatch repair. Interacts with Msh2/3 or Msh2/6 as a heterodimer with Mlh1[119].
**Mer3**	Meiosis-specific DEAD-box helicase that promotes Holliday junction resolution with crossover interference together with ZMM proteins, including **Msh4** and **Msh5** [57], [58], [120]–[122].
Smc1	Forms a heterodimer with Smc3 to form core sister chromatid cohesin subunits, with ring shape around sister chromatids [123], [124].
Smc2	Forms a heterodimer with Smc4 to form core condensin subunits, ring shape, essential for chromosome assembly and segregation. [123]
Smc3	Forms a heterodimer with Smc1 to form core sister chromatid cohesin subunits, with ring shape around sister chromatids [123], [124].
Smc4	Forms a heterodimer with Smc2 to form core condensin subunits, ring shape, essential for chromosome assembly and segregation [123].
Smc5	Forms a heterodimer with Smc6 (Rad18) and is involved in DNA repair and checkpoint responses [123].
Smc6 (Rad18)	Binds ssDNA, has important role in postreplication DNA repair [125]. Forms a heterodimer with Smc5 and is involved in DNA repair & checkpoint responses [123].
Rad21 (Scc1)	Holds Smc1 and Smc3 heads together by binding N-terminal domain to Smc3 and C-terminal domain to Smc1, thus holding sister chromatids together during mitosis and meiosis [124].
**Rec8**	Meiotic homolog of Rad21. Holds Smc1 and Smc3 heads together by binding N-terminal domain to Smc3 and C-terminal domain to Smc1, thus holding sister chromatids together during meiosis [126].
Scc3	Necessary for sister chromatid cohesion, and required for DSB repair [127]. Interacts with Smc1, Smc3 and **Rec8**/Rad21 in holding cohesin ring together.
Pds5	Important for maintenance of sister chromatid cohesion in late prophase [127].

Genes encoding meiosis-specific proteins are highlighted in grey.

## Results and Discussion

We present our inventory of meiotic genes found in *T. vaginalis* in the context of an expanded set of 29 meiotic genes conserved among over 30 AFP and protist genomes (**Table 2**). Of the 29 meiotic genes surveyed, 27 have homologs in the *T. vaginalis* genome; homologs of the meiosis-specific sister chromatid cohesin Rec8 and the DNA repair protein Rad52 were not found. The 29 genes surveyed from the five of six major eukaryotic lineages [11] for which complete genome sequence data are available (Opisthokonta, ‘Amoebozoa’, ‘Archaeplastida’, ‘Chromalveolata’ and ‘Excavata’) include 17 genes previously reported as “core meiotic machinery” [1]. The large number of meiotic genes shared by *T. vaginalis*, mammals (*e.g.*, *Homo*) and fungi (*e.g.*, *Saccharomyces*) suggest that putative meiotic processes in *T. vaginalis* could resemble those in mammals and fungi. In contrast, *Giardia intestinalis*, the other putatively asexual early-branching protist in our study, lacks eight of 29 meiotic genes, and *Drosophila melanogaster*, a sexual organism, is missing ten. *G. intestinalis* was recently shown to utilize three of its meiosis-specific protein homologs (Spo11, Hop1 and Dmc1) to mediate homologous recombination (but not meiosis) in the nuclei of cysts in a process named diplomyxis [4]. Together, our data and these observations suggest that *T. vaginalis* may be equipped to perform meiotic recombination or similar parasexual process by using its meiotic gene homologs.

**Table 2:** Phylogenetic distribution among eukaryotes of core meiotic proteins and their prokaryotic homologs.

EUKARYOTES	Spo11	Mre11	Rad50	Rad1	Hop1	Hop2	Mnd1	Rad52	Dmc1	Rad51	Msh2	Msh6	Msh4	Msh5	Mlh1	Mlh2	Mlh3	Pms1	Mer3	Smc1	Smc2	Smc3	Smc4	Smc5	Rad18	Rad21	Rec8	Pds5	Scc3	
**Excavata**	***Trichomonas***	**S**	S	S(2)	S	**S**	**S(2)**	**S**	–	**S**	S(2)	S	S	**S**	**S**	S(3)	S(2)	S	S	**S**	P(2)	P	P(3)	P(2)	P(2)	S	S,P	**–**	S	S(2)
	***Giardia***	**S**	S	S	P	**S**	**P**	**S**	S	**S(2)**	–	S	S	**–**	**–**	S	S	–	S	**P**	P	P	P	P	B	S	–	**–**	–	–
	***Trypanosoma br/cr***	**P**	P	P	P	**P**	**P**	**P**	–	**P**	P	P	P	**P**	**P**	P	–	B	P	**P**	P	P	P	P	–	–	P	**–**	P	P
**Chromalveolata**	***Plasmodium fal/yoe***	**P(2)**	P	P	P	**P**	**P**	**P**	–	**P**	P	P(2)	P	**–**	**–**	P	–	–	P	**–**	P	P	P	P	P	P	P	**–**	–	–
	***Cryptosporidium p/h***	**P**	P	P	P	**P**	**P**	**P**	–	**P**	P	P	P	**–**	**–**	P	–	–	P	**–**	P	P	P	P	B	P	P	**–**	–	–
	*Tetrahymena*	**P**	P	P	P	**P**	**P**	**P**		**P**	P	P	P(4)	**B**		P			P			P	B	P						
	*Thalassiosira*	**P**	P	B	P			**P**	P		P	B	P	**B**	**P**	P			P		P	P	P	P		P	P		P	
	*Phytophthora ram/soj*	**P**	P	P				**P**	P	**P**	P	P	P			P			P		P	P	P	P		P	P		P	
**Amoe.**	*Dictyostelium*		P	P	P		**P**	**P**	P(2)		P	P	P	**P**	**P**	P		P	P		P	P	P	P	P	P	P		P	P
	***Entamoeba***	**P(2)**	P(2)	P	P	**–**	**P**	**P**	P(2)	**P(2)**	P	P	P	**P**	**P**	P	–	P	P	**–**	B	P	P	P	P	P(2)	P	**–**	–	P
**ANIMALS**	***Homo***	**P**	P	P	P	**P**	**P**	**P**	P	**P**	P	P	P	**P**	**P**	P	P	P	P	**P**	P(2)	P	P	P	P	P	P	**P**	P(2)	P(3)
	***Mus/Rattus***	**P**	P	P	P	**P**	**P**	**P**	P	**P**	P	P	P	**P**	**P**	P	P	P	P	**P**	P(2)	P	P	P	P	P	P	**P**	P(2)	P(3)
	*Gallus*		P	P	P	**P**		**B**	P	**P**	P	P	B	**B**	**B**	P	P	P	P	**P**	P	P	P	P	P	P	P(2)		P(2)	P(3)
	*Xenopus*	**P**	P	P	P	**P**	**P**	**P**	B		P	P	B			B	P	P		**P**	P	P	P	P	P	P	P	**B**	P(2)	P(2)
	*Tetraodon/Fugu/Danio*	**P**	P	P	P	**P**	**P**	**P**	P	**P**	P	P	P	**B**	**P**	P	P	P	P	**P**	P(2)	P	P	P	P	P	P(2)	**P**	P(2)	P(4)
	***Drosophila mel/pse***	**P**	P	P	P	**–**	**–**	**–**	–	**–**	P	P	P	**–**	**–**	P	–	–	P	**–**	P	P	P	P	P	P	P	**P**	P	P(2)
	*Anopheles*	**P**	P	P	P							P	P	**P**	**P**	P			P	**P**	P	P	P	P	P	P	P		P	P(2)
	***Caenorhabditis el/br***	**P**	P	P	P	**P**	**–**	**–**	–	**–**	P	P	P	**P**	**P**	P	–	–	P	**–**	P	P	P	P(2)	P	P(2)	P(2)	**P(3)**	P	P
**FUNGI**	***Saccharomyces***	**P**	P	P	P	**P**	**P**	**P**	P	**P**	P	P	P	**P**	**P**	P	P	P	P	**P**	P	P	P	P	P	P	P	**P**	P	P
	***Candida glabrata***	**P**	P	P	P	**P**	**P**	**P**	P	**P**	P	B	P	**P**	**P**	P	P	P	P	**P**	B	B	P	B	B	P	P	**P**	P	P
	***Kluyveromyces***	**P**	P	P	P	**P**	**P**	**P**	P	**P**	P	P	P	**P**	**P**	P	P	P	P	**P**	B	P	P	P	B	P	P	**P**	P	P
	***Candida albicans***	**P**	P	P	B	**P**	**P**	**P**	P	**P**	P	B	P	**P**	**P**	P	–	P	B	**P**	B	B	B(2)	B	B	B	P	**P**	P	P
	***Schizosaccharomyces***	**P**	P	P	P	**P**	**P**	**P**	P(2)	**P**	P	P	P	**–**	**–**	P	–	–	P	**–**	P	P	P	P	P	P	P	**P**	P	P(2)
	***Neurospora***	**P**	P	P	P	**–**	**–**	**–**	P	**–**	P	P	P	**–**	**P**	P	P	P	P	**P**	P	P	P	P	P	P	P	**P**	P	P
	***Gibberella***	**P**	P	P	P	**–**	**–**	**–**	P	**–**	P	P	P	**P**	**P**	P	P	P	P	**–**	P	B	B	P	P	B	P	**P**	P	P
	*Magnaporthe*	**P**	P		P				P		P	P	P	**P**	**P**	B	P	B	P	**P**	P	B	B	P	B	B	P	**P**	P	P
	***Aspergillus ni/fu/or***	**P**	P	P	P	**P**	**P**	**P**	P	**P**	P	P	P	**P**	**P**	P	P	P	P	**P**	P	P	P	P	P	P	P	**P**	P	P
	*Ustilago*		P	P	P				P		P	P	P	**P**	**P**	P		P	P	**P**	P	P	P	P	P	P	P	**P**	P	P
	*Cryptococcus*	**P**	P	P	P	**P**	**P**	**P**	P	**P**	B	P	P	**P**	**P**	P		P	P	**P**	P	P	P	P	P	P	P	**P**	P	P
	***Encephalitozoon***	**P**	P	P	P	**P**	**P**	**P**	P	**–**	P	P	P	**–**	**–**	P	–	–	P	**–**	P	P	P	P	P	P	P	**P**	P	P
**Archaeplastid.**	*Cyanidioschyzon*	**P**	P	P	P		**P**	**P**	P	**P**	P	P	P	**P**	**P**	P	P	P	P		B	B	B	B		P	P	**P**	P	P
	***Arabidopsis***	**P**	P	P	P	**P**	**P**	**P**	–	**P**	P	P	P(2)	**P**	**P**	P	–	P	P	**P**	P	P(2)	P	P	P	P(3)	P(3)	**P**	P(2)	P
	*Oryza/Zea*	**P**	P(2)	P	B	**P**	**P**	**P**	–	**P**	P(2)	P	P(2)	**B**	**P**	P	–	P	P	**P**	P	P	P(2)	P	P	P	P(3)	**P**	P(2)	P
	*Chlamydomonas*		P	P	P		**B**	**P**		**P**	P		P		**P**	P				**P**		P	P	P	B	P(2)	P		P	
**Homologs in:**																														
**ARCHAEA**		**Top6A**	SbcD	SbcC	Ercc4					RadA		MutS				MutL				**Ski2**	Smc									
**BACTERIA**										RecA																				

**Figure 2:**
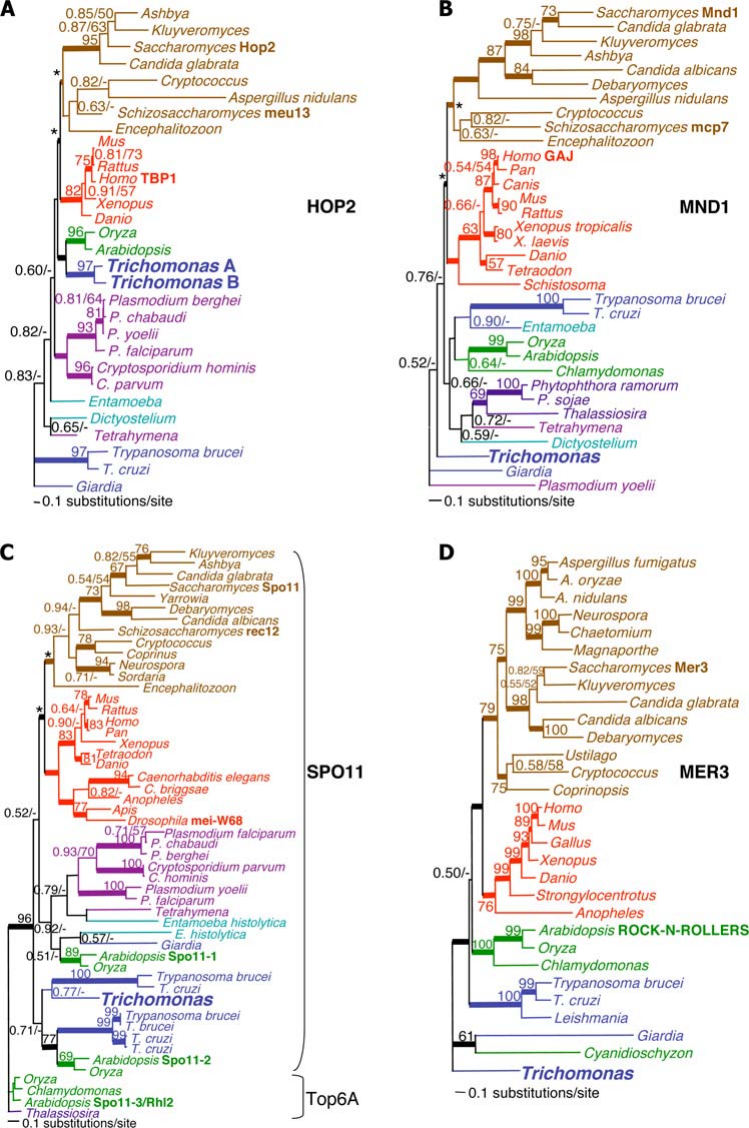
Phylogenetic trees for meiosis-specific proteins Hop2, Mnd1, Spo11 and Mer3. All trees shown are the consensus tree topologies determined from ≥700 best trees (*i.e.* those with the highest posterior probabilities) inferred by Bayesian analysis using alignments of inferred proteins. Animals are indicated in red text, fungi brown, ‘Amoebozoa’ teal, ‘Archaeplastida’ in green, Alveolates plum, ‘Chromista’ purple, ‘Excavata’ blue and prokaryotes shown in black. Branches with the best support – *i.e.*, those with 0.95 to 1.00 Bayesian posterior probabilities – have thicker lines. Numbers at the nodes indicate Bayesian posterior probability followed by percent bootstrap support from 100 replicates of PROML. An asterisk (*) denotes topological constraints placed upon the nodes uniting Fungi and Opisthokonts for Bayesian analysis. Scale bars represent 0.1 amino acid substitutions per site. Details for each tree and the accession numbers for all sequences are provided in Figures S1.1–S1.4 in Supporting Information File S1. (A) Hop2 homologs, unrooted. 167 aligned amino acid sites were analyzed, this consensus topology derived from 900 trees, α = 3.86 (2.71<α<5.37), pI = 0.014 (0.0004<pI<0.051) and lnL = −8363.01. (B) Mnd1 homologs, unrooted. 202 aligned amino acid sites were analyzed, this consensus topology derived from 850 trees, α = 2.80 (2.18<α<3.52), pI = 0.01 (0.0005<pI<0.043) and lnL = −11589.94. (C) Spo11 homologs, rooted with the eukaryotic Top6A paralog outgroup. 148 aligned amino acid sites were analyzed, this consensus topology derived from 700 trees, α = 1.76 (1.34<α<2.23), pI = 0.10 (0.03<pI<0.17) and lnL = −10624.08. (D) Mer3 homologs unrooted. 610 aligned amino acid sites were analyzed, this consensus topology derived from 950 trees, α = 1.60 (1.39<α<1.83), pI = 0.04 (0.02<pI<0.06) and lnL = −27086.67.

Bold columns highlight meiosis-specific proteins, and *T. vaginalis* is highlighted at the beginning. The names of genera with the most completely annotated genome sequences are highlighted in bold. The presence of orthologs is designated on the basis of phylogenetically verified data (P) obtained by BLASTp searches of public protein sequence databases and by cloning/sequencing (S) of selected genes (from *T. vaginalis*) in this study or our previous study [1]. The presence (B) of putative homologs identified by BLASTp from recently released sequence data and verified only by bi-directional BLAST searches (**Table S1.3 in Supporting Information File S1**) are also indicated. If more than one gene is present, copy number follows this designation parenthetically. The absence of meiotic genes from completed genome sequencing projects is indicated by (–), while empty cells indicate putatively missing data (from unfinished or incompletely annotated genomes). Protein homology was inferred by multiple sequence alignment and Bayesian phylogenetic analyses (see **Figure 2** **and Figures S1.1–S1.33 in Supporting Information File S1** for details), except for cells designated by “B”. Some data excluded from **Figures S1.1–S1.33 in Supporting Information File S1** were phylogenetically verified in equally or similarly rigorous prior studies [1], [50], [101], [102].

We found non-identical copies of eleven meiotic genes in *T. vaginalis* that are usually found as single copies in other eukaryotes (**Tables 2** **,** **3** and **Table S1.1 in Supporting Information File S1**). We cannot discern whether all of the copies are functional given the limited gene expression information available. However, our phylogenetic analyses show that these genes have evolved by recent duplications (within parabasalids) and, in most cases, one copy is more conserved than the others. Consistent with that observation, duplications within families of *Trichomonas vaginalis* protein-coding genes likely occurred after its divergence from sister taxon *Trichomonas tenax* [9]. The frequency of recently duplicated meiotic genes observed in *T. vaginalis* approaches that seen in some plants and fish (**Table 2**), which are both thought to have polyploid origins. Six of the eleven genes present in multiple copies in *T. vaginalis* (**Table 3**) are uniquely duplicated in *T. vaginalis* and not in any other organism included in our study. Duplications of chromosomes, segments of chromosomes, and possibly whole genomes as in some plants and fish might explain the presence of extra copies of meiotic genes in *T. vaginalis*. If so, these genes are putative homeologs or ohnologs – duplicated genes arising from polyploidization events [46]. Since the *T. vaginalis* genome sequence is highly repetitive and consists of 17,290 unordered scaffolds [9], additional data and analyses to better assemble the genome sequence into a smaller number of longer and ordered scaffolds are required to understand how chromosome- and genome-scale duplication contributed to its genome architecture. Ten of eleven recently duplicated meiotic genes were located on separate scaffolds. However, the two *Rad50* gene copies were found in an inverted tandem repeat on the same scaffold of assembled genome sequence, an arrangement which is inconsistent with polyploidization and that might result from ectopic meiotic sister chromatid recombination [47]. Our sequences from *T. vaginalis* strain NIH-C1 revealed that *Rad51b*, *Scc3b* and *Pds5* differed in the number of short tandemly repeated sequences within their coding regions when compared with the genome sequence of *T. vaginalis* strain G3. These duplicate genes (*Rad51b* and *Scc3b*) may also be derived from allelic divergence resulting from the accumulation of mutations during extensive asexual (mitotic) reproduction [48]. If this is the case, *T. vaginalis* may be facultatively sexual or asexual.

**Table 3:** Meiotic genes duplicated recently in *T. vaginalis*.

Gene name	# of copies	% nucleotide identity
***Hop2*** * (a, b)*	2	63%
*Rad50 (a, Ψ)*	2	44%
*Smc1 (a, b)*	2	45%
*Smc3 (a, b, c)*	3	53–54%
*Smc4 (a, b)*	2	56%
*Smc5 (a, b)*	2	53%
*Mlh1 (a, b, c)*	3	40–46%
*Mlh2 (a, Ψ)*	2	8%
*Rad51 (a, b)*	2	76%
*Rad21 (a, b)*	2	17%
*Scc3 (a, b)*	2	50%

Summarized from **Table S1.2 in Supporting Information File S1.** All duplicates trace to within parabasalids since their phylogenies show no intervening lineages, with three exceptions (*Smc1*, *Smc3*, *Mlh2*) that can be attributed to rapid rates of evolution.

To assess if the *T. vaginalis* meiotic genes are functional, we queried two expressed sequence tag (EST) databases and found evidence of the transcription of some genes (**Table S1.1 in Supporting Information File S1**). ESTs derived from normal asynchronized cultures were found for *Rad50a*, *Rad1*, *Dmc1*, *Msh2*, *Smc1b*, *Smc5b*, *Smc6*, *Rad21b* and *Pds5*. In low-iron conditions, *Msh5*, *Smc1a*, *Smc5a*, *Rad21b* and *Scc3a* are transcribed, while ESTs encoding *Rad51a* were found from G2/M trophozoites. Cells exhibiting vaginal epithelial cell mediated cytoadherence expressed *Smc2*, and ESTs encoding *Smc1b* and *Smc4b* were found from cold-induced pseudocysts (“compact non-motile forms without a cyst wall” [40]). These data are consistent with the expression of many (18 of 37) of the genes in our survey. However, the available ESTs may not represent conditions that promote meiosis, and their small numbers are consistent with the possibility that a given gene is expressed at low levels, or even post-transcriptionally down regulated. Of the 18 meiotic genes found in ESTs, *Dmc1* and *Msh5* are the only meiosis-specific gene orthologs found to be expressed in the available small sample of ESTs. The expression of *Dmc1* and *Msh5* orthologs suggests that meiotic recombination may occur in *T. vaginalis* in asynchronous cells and during low iron conditions (stress). In sum, the EST data are consistent with homologous recombination (and possibly meiosis) occuring in asynchronous cells, G2/M trophozoites and pseudocysts, as well as during low iron conditions and cytoadherence to vaginal epithelial cells.

### Phylogenetic inference of orthology and paralogy of meiotic proteins

Our revised inventory of meiotic proteins that spans an additional breadth of organisms with completely sequenced genomes allows us to elucidate the sexual status of basal eukaryotes. We have identified homologs of meiotic genes among organisms that may span most of the deepest divergences among eukaryotes by using phylogenetic inference to assess the evolutionary history of each of meiotic protein homolog. This approach allows evaluation of the origin and evolution of meiosis in the context of the common ancestor of eukaryotes (**Figure 2** and **Table 2**).

Many of the meiotic genes analyzed have homologs in prokaryotes while others are limited to eukaryotes (**Table 2**). There are prokaryotic homologs of 21 of the 29 meiotic genes (*i.e.*, *Spo11*, *Mre11*, *Rad50*, *Rad1*, *Dmc1*, *Rad51*, *Mer3*, *Msh2-6*, *Mlh1-3*, *Pms1*, *Smc1-6* are orthologs of prokaryotic *Top6a*, *SbcD*, *SbcC*, *Ercc4*, *RecA*, *Ski2*, *MutS*, *MutL*, and *Smc*, respectively). Of these, *Spo11*, *Dmc1*, *Rad51*, *Mer3*, *Msh2-6*, *Mlh1-3*, *Pms1*, and *Smc1-6* belong to multigene families that evolved from prokaryotic orthologs by gene duplication in eukaryotes; *Mre11*, *Rad50* and *Rad1* are non-duplicated genes with prokaryotic orthologs. Eight of the meiotic genes are apparently limited to eukaryotes (*i.e.*, *Hop1*, *Hop2*, *Mnd1*, *Rad52*, *Rad21*, *Rec8*, *Pds5*, *Scc3*) and either arose during eukaryotic evolution or diverged markedly beyond recognition from prokaryotic ancestors; all eight have experienced gene duplication events in their eukaryotic evolutionary histories. All 29 meiotic genes in our inventory are widespread among AFP and protists.

**Figure 2** highlights the phylogenies of four of eight meiosis-specific proteins found in *T. vaginalis*: Hop2, Mnd1, Spo11 and Mer3. *Hop2* and *Mnd1* homologs (**Figures 2A and 2B**) are apparently limited to eukaryotes. *Spo11* (**Figure 2C**) has a prokaryotic ortholog (*Top6A*) and evolved by early eukaryotic gene duplications [49]. *Mer3* (**Figure 2D**) also has a prokaryotic ortholog (*Ski2*) and belongs to a eukaryotic gene family of DEAD-box helicases. In these trees, the phylogenetic resolution of some groups is limited, but our analyses clearly demonstrate orthology of each gene (**Figure 2** and **Figures S1.1–S1.33 in Supporting Information File S1**) since *T. vaginalis* protein homologs consistently fall into groups that include proteins that were demonstrated to be meiosis-specific in AFP (as summarized in **Table 1**). In this study, we analyze a broader phyletic distribution of meiotic genes among eukaryotes than previously reported [1], [50].

In many cases, the broad survey of eukaryotes in this study enabled more precise identification of orthologous meiotic genes than previous smaller datasets permitted. The phylogeny of *Hop1* orthologs rooted by distant paralogs exemplifies this improvement. Hop1 is a meiosis-specific component of the synaptonemal complex. In contrast to previous results [1], the current analysis reveals the absence of a *Hop1* ortholog in *Drosophila*, *Anopheles* and *Neurospora* and the presence of a *Hop1* ortholog in *Encephalitozoon* and *Schizosaccharomyces* (**Table 2**). The absence of *Hop1* in these three sexual animal and fungal species demonstrates that meiosis is possible without it in animals and fungi, and possibly other organisms. In contrast, the presence of *Hop1* and other meiosis-specific genes (*Spo11*, *Hop2*, *Mnd1* and *Rec8*) in the putatively asexual microsporidian *Encephalitozoon* suggests that it may be sexual. A suite of meiosis-specific genes (*Hop1*, *Hop2*, *Mnd1* and *Dmc1*) apparently missing in *Drosophila*, *Anopheles* and *Neurospora* (all sexual organisms) was previously revealed as having a patchy phylogenetic distribution [1], [55]. However, our results show that these genes are generally conserved in most other major lineages of eukaryotes, and appear to be lost independently in different lineages, many which are known to be sexual. *Caenorhabditis*, nonetheless, is sexual and retains *Hop1* homologs but lacks *Hop2*, *Mnd1* and *Dmc1*. Interestingly, Hop2 and Mnd1 interact with Dmc1 to promote interhomolog recombination in *Mus* and *Saccharomyces* [51], [52]. The shared absence of *Hop1* from *Drosophila*, *Anopheles* and *Neurospora* suggests that Hop1 might also function with this suite of interacting proteins. For complexes of interacting proteins the evolutionarily conserved presence of the components suggests that their interactions are also conserved. One example of this principle is the universal presence in eukaryotes of both Mre11 and Rad50 that work together to mediate double strand break repair (**Table 2**).

Where previous comparative studies of meiotic genes have been taxonomically limited [1], [50], [53], [54], the distribution of meiotic gene homologs across the tree of eukaryotic life can be clarified by studying more diverse organisms. It is now clear that *Smc* homologs are ubiquitous among eukaryotes (**Table 2**). The *MutL* homologs, *Mlh2* and *Mlh3* are now demonstrably widespread, being found in AFP and protists. Although previous studies revealed few protist orthologs of *Msh4* and *Msh5* [1], [55] and suggested that the genes may have evolved recently, we find them in the genomes of several protists. *Mer3* orthologs are also widely present in AFP and protists, albeit sporadically. The presence of protist orthologs of *Msh4*, *Msh5* and *Mer3*, along with some apparent absences (**Table 2**, and Malik and Logsdon unpublished results) is readily explained by independent gene losses following the origin of paralogs by duplication. Notably, *Msh4*, *Msh5* and *Mer3* are concomitantly missing from *Plasmodium*, *Drosophila* and *Schizosaccharomyces*, although all are sexual. Msh4 and Msh5 interact as a heterodimer to promote resolution of meiotic Holliday junctions with crossover interference [56], in collaboration with ZMM proteins including Mer3 [57], [58]. While the presence of meiosis-specific *mutS* homologs *Msh4* and *Msh5* suggests the potential for meiosis in organisms such as *Trichomonas*, *Trypanosoma*, *Entamoeba*, *Arabidopsis*, *Homo* and *Saccharomyces*, the absence of *Msh4* and *Msh5* in sexual organisms indicates that meiosis can proceed without them. Thus, the presence of meiotic genes supports the hypothesis of sexuality, but the absence of a subset of genes does not exclude it.

Some meiotic genes in our survey exhibit patchy distributions (*i.e. Rad52*, *Msh4*, *Msh5*, *Mlh2*, *Mlh3*, *Mer3*, *Rad21*, *Rec8*, *Pds5* and *Scc3*, see **Table 2**). While these genes are present in many AFP and some protist lineages, they are absent from others, begging the question of whether these absences are due to recent gene loss. The conserved sister chromatid cohesin proteins Pds5, Scc3, Rec8 and Rad21 all interact in a complex with Smc1 and Smc3, which in turn are both present in each complete genome surveyed for this study. The absence of *Rec8*, *Pds5* and *Scc3* mainly among some protists suggests either that other proteins have evolved in these organisms to function with Smc1 and Smc3 during meiosis, or that orthologs of these genes have diverged beyond current recognition, as we previously thought for *Hop1* genes from *Encephalitozoon* and *Schizosaccharomyces* [1]. Conserved meiotic recombination genes (*e.g.*, *Rad52*, *Msh4*, *Msh5*, *Mer3*, *Mlh2*, *Mlh3*) may be missing in some eukaryotes because they were dispensable in the ancestors of those organisms, and perhaps replaced by alternate DNA repair machinery. However, the general conservation of meiotic genes among diverse eukaryotes is *prima facie* evidence that they are ancient and were present in the common ancestor of eukaryotes, even if they may be prone to lineage-specific losses or duplications during eukaryotic evolution.

### Evidence for meiosis in *T. vaginalis* and other eukaryotes

Although *T. vaginalis* is generally considered to be asexual, our inventory of meiotic genes suggests that the capacity for meiosis was present in the last common ancestor of *T. vaginalis* and other eukaryotes. Indeed, the presence of 27 of 29 components of the meiotic machinery in *T. vaginalis* suggests that the machinery for meiotic recombination was well established before the divergence of Parabasalids and Diplomonads (*e.g. Giardia*). It will be necessary to determine when in the *T. vaginalis* life cycle meiosis might occur and to discern if *T. vaginalis* uses standard two-step meiosis or a putative one-step meiosis (as was described in other Parabasalids [29], [30], [59]). Such one-step meiosis (if true; see refs. [8], [60]) has been suggested to be ancestral [28]–[30], [59], but could also represent a derivative form of two-step meiosis. Since meiosis in hypermastigotes is induced in response to an insect hormone, ecdysone [29], meiosis in *T. vaginalis* might be similarly induced in response to hormones of its animal hosts. Such conditions need to be explored in detailed cytological studies of cell division in trichomonads.

A goal in searching for meiotic gene homologs in *T. vaginalis* was also to expand the previous inventory of meiotic genes [1], [55] to other diverse eukaryotes. Organisms included in this expanded phylogenomic inventory of meiotic genes include some in which sexual cycles have not been observed [61]: *Cryptosporidium* (an alveolate), *Entamoeba* (an amoebozoan), *Cyanidioschyzon* (a red alga), *Encephalitozoon* (a microsporidian, derived from Fungi) and *Giardia* (a diplomonad). We found orthologs of meiosis-specific genes in the genomes of each of these organisms, indicating that they all may have the potential to undergo meiosis, or that they recently diverged from a sexual ancestor. Meiosis is well known among some alveolates such as *Plasmodium* and *Tetrahymena*, included here with *Cryptosporidium*. *Entamoeba* has homologs of six of nine meiosis-specific genes, suggesting that it may be able to initiate double-strand breaks and promote interhomolog recombination and Holliday junction resolution with crossover interference. Orthologs of *Mre11*, *Mlh3* and meiosis-specific *Hop2* genes in *Entamoeba* and *Dictyostelium*, and of meiosis-specific *Hop1* and *Msh4* genes in *Tetrahymena* were recently reported to be absent [62], [63] according to tBLASTx and other searches; however, our results of PSI-BLASTp searches and phylogenetic analyses revealed the presence of these genes. Red algae other than *Cyanidioschyzon* exhibit meiosis, as evident from 1.2 billion year old fossilized rhodophyte remains [64]. Homologs of several meiosis-specific genes, including *Spo11*, *Hop1*, *Hop2*, *Mnd1*, *Dmc1* and *Msh5*, were reported in the genome sequence of the green alga *Ostreococcus tauri*, suggesting that it has a hitherto undescribed sexual cycle [65]. *Encephalitozoon*, which is derived from a sexual lineage (Fungi [66]), has homologs of several meiosis-specific genes and only appears to be missing *Dmc1*, *Msh4* and *Msh5* among the meiosis-specific genes in our inventory. This may either indicate a hitherto unseen sexual cycle in *Encephalitozoon* or be representative of a secondarily asexual state, given that meiosis also occurs in some diplokaryotic microsporidia [67]. *Giardia intestinalis* was previously found to contain five of seven meiosis-specific genes surveyed [1]; we have determined here that *Giardia intestinalis* has one of two additional meiosis-specific genes (*Mer3*, but not *Rec8*). Recent analyses indicate that Diplomonads and Parabasalids are closely related [12]–[16]. The presence of homologs of some meiotic genes in *Giardia intestinalis* (and the absence of others) may suggest that the parasexual process in which some of these genes were recently shown to act [4] may represent an intermediate or primitive form of recombination that evolved prior to the origin of those missing meiotic genes. However, given the specific relationship of *Giardia* to *Trichomonas* and the presence of most of these meiotic genes in *T. vaginalis* (27 of 29), it is more likely that *Giardia intestinalis* secondarily lost some meiotic genes and its parasexual homologous recombination [4] is derived from a more typical meiotic recombination. In sum, this inventory of meiotic genes suggests the potential for meiosis in *Cryptosporidium*, *Entamoeba*, *Cyanidioschyzon*, *Encephalitozoon* and *Giardia*.

Finally, several other organisms among AFP and protists that are known to be sexual were also included in this inventory of meiotic genes (**Table 2**). Conserved homologs of meiotic genes are present in the stramenopiles *Thalassiosira* and *Phytophthora*. The conserved meiotic genes found in *Trypanosoma brucei* and *Trypanosoma cruzi* and the recently discovered evidence for genetic exchange *in vitro* in these organisms [68], [69] together support evidence for a sexual cycle. We also find that the meiotic genes in *Saccharomyces* are conserved in other fungi, though *Gibberella* (and possibly, *Magnaporthe*) is missing the same set of genes that are also absent in its close relative, *Neurospora*. The meiotic genes found in mammals are generally conserved in other vertebrates, as well as invertebrates. Notably, genes such as *Mnd1* and *Dmc1* that are found in vertebrates but missing in *Drosophila* and *Caenorhabditis* are present in *Schistosoma* and *Bombyx*, which suggest multiple independent lineage-specific losses of these genes during the evolution of animals. Plants and the green alga *Chlamydomonas* included in our analysis also appear to share a similar complement of meiotic genes.

Of the nine meiosis-specific genes included in our study, only *Rec8* —the meiosis-specific paralog of the *Rad21* cohesin— cannot be found in any protists (**Table 2**). Additional data from other protist lineages will be required to ascertain when *Rec8* and *Rad21* diverged. In any case, Rad21 may perform the meiotic role of Rec8 for homologous chromosome cohesion in sexual protists: although Rad21 is not meiosis-specific, it has a critical meiotic role, which in the absence of Rec8 may be sufficient for meiosis [70], [71].

### Conclusions

We found 27 of 29 meiotic genes in *Trichomonas vaginalis*, and 21 of these 29 genes are also present in *Giardia intestinalis*. These 27 meiotic genes must have been present in the common ancestor of *Trichomonas* and *Giardia*, and given the highly diverged positions of these lineages among eukaryotes [12]–[16], each of the genes also must have been present in the common ancestor of all eukaryotes. The conservation of this inventory of meiotic genes across such a diverse group of sexual and putatively asexual eukaryotes allows us to infer that the presence of these genes – particularly the meiosis-specific genes – in putatively asexual eukaryotes indicates the potential for meiosis, at least in their recent ancestors.

The widespread presence of the meiotic genes indicates that the core meiotic machinery is largely universal among extant eukaryotes. Our results show that a substantial fraction of the meiotic machinery has evolved early in eukaryotes (**Table 2**). The evolution of each of the components of the meiotic machinery early during eukaryotic evolution implies that the interactions among the proteins included in our inventory also predate the divergences of the organisms included here. The proteins inventoried here are involved in creating meiotic double-strand DNA breaks and in subsequent meiotic DNA repair, crossing over, and cohesion of sister chromatids and homologous chromosomes, which when found together are compelling evidence for their potential interaction in processes resembling meiotic recombination. We can use this inventory of conserved meiotic genes as a “meiosis detection toolkit” with which to look in the genomes of putative asexuals for homologs of the meiotic machinery. This makes useful *a priori* data with which to further investigate the occurrence of sexual or parasexual processes in the life cycles of organisms for which sex has not been observed, which may have important ecological and epidemiological implications for some organisms such as abundant or parasitic eukaryotic microorganisms.

## Materials and Methods

### Database mining

Searches through the literature and keyword searches of the National Center for Biotechnology Information (NCBI) protein database revealed homologs of 29 meiotic proteins from various organisms. These protein sequences were used as queries for BLASTp, PSI-BLASTp and tBLASTn searches [72] of the NCBI nonredundant and genomic sequence databases between October 2003 and May 2006. Similarly, meiotic protein homologs were retrieved from the protist genome sequence databases of *Giardia intestinalis* ([73], [74], http://www.mbl.edu/Giardia), *Trypanosoma brucei* [75], *Trypanosoma cruzi* [76], *Entamoeba histolytica* [77], *Tetrahymena thermophila* [78] and *Trichomonas vaginalis* strain G3 [9] at The Institute for Genomic Research (TIGR, www.tigr.org/tdb/euk), *Cyanidioschyzon merolae* ([79], http://merolae.biol.s.u-tokyo.ac.jp/blast/blast.html), and *Thalassiosira pseudonana* [80], *Phytophthora ramorum* and *Phytophthora sojae* [81] and *Chlamydomonas reinhardtii* [82] at the Joint Genome Institute (JGI, http://genome.jgi-psf.org/) by either BLAST or keyword searches of annotated proteins. Between November 2003 and May 2006, unannotated nucleotide sequences of meiotic genes were extracted from the genome of *Trichomonas vaginalis* strain G3 [9] by tBLASTn searches of the database of unannotated sequences (http://tigrblast.tigr.org/er-blast/index.cgiprojecttvg) with meiotic protein homologs from other eukaryotes as the queries. Once the sequences for each *T. vaginalis* gene (**Table 2**) were mined from the database, putative start and stop codons were inferred on the basis of the inferred translation with reference to pairwise comparisons (BLASTx of GenBank) and multiple sequence alignments of homologous proteins. Sequences were assembled and putative open reading frames annotated using Sequencher™ 4.6 (Genecodes, Ann Arbor MI, USA). We used meiotic gene homologs from *T. vaginalis* strains NIH-C1 or G3 as queries to search public databases of *T. vaginalis* strain G3 expressed sequence tags (ESTs) by BLASTn (http://cgbc.cgu.edu.tw/est/, now at http://www.trichdb.org/trichdb) and dbEST at NCBI http://www.ncbi.nlm.nih.gov/blast/) in August and October 2005 using BLASTn e-value cutoffs zero to −185. *T. vaginalis* meiotic genes were also mapped back to whole genome shotgun (WGS) scaffolds at NCBI by BLASTn in August 2005. Vertebrate orthologs of Mer3 were identified by BLASTp searches of NCBI in May 2007. Pairwise comparisons of the nucleotide sequences and inferred translations of *T. vaginalis* duplicated genes were made using the LAlign and PRSS3 programs (http://www.ch.embnet.org/software/LALIGN_form.html [83] and http://www.ch.embnet.org/software/PRSS_form.html [84], [85]).

### PCR amplification

Amplification primers designed from the *Trichomonas vaginalis* strain G3 genome project sequences were used to amplify products for regions including the entire gene of interest and roughly 100–200 nucleotides of flanking sequence on either end when possible (**Table S1.2 in Supporting Information File S1**). In some cases, gene fragments were discovered in the first release of the genome (November 2003) and PCR was used to link the fragments together. Miklós Müller (Rockefeller University, New York) generously provided genomic DNA from *Trichomonas vaginalis* strain NIH-C1. Genes were amplified from this DNA by PCR with Eppendorf MasterTaq™ DNA polymerase (Hamburg, Germany) or Stratagene Easy-A™ DNA polymerase (La Jolla CA, USA), as recommended by the manufacturers, with 10–50 ng DNA, 250 µM each dNTP (Invitrogen, Carlsbad CA, USA) and 1 µM each primer (synthesized at Integrated DNA Technologies (IDT, Coralville IA, USA)) per reaction. Reaction conditions were 95°C for 3 minutes followed by 35–40 cycles at 92°C for 40–90 seconds, 35–55°C for 60–90 seconds and 72°C for 90–120 seconds+6 seconds/cycle, then ending at 72°C for 5–7 minutes. PCR products were fractionated and isolated from 1% low melt: 1% NuSieve™ GTG agarose (Fisher [Pittsburgh PA] and BioWhittaker [Walkersville MD]) in 1× TAE buffer. DNA bands were excised from the gel and cloned directly into the pCR4.0-TOPO™ vector (Invitrogen) according to the manufacturer's instructions. PCR screening with T3 *vs* T7 primers was used to identify putative clones by the size of their plasmid inserts, cycling at 94°C for 2 minutes followed by 30 cycles at 94°C for 1 minute, 57°C for 1 minute and 72°C for 2–3 minutes, then ending at 72°C for 5 minutes (reagents from Invitrogen, Promega [Madison WI, USA] and Fisher) [86]. At least two clones per PCR product were isolated (Eppendorf FastPlasmid Kit™) and sequenced (ABI BigDye™ 3.1 and ABI 3730™, Applied Biosystems, Foster City CA, USA) on both strands using M13 forward/reverse and gene-specific primers (Invitrogen and IDT). All sequences have been deposited in GenBank, accession numbers DQ321757–DQ321785 and DQ485348, as listed in **Table S1.2 in Supporting Information File S1**. We used the TIGR database predicted translations of July 2005 for *Smc1*, *Smc2*, *Smc3*, *Smc4* and *Smc5* homologs from *T. vaginalis* strain G3 for our analyses and did not sequence these from strain NIH-C1.

### Phylogenetic analysis

Phylogenetic inference of the evolutionary relationships of each set of putative meiotic proteins present in *T. vaginalis* and its homologs obtained from public databases was used to assign orthology to the *T. vaginalis* meiotic protein homologs. Multiple alignments of amino acid sequences from complete proteins were initially constructed using ClustalX 1.83 [87], then inspected and adjusted manually using MacClade 4.08 [88]. Only unambiguously aligned amino acid sites were used for phylogenetic analyses. For the alignment of eukaryotic and prokaryotic MutS homologs, sites were selected with GBLOCKS ([89], http://molevol.ibmb.csic.es/Gblocks_server/index.html). Phylogenies are unrooted and also rooted by outgroups when possible using either non-meiotic paralogs in a eukaryotic multigene family, or prokaryotic orthologs. Additional analyses in which systematically problematic sequences were removed were also performed (not shown).

MrBayes3.0b4 [90], [91] was used for analyses of each meiotic protein alignment. MrBayes was run for 10^6^ generations, with four incrementally heated Markov chains, a sampling frequency of 10^3^ generations and the temperature set at 0.5. Among-site substitution rate heterogeneity was corrected using an invariable and eight gamma-distributed substitution rate categories and the WAG model for amino acid substitutions [92], abbreviated herein as WAG+I+8G. The consensus tree topology, the arithmetic mean log-likelihood (lnL) for this topology, and branch support were estimated from the set of sampled trees with the best posterior probabilities. The number of trees included in this set varied among analyses. Means and 95% confidence intervals for the gamma distribution shape parameter (α) and the proportion of invariable sites (pI) were also estimated for each alignment that was analyzed. These analyses were repeated in MrBayes3.1.2 for the Hop2, Mnd1 and Spo11 datasets with two topological constraints that group fungi and opisthokonts (animals+fungi). Bootstrap support for the Hop2, Mnd1, Spo11, Mer3 and Msh datasets was estimated with PROML (with SEQBOOT and CONSENSE in PHYLIP 3.6a3 [93]) for 100 bootstrap replicates using the JTT substitution model [94] and eight categories of gamma-distributed and invarying sites (abbreviated herein as JTT+I+8G), with the coefficient of variation calculated from the alpha parameter estimated by MrBayes3.0b4 for each dataset. Prior to the Bayesian analyses shown (**Figure 2** and **Figures S1.1–S1.33 in Supporting Information File S1**), preliminary analyses (results not shown) were carried out using parsimony and distance methods for the purposes of monitoring the progress of the project and for examining partial sequence data (PAUP*4.0b10 [95] and SEQBOOT, PROTDIST, NEIGHBOR and CONSENSE in PHYLIP 3.6a3 [93]). Using Tree-Puzzle 5.2 [96] we generated maximum-likelihood distance matrices in which among-site substitution rate heterogeneity was corrected using the JTT+I+8G model (results not shown). Neighbor-joining trees were constructed using NEIGHBOR.

## Supporting Information

Supporting Information File S1 (0.96 MB PDF)
