# Correction: An Expanded Inventory of Conserved Meiotic Genes Provides Evidence for Sex in *Trichomonas vaginalis*

**DOI:** 10.1371/annotation/029c403b-10aa-47af-9e99-11451de76b85

**Published:** 2008-09-05

**Authors:** Shehre-Banoo Malik, Arthur W. Pightling, Lauren M. Stefaniak, Andrew M. Schurko, John M. Logsdon

In the last two rows of Table 2, the data appear in the wrong columns. Please view the corrected table here: http://www.plosone.org/corrections/pone.0002879.t002.cn.pdf

