# Supplementary material for: An Expanded Inventory of Conserved Meiotic Genes Provides Evidence for Sex in Trichomonas vaginalis
Source: PLoS One. 2008 Aug 6;3(8):e2879. doi: 10.1371/journal.pone.0002879 (PMC2488364; doi:10.1371/journal.pone.0002879)
Supplement: Supporting Information File S1 — (0.96 MB PDF) [file pone.0002879.s001.pdf]

Supporting Information for:

An expanded inventory of conserved meiotic genes provides evidence for sex in *Trichomonas vaginalis*

Shehre-Banoo Malik[1,2], Arthur W. Pightling[1], Lauren M. Stefaniak[1,3], Andrew M. Schurko[1] & John M. Logsdon, Jr.[1,4]

[1]Department of Biology
Roy J. Carver Center for Comparative Genomics
University of Iowa
Iowa City, IA USA 52242-1324

[2]Current address:
Department of Medical and Molecular Parasitology
New York University School of Medicine
341 East 25th Street
OPH Building Room 217
New York NY USA 10010-2598

[3]Current address:
Department of Marine Sciences
University of Connecticut
Groton, CT USA 06340

[4]Author for correspondence:
Email: john-logsdon@uiowa.edu
FAX: 319-335-1069; TEL: 319-335-1082

e-mail addresses for all authors:
shehre-banoo.malik@nyumc.org
arthur-pightling@uiowa.edu
lauren.stefaniak@uconn.edu
andrew-schurko@uiowa.edu
john-logsdon@uiowa.edu

Running title: **Meiosis genes in *Trichomonas***

Supporting Information

Table S1.1: Comparisons of *T. vaginalis* meiotic genes with ESTs, among duplicates, and with *Saccharomyces* orthologs. *T. vaginalis* nucleotide sequences from this study (strain G3) are compared with ESTs and Genbank WGS from strain NIH-C1, and comparisons made among gene duplicates. Inferred translations are compared with *Saccharomyces* proteins. Meiosis-specific genes are highlighted in grey. Cell populations for ESTs from http://cgbc.cgu.edu.tw/est/ (now http://www.trichdb.org/trichdb) are identified as TvE = normal unsynchronized culture, TvLI = low iron culture, TvG = G2/M trophozoite, TvC = cold-induced pseudocyst, TvV = vaginal epithelial cell mediated cytoadherence. Both amino acid and nucleotide sequences of duplicate genes were compared.

Table S1.1.

Gene	identity of strain NIH-C1 *vs* TIGR/WGS strain G3 (GI#)	5'-3' WGS strain G3 coordinates	aa identity to yeast	matching strain G3 EST (GI or clone#)	identity to "homeolog" a.a. (nt.)	Probability of random alignment (PRSS)
Spo11	99% (66650271)	14495-15649	24%	n/a	n/a	
Mre11	99% (66649716)	10936-12624	25%	n/a	n/a	
Rad50a	99% (66651528)	52907-49258	26%	52151037, 52151036	37% (44%)	3.087 e-20 (3.728 e-21)
Rad50bψ	100% (66651528)	38884-41151	23%	n/a		
Rad1	99% (66651345)	40355-41997	28%	TvE143B03	n/a	
Hop1	100% (66651530)	11527-10544	29%	n/a	n/a	
Hop2a	99% (66651652)	65469-64798	21%	n/a	62% (63%)	5.653 e-14 (1.791 e-14)
Hop2b	99% (66651263)	926-174	26%	n/a		
Mnd1	100% (66651572)	13392-12775	28%	n/a	n/a	
Rad51a	100% (66648917)	5593-6582	62%	TvG004A11	82% (76%)	1.253 e-16 (2.158 e-19)
Rad51bψ	97% (66651508)	52204-51212	64%	n/a		
Dmc1	99% (66650366)	9441-8425	53%	TvE134E05	n/a	
Msh2	99% (66649410)	5815-3260	31%	TvE115E07	n/a	
Msh4	99% (66651026)	27063-28729	23%	n/a	n/a	
Msh5	99% (66649326)	1224-3530	28%	TvLI170G03	n/a	
Msh6	99% (66651668)	108481-105549	29%	n/a	n/a	
Mlh1a	99% (66651275)	13639-10943	30%	n/a	22-31% (40-46%)	
Mlh1b	99% (66650799)	4560-6941	28%	n/a		
Mlh1c	99% (66651671)	107169-105106	24%	n/a		
Mlh2a	99% (66651015)	28847-27459	26%	n/a	14% (8%)	4.604 e-15 (4.319 e-2)
Mlh2bψ	99% (66651592)	38497-36643	26%	n/a		
Pms1	99% (66649895)	12908-14577	29%	n/a	n/a	
Mlh3	99% (66648511)	5757-4373	33%	n/a	n/a	
Mer3	99% (66649372)	1181-3877	33%	n/a	n/a	
Smc1a	n/a (66650970)	20649-23510	24%	TvLI142A02	17% (45%)	1.499 e-16 (4.352 e-05)
Smc1b	n/a (66650109)	7300-10656	23%	TvC128A03,TvE050F12		
Smc2	n/a (66650746)	12261-8752	31%	TvV057H06	n/a	
Smc3a	n/a (66650923)	8546-11953	25%	n/a	25-30% (53-54%)	
Smc3b	n/a (66651268)	9173-12640	26%	n/a		
Smc3c	n/a (66650705)	12278-8991	23%	n/a		
Smc4a	n/a (66651502)	17016-20549	30%	n/a	35% (56%)	1.321 e-20 (1.914 e-12)
Smc4b	n/a (66648681)	2200-5808	31%	TvC041E10		
Smc5a	n/a (66651662)	104193-107447	25%	TvLI132C07	29% (53%)	1.126 e-27 (3.279 e-9)
Smc5b	n/a (66649198)	7991-4830	23%	TvE100G07		
Smc6	99% (66650850)	14523-17637	23%	TvE085E04, TvE046G10	n/a	
Rad21a	99% (66649047)	4063-5232	25%	n/a	18% (17%)	1.56 e-10 (7.755 e-3)
Rad21b	99% (66651520)	47904-46444	23%	TvE199G05,TvLI086H02, TvLI008D09		
Pds5	99% (66651004)	28656-25033	24%	TvE118B03	n/a	
Scc3a	99% (66651519)	19579-22293	23%	TvLI146B03	19% (50%)	5.817 e-41 (3.186 e-4)
Scc3b	99% (66650490)	18831-21434	19%	n/a		

Table S1.2: Primers designed from strain G3 and used for PCR amplification of *Trichomonas vaginalis* strain NIH-C1 meiotic genes and corresponding NCBI GI numbers for genes sequenced in this study. Meiosis-specific genes highlighted in grey.

Gene Name	NCBI GI#	Forward Primer (5' – 3')	Reverse Primer (5' – 3')
Spo11	DQ321760	F1: TTACTCAGGTTTACGAC<br>F2: CTCATAGATCAACAGCAGC	R1: TTTAACTCAGACGTC<br>R2: CGATGTTATCATTGGATG
Mre11	DQ321776	GGAAAGGTGACCATC	CTGGCGTGAAGCGTC
Rad50a	DQ321780	F1: GGCAGTTTTATCCATGTAG<br>F2: GATCTCAAGTCAAAATCCG<br>F3: GTATTAAGAAAGCTACG	R1: GGATAGTGATCATATGAG<br>R2: AACTAGATGTAGACA<br>R4: CACTTCTTAGTTCAGCATC
Rad50bψ	DQ321781	TAACAGTGAAGGGTATTCG	CTGTAGATCTTGGGATGCC
Rad1 (Mei9)	DQ321779	F1: CTCTTTGGTTGATTGGCTC<br>F2: TCCAAAGCCACAACAGTG	R1: TGTGGAGCAAATCGACCAC<br>R2: GATCACTATATGCTTTGCGC
Hop1	DQ321759	GAGTCTTTCAATCAGC	CGAGTTAATCATTCCAGG
Hop2a	DQ321770	GAATCCAAAGGGAAG	GAAGGTCCTATTCAAG
Hop2b	DQ321771	GATATGGCATAATTTGGC	GCAGTTCAATCAATG
Mnd1	DQ321773	GCGATCCTAAGAAACTC	GCTGGATGAGAAATGGC
Dmc1	DQ321768	AGGGTCCACGAGCAT	TACTCATGCAGCAGA
Rad51a	DQ321774	ACTGAGTCGATAGCTC	CTCTTCCGGTATGAC
Rad51bψ	DQ321775	F1: TGCTATGAACAATGA<br>F2: GGAACTTGGTTTACC	R1: CTATCTGCGCTATAC<br>R2: GGTGAATTTCAATTCGG
Msh2	DQ321769	F1: TGAAGGGGTAAACACGAG<br>F2: CCAAGCAAGACATCCAATCC	R1: GAAATCAAAGCAGCAATGG<br>R2: CGAATCCAACATCGTAAA
Msh4	DQ321778	TCGTCAGAGAATTGGCAG	GCTGCATCAGCGATGACAT
Msh5	DQ321757	F1: GACTTCCATCAAAGAAGG<br>F2: ATCAATGTCAGATAGAGG	R1: GGACATTGGATCAGACCA<br>R2: CGTTATCGGAGCGATTAT
Msh6	DQ321777	F1: GACTATTTGATAGGCAAGG<br>F2: CAATTGCAGCCTTAGGTGG	R2: CAGGAATTGTCTTGAGCTCC<br>R3: CCGAATGAGCTTGGACAC
Mlh1a	DQ321764	F1: ATCGAATTCTCCAGCTCG<br>F2: GGGAACTACGATTATTTGAAGG<br>F3: GGGATGTTTAGAAGAGATGCCG	R2: GCATTCCAACTCTCCATCCC<br>R3: CGCAAATGTAACTGATGC
Mlh1b	DQ321783	TGACGCATATTGGTGTGC	GCTTGCATATGAACATCA
Mlh1c	DQ321784	CAGATATGATCAGAATCC	GTCGAAGTTATGGAAGCG
Mlh2	DQ321765	GGAATAATGACAAACACC	GCAATTGAATCCATGTGC
Mlh2bψ	DQ321782	CACTAAGTGATAGGTTGG	GGGAATCTCTTGTTGACC
Mlh3	DQ321766	CTTGCATCTATATGAATAC	GGCACAGCAGCATAAGTGC
Pms1	DQ321767	ACTAGGAGCACACTTACC	CTTCCGTGCGGACAATTCC
Mer3	DQ485348	GAATTCGATTAAGGTGGAG	R1: TAGTTGAGATGGCAAGGAC<br>R2: CATAATTTGGAAGCCAAGC
Rad18 (Smc6)	DQ321772	F1: GGGAACAAGACGTTGTGG<br>F2: GTGGATGATATAGAC<br>F3: CTAGATACGACATTGGC	R1: AGCCAAGATAATGCC<br>R2: GGTCATAATGGAGTC<br>R3: CCATGTCCTGTCCGCTG
Rad21a	DQ321758	F7: TACAAGATTCTCGGCATC<br>F8: GCCATGAATTTTCCAGCAAC	R11: GCATTTCTCCATACGATG<br>R12:GAGCAATTAGACATTGCGAG
Rad21b	DQ321785	F4: TGAACCAGTTGATCTCGCTGA	R4: CTGGTAGAAAGCTCTTGC
Pds5	DQ321761	F1: CACGAAGTGCCTGGAAGCC<br>F2: AAGATGTTGATTTGCTCTG<br>F3: AACCTATGCCAGCCTTACC	R1: AGAACGTGGCATCCCAG<br>R2:GTAAGTTTACAGAACGTGGC<br>R3:AGTGCTTTAAGGACATCTGG
Scc3a	DQ321762	F1: GAAATCCGAATGAGCATCC<br>F2: TTGCAGAGCTTTGCTCCG	R1: TTTCACTGCTACGTTCGCC<br>R2: CTGGAACTGACTTATGATCC
Scc3b	DQ321763	F1: TTCTTCATAGTGTCGAGAG<br>F2: AGTGTAAGAATCGCTGCTC	R1: CTTATGCTTGTCCAGCTGG<br>R2: ATCTTACCTGATGCTGCC

Table S1.3: Meiotic genes identified by BLASTp of public databases and verified reciprocally by BLASTp of NCBI. These data are from September 2006, after our phylogenetic analyses were completed.

Group	Organism	Protein	Database	Accession #
Animals	*Xenopus laevis*	Rad52	NCBI	GI# 68533761
		Msh6	NCBI	GI# 58399508
		Mlh1	NCBI	GI# 89269512
		Rec8	NCBI	GI# 56269132
	Gallus gallus	Msh4	NCBI	GI# 50751778
		Msh5	NCBI	GI# 50798458
		Msh6	NCBI	GI# 50739008
	Danio rerio	Msh4	NCBI	GI# 68388163
	Tetraodon	Msh4	NCBI	GI# 47230170
Fungi	*Candida albicans*	Rad1	NCBI	GI# 68477617
		Msh2	NCBI	GI# 68481114
		Pms1	NCBI	GI# 68491028
		Smc1	NCBI	GI# 46436700
		Smc2	NCBI	GI# 46436033
		Smc3a	NCBI	GI# 46441945
		Smc3b	NCBI	GI# 46441807
		Smc4	NCBI	GI# 68485641
		Smc5	NCBI	GI# 68485641
		Smc6	NCBI	GI# 46442783
	Candida glabrata	Msh2	NCBI	GI# 50290307
		Smc1	NCBI	GI# 50287267
		Smc2	NCBI	GI# 50286419
		Smc4	NCBI	GI# 50293773
		Smc5	NCBI	GI# 50287189
	Kluyveromyces lactis	Smc1	NCBI	GI# 50306843
		Smc5	NCBI	GI# 50310839
	Gibberella zeae	Smc2	NCBI	GI# 46121453
		Smc3	NCBI	GI# 46124753
		Smc6	NCBI	GI# 46136645
	Magnaporthe grisea	Mlh1	NCBI	GI# 14029392
		Mlh3	NCBI	GI# 39942408
		Smc2	NCBI	GI# 39971565
		Smc3	NCBI	GI# 39940504
		Smc5	NCBI	GI# 86196520
		Smc6	NCBI	GI# 39942986
	Cryptococcus neoformans	Rad51	NCBI	GI# 57223153
Amoebozoa	*Entamoeba histolytica*	Smc1	NCBI	GI# 67482463
Viridiplantae	*Oryza sativa*	Rad1	NCBI	GI# 108705675
		Msh4	NCBI	GI# 113611196
		Mer3	NCBI	GI# 113536973
	Chlamydomonas reinhardtii	Hop2	JGI-Chlre3	1747501|fgenesh2.pg.c.scaffold.26000132
		Mer3	JGI-Chlre3	142899|Chlre2.kg.scaffold4000022
		Smc5	JGI-Chlre3	122088|e_gwW.56.45.1
Red algae	*Cyanidioschyzon merolae*	Smc1		CMI192C
		Smc2		CMG189C
		Smc3		CML027C
		Smc4		CME029C
		Smc6		CMA066C
Chromalveolates	*Cryptosporidium parvum*	Smc5	NCBI	GI# 66359718
	Tetrahymena thermophila	Msh4	NCBI	GI# 89303698
		Smc3	NCBI	GI# 89294880
	Thalassiosira pseudonana	Rad50	JGI-thaps1	18982|newV2.0.genewise.222.1.1
		Msh2	JGI-thaps1	20930|newV2.0.genewise.25.224.1
		Msh4	JGI-thaps1	119515|newV2.0.genewise.13.54.1
Kinetoplastids	*Trypanosoma brucei*	Mlh3	TIGR	Tb927.5.2020
Diplomonads	*Giardia intestinalis*	Smc5	NCBI	GI# 29250412

Figures S1.1-S1.33: ***Phylogenetic trees of eukaryotic meiotic proteins listed in Table 2.*** All trees shown are the consensus Bayesian ML tree topologies derived from the ≥ 400 trees with the highest posterior probabilities inferred by Bayesian analysis using alignments of inferred proteins. In general, proteins are labeled according to their names assigned in *Saccharomyces cerevisiae*. Animals are indicated in red text, fungi in brown, 'Amoebozoa' teal, 'Archaeplastida' in green, Alveolates plum, 'Chromista' purple, and 'Excavata' in blue; prokaryotes are shown in black. Numbers at nodes represent Bayesian posterior probabilities for that relationship. An asterisk (*) indicates constrained nodes. The scale bars represent 0.1 amino acid substitutions per site. Accession (NCBI GI) numbers for all sequences are provided alongside the taxon names. See Materials and Methods for details on analytical methods. Meiosis-specific proteins shown are Spo11, Hop1, Hop2, Mnd1, Dmc1, Mer3, Msh4, Msh5 and Rec8. **Figures S1.1-S1.4** are also shown as **Figure 2 (A – D)** in the main text.

Figure S1.1: Hop2 homologs, unrooted, with Fungi and Opisthokonts constrained. 167 aligned amino acid sites were analyzed, this consensus topology derived from 900 trees, $\alpha = 3.86$ $(2.71 < \alpha < 5.37)$, $pI = 0.014$ $(0.0004 < pI < 0.051)$ and $lnL = -8363.01$. (Same as **Figure 2A**) For the unconstrained analysis, see **Figure S1.18**.

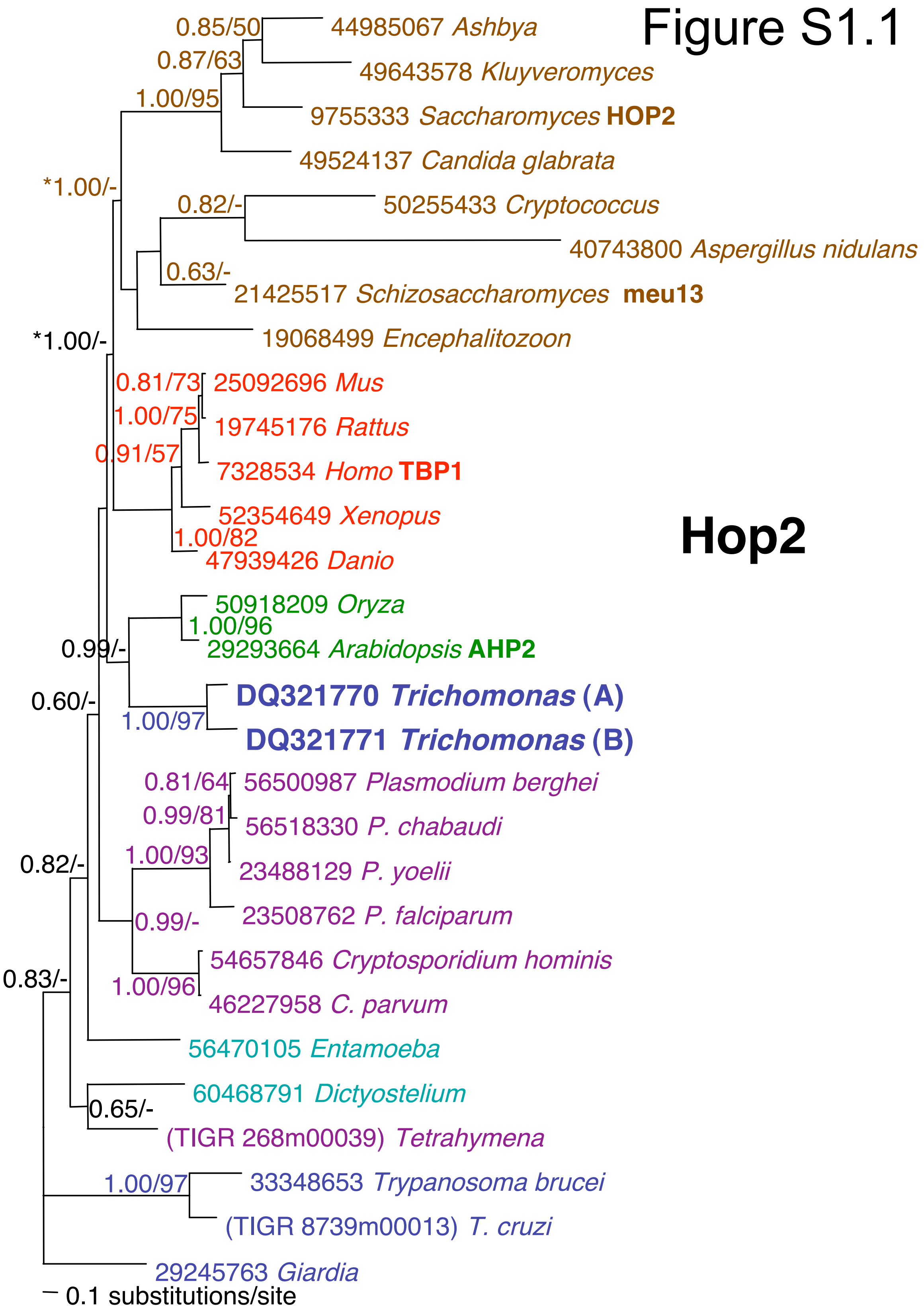

Figure S1.1
Hop2
0.85/50
44985067 Ashbya
0.87/63
49643578 Kluyveromyces
1.00/95
9755333 Saccharomyces HOP2
49524137 Candida glabrata
*1.00/-
0.82/-
50255433 Cryptococcus
40743800 Aspergillus nidulans
0.63/-
21425517 Schizosaccharomyces meu13
19068499 Encephalitozoon
*1.00/-
0.81/73
25092696 Mus
1.00/75
19745176 Rattus
0.91/57
7328534 Homo TBP1
52354649 Xenopus
1.00/82
47939426 Danio
50918209 Oryza
1.00/96
29293664 Arabidopsis AHP2
0.99/-
DQ321770 Trichomonas (A)
0.60/-
1.00/97
DQ321771 Trichomonas (B)
0.81/64
56500987 Plasmodium berghei
0.99/81
56518330 P. chabaudi
1.00/93
23488129 P. yoelii
0.82/-
23508762 P. falciparum
0.99/-
54657846 Cryptosporidium hominis
0.83/-
1.00/96
46227958 C. parvum
56470105 Entamoeba
60468791 Dictyostelium
0.65/-
(TIGR 268m00039) Tetrahymena
1.00/97
33348653 Trypanosoma brucei
(TIGR 8739m00013) T. cruzi
29245763 Giardia
0.1 substitutions/site

Figure S1.2: Mnd1 homologs, unrooted, with Fungi and Opisthokonts constrained. 202 aligned amino acid sites were analyzed, this consensus topology derived from 850 trees, $\alpha = 2.80$ ($2.18 < \alpha < 3.52$), pI = 0.01 (0.0005 < pI < 0.043) and lnL = –11589.94. (Same as **Figure 2B**) For the unconstrained analysis, see **Figure S1.19**.

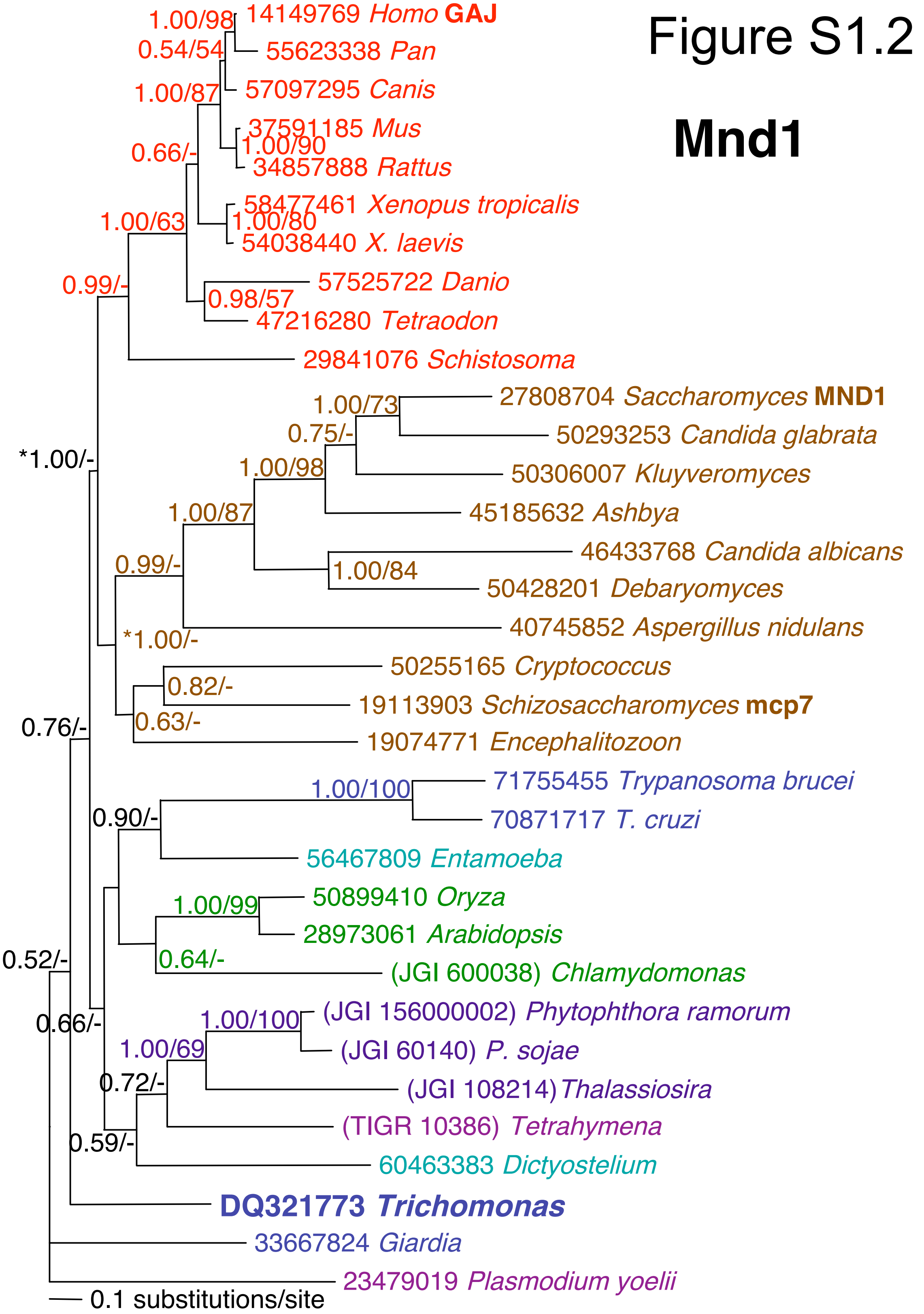

Figure S1.2
Mnd1
1.00/98
14149769 Homo GAJ
0.54/54
55623338 Pan
1.00/87
57097295 Canis
0.66/-
37591185 Mus
1.00/90
34857888 Rattus
58477461 Xenopus tropicalis
1.00/63
1.00/80
54038440 X. laevis
0.99/-
57525722 Danio
0.98/57
47216280 Tetraodon
29841076 Schistosoma
27808704 Saccharomyces MND1
1.00/73
0.75/-
50293253 Candida glabrata
*1.00/-
1.00/98
50306007 Kluyveromyces
1.00/87
45185632 Ashbya
46433768 Candida albicans
0.99/-
1.00/84
50428201 Debaryomyces
40745852 Aspergillus nidulans
*1.00/-
50255165 Cryptococcus
0.82/-
19113903 Schizosaccharomyces mcp7
0.76/-
0.63/-
19074771 Encephalitozoon
1.00/100
71755455 Trypanosoma brucei
0.90/-
70871717 T. cruzi
56467809 Entamoeba
50899410 Oryza
1.00/99
28973061 Arabidopsis
0.52/-
0.64/-
(JGI 600038) Chlamydomonas
0.66/-
1.00/100
(JGI 156000002) Phytophthora ramorum
1.00/69
(JGI 60140) P. sojae
0.72/-
(JGI 108214) Thalassiosira
(TIGR 10386) Tetrahymena
0.59/-
60463383 Dictyostelium
DQ321773 Trichomonas
33667824 Giardia
23479019 Plasmodium yoelii
0.1 substitutions/site

Figure S1.3: Spo11 homologs, rooted with the eukaryotic Top6A paralog outgroup, with Fungi and Opisthokonts constrained. 148 aligned amino acid sites were analyzed, this consensus topology derived from 700 trees, $\alpha = 1.76$ ($1.34 < \alpha < 2.23$), pI = 0.10 (0.03 < pI < 0.17) and lnL = –10624.08. (Same as **Figure 2C**) For the unconstrained analysis, see **Figure S1.6**.

Figure S1.3

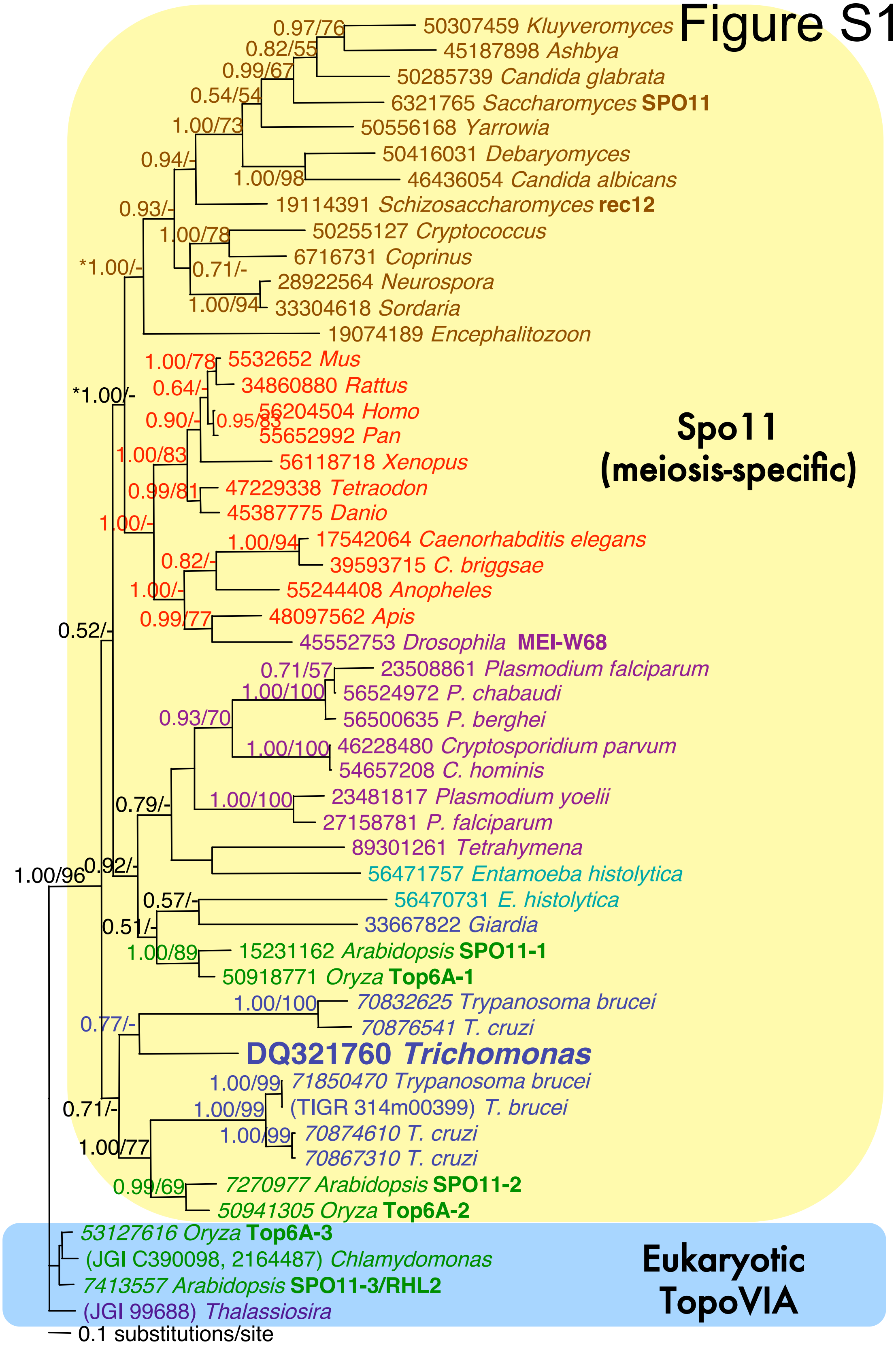

Spo11 (meiosis-specific)
Eukaryotic TopoVIA
50307459 Kluyveromyces
45187898 Ashbya
50285739 Candida glabrata
6321765 Saccharomyces SPO11
50556168 Yarrowia
50416031 Debaryomyces
46436054 Candida albicans
19114391 Schizosaccharomyces rec12
50255127 Cryptococcus
6716731 Coprinus
28922564 Neurospora
33304618 Sordaria
19074189 Encephalitozoon
5532652 Mus
34860880 Rattus
56204504 Homo
55652992 Pan
56118718 Xenopus
47229338 Tetraodon
45387775 Danio
17542064 Caenorhabditis elegans
39593715 C. briggsae
55244408 Anopheles
48097562 Apis
45552753 Drosophila MEI-W68
23508861 Plasmodium falciparum
56524972 P. chabaudi
56500635 P. berghei
46228480 Cryptosporidium parvum
54657208 C. hominis
23481817 Plasmodium yoelii
27158781 P. falciparum
89301261 Tetrahymena
56471757 Entamoeba histolytica
56470731 E. histolytica
33667822 Giardia
15231162 Arabidopsis SPO11-1
50918771 Oryza Top6A-1
70832625 Trypanosoma brucei
70876541 T. cruzi
DQ321760 Trichomonas
71850470 Trypanosoma brucei
(TIGR 314m00399) T. brucei
70874610 T. cruzi
70867310 T. cruzi
7270977 Arabidopsis SPO11-2
50941305 Oryza Top6A-2
53127616 Oryza Top6A-3
(JGI C390098, 2164487) Chlamydomonas
7413557 Arabidopsis SPO11-3/RHL2
(JGI 99688) Thalassiosira
0.97/76
0.82/55
0.99/67
0.54/54
1.00/73
0.94/-
1.00/98
0.93/-
1.00/78
0.71/-
*1.00/-
1.00/94
1.00/78
0.64/-
*1.00/-
0.90/-
0.95/83
1.00/83
0.99/81
1.00/-
1.00/94
0.82/-
1.00/-
0.99/77
0.52/-
0.71/57
1.00/100
0.93/70
1.00/100
0.79/-
1.00/100
0.92/-
1.00/96
0.57/-
0.51/-
1.00/89
1.00/100
0.77/-
1.00/99
0.71/-
1.00/99
1.00/99
1.00/77
0.99/69
0.1 substitutions/site

Figure S1.4: Mer3 homologs, unrooted. 610 aligned amino acid sites were analyzed, this consensus topology derived from 950 trees, $\alpha = 1.60$ ($1.39 < \alpha < 1.83$), pI = 0.04 (0.02 < pI < 0.06) and lnL = –27086.67. (Same as **Figure 2D**)

Figure S1.4

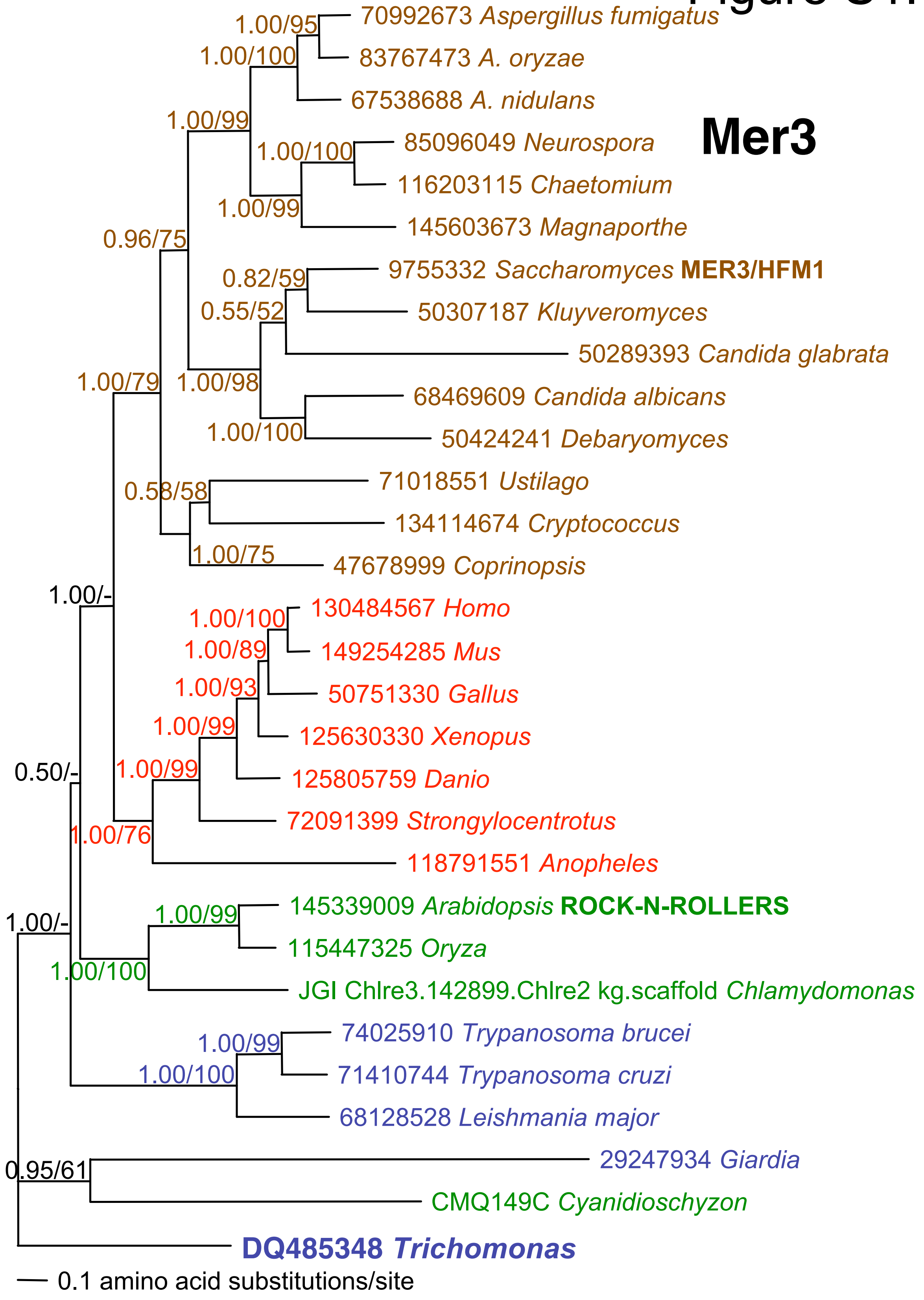
Mer3
1.00/95
70992673 Aspergillus fumigatus
1.00/100
83767473 A. oryzae
67538688 A. nidulans
1.00/99
85096049 Neurospora
1.00/100
116203115 Chaetomium
1.00/99
145603673 Magnaporthe
0.96/75
9755332 Saccharomyces MER3/HFM1
0.82/59
50307187 Kluyveromyces
0.55/52
50289393 Candida glabrata
1.00/79
1.00/98
68469609 Candida albicans
1.00/100
50424241 Debaryomyces
0.58/58
71018551 Ustilago
134114674 Cryptococcus
1.00/75
47678999 Coprinopsis
1.00/-
130484567 Homo
1.00/100
149254285 Mus
1.00/89
50751330 Gallus
1.00/93
125630330 Xenopus
1.00/99
125805759 Danio
0.50/-
1.00/99
72091399 Strongylocentrotus
1.00/76
118791551 Anopheles
145339009 Arabidopsis ROCK-N-ROLLERS
1.00/99
1.00/-
115447325 Oryza
1.00/100
JGI Chlre3.142899.Chlre2 kg.scaffold Chlamydomonas
74025910 Trypanosoma brucei
1.00/99
71410744 Trypanosoma cruzi
1.00/100
68128528 Leishmania major
29247934 Giardia
0.95/61
CMQ149C Cyanidioschyzon
DQ485348 Trichomonas
0.1 amino acid substitutions/site

Figure S1.5: Spo11 homologs, rooted with the prokaryotic Top6A ortholog outgroup. 134 aligned amino acid sites were analyzed, this consensus topology derived from 970 trees, $\alpha = 1.63$ ($1.18 < \alpha < 2.10$), $pI = 0.10$ ($0.001 < pI < 0.17$) and $\ln L = -10611.60$.

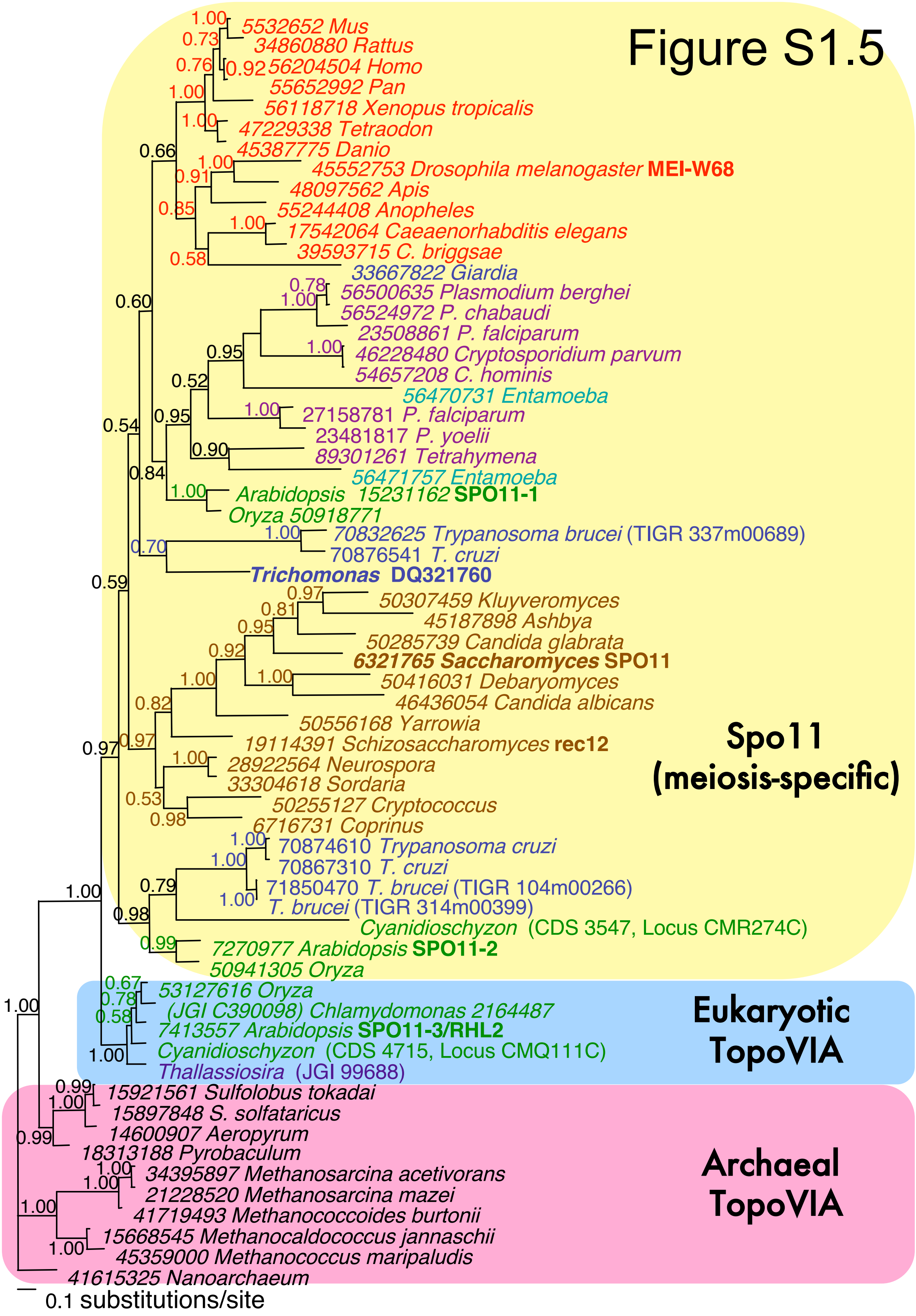

Figure S1.5
5532652 Mus
34860880 Rattus
56204504 Homo
55652992 Pan
56118718 Xenopus tropicalis
47229338 Tetraodon
45387775 Danio
45552753 Drosophila melanogaster MEI-W68
48097562 Apis
55244408 Anopheles
17542064 Caeaenorhabditis elegans
39593715 C. briggsae
33667822 Giardia
56500635 Plasmodium berghei
56524972 P. chabaudi
23508861 P. falciparum
46228480 Cryptosporidium parvum
54657208 C. hominis
56470731 Entamoeba
27158781 P. falciparum
23481817 P. yoelii
89301261 Tetrahymena
56471757 Entamoeba
Arabidopsis 15231162 SPO11-1
Oryza 50918771
70832625 Trypanosoma brucei (TIGR 337m00689)
70876541 T. cruzi
Trichomonas DQ321760
50307459 Kluyveromyces
45187898 Ashbya
50285739 Candida glabrata
6321765 Saccharomyces SPO11
50416031 Debaryomyces
46436054 Candida albicans
50556168 Yarrowia
19114391 Schizosaccharomyces rec12
28922564 Neurospora
33304618 Sordaria
50255127 Cryptococcus
6716731 Coprinus
70874610 Trypanosoma cruzi
70867310 T. cruzi
71850470 T. brucei (TIGR 104m00266)
T. brucei (TIGR 314m00399)
Cyanidioschyzon (CDS 3547, Locus CMR274C)
7270977 Arabidopsis SPO11-2
50941305 Oryza
Spo11
(meiosis-specific)
53127616 Oryza
(JGI C390098) Chlamydomonas 2164487
7413557 Arabidopsis SPO11-3/RHL2
Cyanidioschyzon (CDS 4715, Locus CMQ111C)
Thallassiosira (JGI 99688)
Eukaryotic
TopoVIA
15921561 Sulfolobus tokadai
15897848 S. solfataricus
14600907 Aeropyrum
18313188 Pyrobaculum
34395897 Methanosarcina acetivorans
21228520 Methanosarcina mazei
41719493 Methanococcoides burtonii
15668545 Methanocaldococcus jannaschii
45359000 Methanococcus maripaludis
41615325 Nanoarchaeum
Archaeal
TopoVIA
0.1 substitutions/site

Figure S1.6: Spo11 homologs, rooted with the eukaryotic Top6A paralog outgroup. 148 aligned amino acid sites were analyzed, this consensus topology derived from 925 trees, $\alpha = 1.69$ ($1.23 < \alpha < 2.19$), $pI = 0.11$ ($0.04 < pI < 0.19$) and $\ln L = -10617.54$.

Figure S1.6

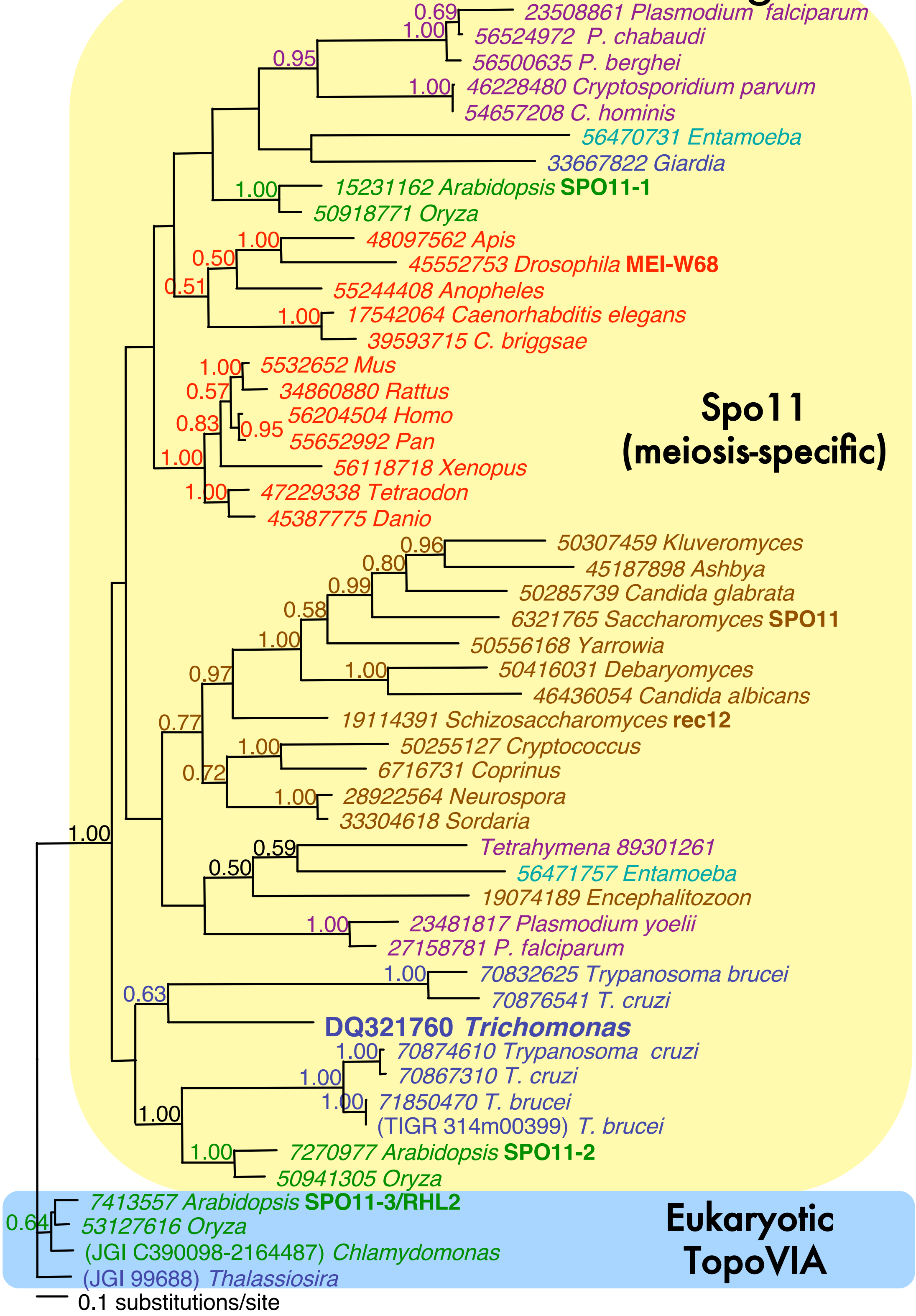
23508861 Plasmodium falciparum
56524972 P. chabaudi
56500635 P. berghei
46228480 Cryptosporidium parvum
54657208 C. hominis
56470731 Entamoeba
33667822 Giardia
15231162 Arabidopsis SPO11-1
50918771 Oryza
48097562 Apis
45552753 Drosophila MEI-W68
55244408 Anopheles
17542064 Caenorhabditis elegans
39593715 C. briggsae
5532652 Mus
34860880 Rattus
56204504 Homo
55652992 Pan
56118718 Xenopus
47229338 Tetraodon
45387775 Danio
50307459 Kluveromyces
45187898 Ashbya
50285739 Candida glabrata
6321765 Saccharomyces SPO11
50556168 Yarrowia
50416031 Debaryomyces
46436054 Candida albicans
19114391 Schizosaccharomyces rec12
50255127 Cryptococcus
6716731 Coprinus
28922564 Neurospora
33304618 Sordaria
Tetrahymena 89301261
56471757 Entamoeba
19074189 Encephalitozoon
23481817 Plasmodium yoelii
27158781 P. falciparum
70832625 Trypanosoma brucei
70876541 T. cruzi
DQ321760 Trichomonas
70874610 Trypanosoma cruzi
70867310 T. cruzi
71850470 T. brucei
(TIGR 314m00399) T. brucei
7270977 Arabidopsis SPO11-2
50941305 Oryza
7413557 Arabidopsis SPO11-3/RHL2
53127616 Oryza
(JGI C390098-2164487) Chlamydomonas
(JGI 99688) Thalassiosira
Spo11
(meiosis-specific)
Eukaryotic
TopoVIA
0.1 substitutions/site

Figure S1.7: Spo11 homologs, unrooted. 138 aligned amino acid sites were analyzed, this consensus topology derived from 980 trees, $\alpha = 1.40$ ($1.00 < \alpha < 1.85$), $pI = 0.08$ ($0.01 < pI < 0.16$) and $\ln L = -9617.07$.

Figure S1.7

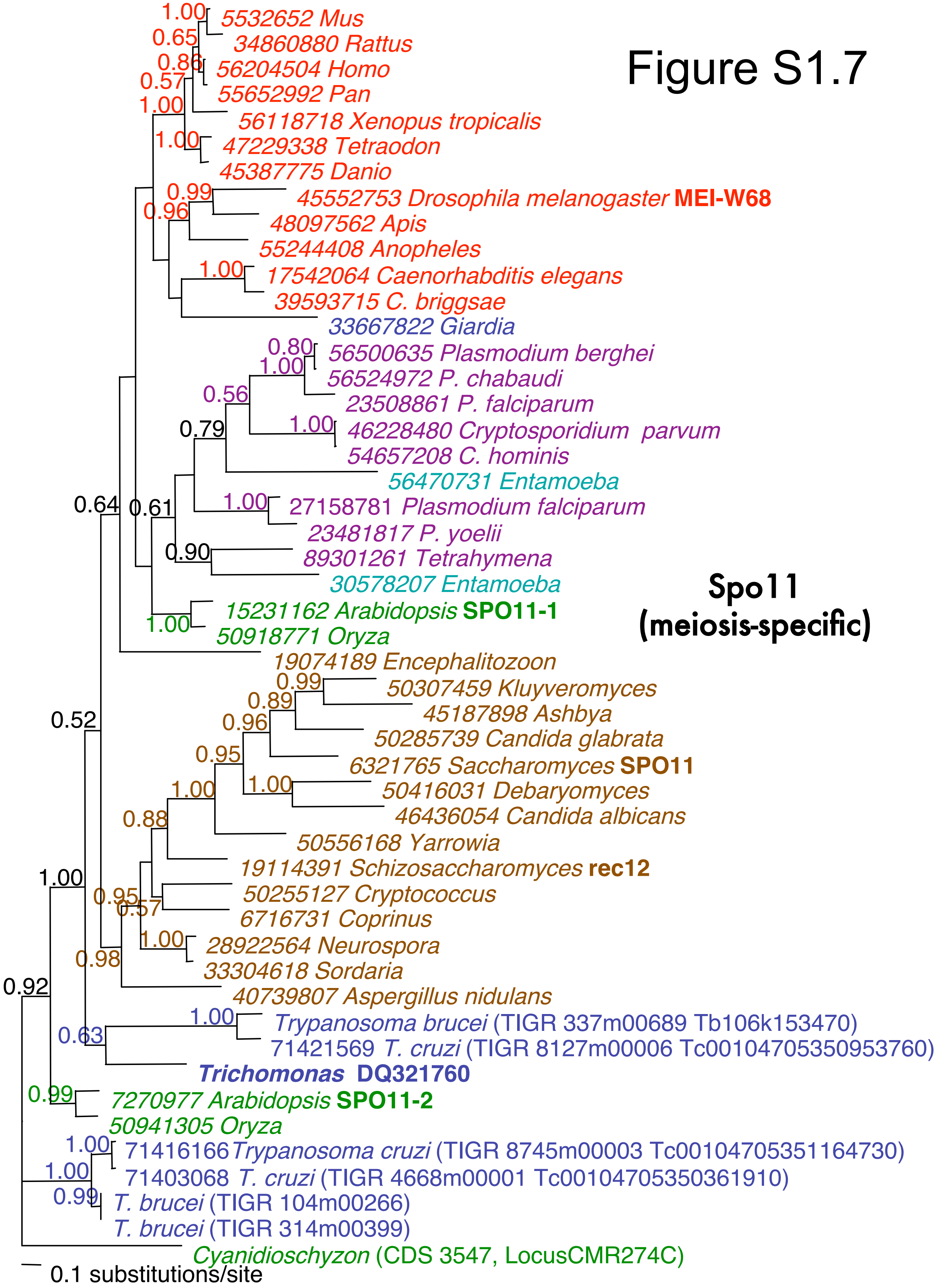

5532652 Mus
34860880 Rattus
56204504 Homo
55652992 Pan
56118718 Xenopus tropicalis
47229338 Tetraodon
45387775 Danio
45552753 Drosophila melanogaster MEI-W68
48097562 Apis
55244408 Anopheles
17542064 Caenorhabditis elegans
39593715 C. briggsae
33667822 Giardia
56500635 Plasmodium berghei
56524972 P. chabaudi
23508861 P. falciparum
46228480 Cryptosporidium parvum
54657208 C. hominis
56470731 Entamoeba
27158781 Plasmodium falciparum
23481817 P. yoelii
89301261 Tetrahymena
30578207 Entamoeba
15231162 Arabidopsis SPO11-1
50918771 Oryza
Spo11
(meiosis-specific)
19074189 Encephalitozoon
50307459 Kluyveromyces
45187898 Ashbya
50285739 Candida glabrata
6321765 Saccharomyces SPO11
50416031 Debaryomyces
46436054 Candida albicans
50556168 Yarrowia
19114391 Schizosaccharomyces rec12
50255127 Cryptococcus
6716731 Coprinus
28922564 Neurospora
33304618 Sordaria
40739807 Aspergillus nidulans
Trypanosoma brucei (TIGR 337m00689 Tb106k153470)
71421569 T. cruzi (TIGR 8127m00006 Tc00104705350953760)
Trichomonas DQ321760
7270977 Arabidopsis SPO11-2
50941305 Oryza
71416166 Trypanosoma cruzi (TIGR 8745m00003 Tc00104705351164730)
71403068 T. cruzi (TIGR 4668m00001 Tc00104705350361910)
T. brucei (TIGR 104m00266)
T. brucei (TIGR 314m00399)
Cyanidioschyzon (CDS 3547, LocusCMR274C)
0.1 substitutions/site
1.00
0.65
0.86
0.57
1.00
1.00
0.99
0.96
1.00
0.80
1.00
0.56
0.79
1.00
0.64
0.61
1.00
0.90
1.00
0.99
0.89
0.96
0.52
0.95
1.00
1.00
0.88
1.00
0.95
0.57
1.00
0.98
0.92
1.00
0.63
0.99
1.00
1.00
0.99

Figure S1.8: Mre11 homologs, rooted with the archaeal SbcD homolog outgroup. 253 aligned amino acid sites were analyzed, this consensus topology derived from 850 trees, $\alpha = 1.68$ ($1.36 < \alpha < 2.05$), pI = 0.05 (0.02 < pI < 0.09) and lnL = –17300.85.

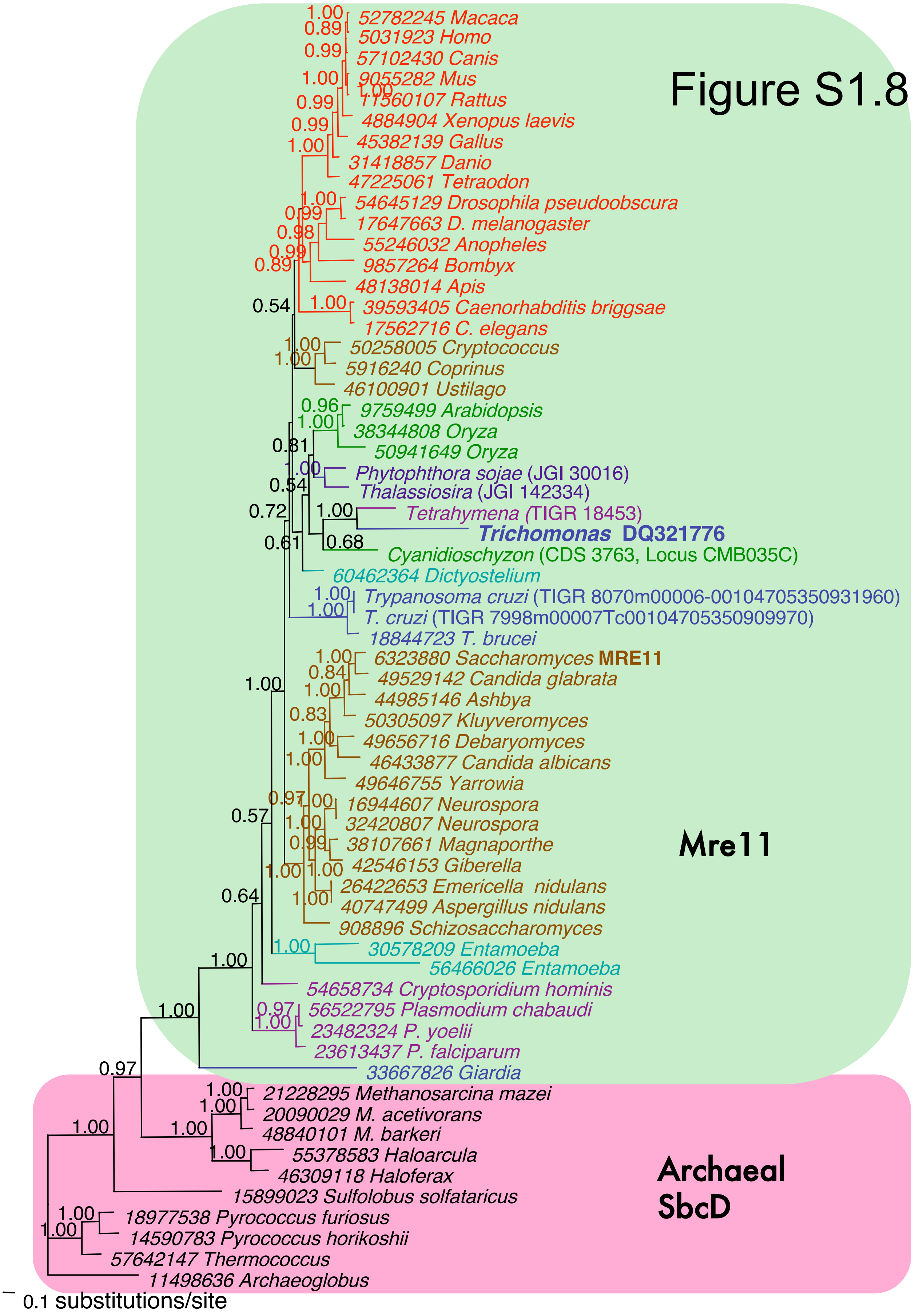
Figure S1.8
52782245 Macaca
5031923 Homo
57102430 Canis
9055282 Mus
11560107 Rattus
4884904 Xenopus laevis
45382139 Gallus
31418857 Danio
47225061 Tetraodon
54645129 Drosophila pseudoobscura
17647663 D. melanogaster
55246032 Anopheles
9857264 Bombyx
48138014 Apis
39593405 Caenorhabditis briggsae
17562716 C. elegans
50258005 Cryptococcus
5916240 Coprinus
46100901 Ustilago
9759499 Arabidopsis
38344808 Oryza
50941649 Oryza
Phytophthora sojae (JGI 30016)
Thalassiosira (JGI 142334)
Tetrahymena (TIGR 18453)
Trichomonas DQ321776
Cyanidioschyzon (CDS 3763, Locus CMB035C)
60462364 Dictyostelium
Trypanosoma cruzi (TIGR 8070m00006-00104705350931960)
T. cruzi (TIGR 7998m00007Tc00104705350909970)
18844723 T. brucei
6323880 Saccharomyces MRE11
49529142 Candida glabrata
44985146 Ashbya
50305097 Kluyveromyces
49656716 Debaryomyces
46433877 Candida albicans
49646755 Yarrowia
16944607 Neurospora
32420807 Neurospora
38107661 Magnaporthe
42546153 Giberella
26422653 Emericella nidulans
40747499 Aspergillus nidulans
908896 Schizosaccharomyces
30578209 Entamoeba
56466026 Entamoeba
54658734 Cryptosporidium hominis
56522795 Plasmodium chabaudi
23482324 P. yoelii
23613437 P. falciparum
33667826 Giardia
Mre11
21228295 Methanosarcina mazei
20090029 M. acetivorans
48840101 M. barkeri
55378583 Haloarcula
46309118 Haloferax
15899023 Sulfolobus solfataricus
18977538 Pyrococcus furiosus
14590783 Pyrococcus horikoshii
57642147 Thermococcus
11498636 Archaeoglobus
Archaeal
SbcD
0.1 substitutions/site

Figure S1.9: Mre11 homologs, unrooted. 342 aligned amino acid sites were analyzed, this consensus topology derived from 960 trees, $\alpha = 1.57$ ($1.30 < \alpha < 1.86$), pI = 0.027 (0.005 < pI < 0.055) and lnL = –19374.82.

Figure S1.9

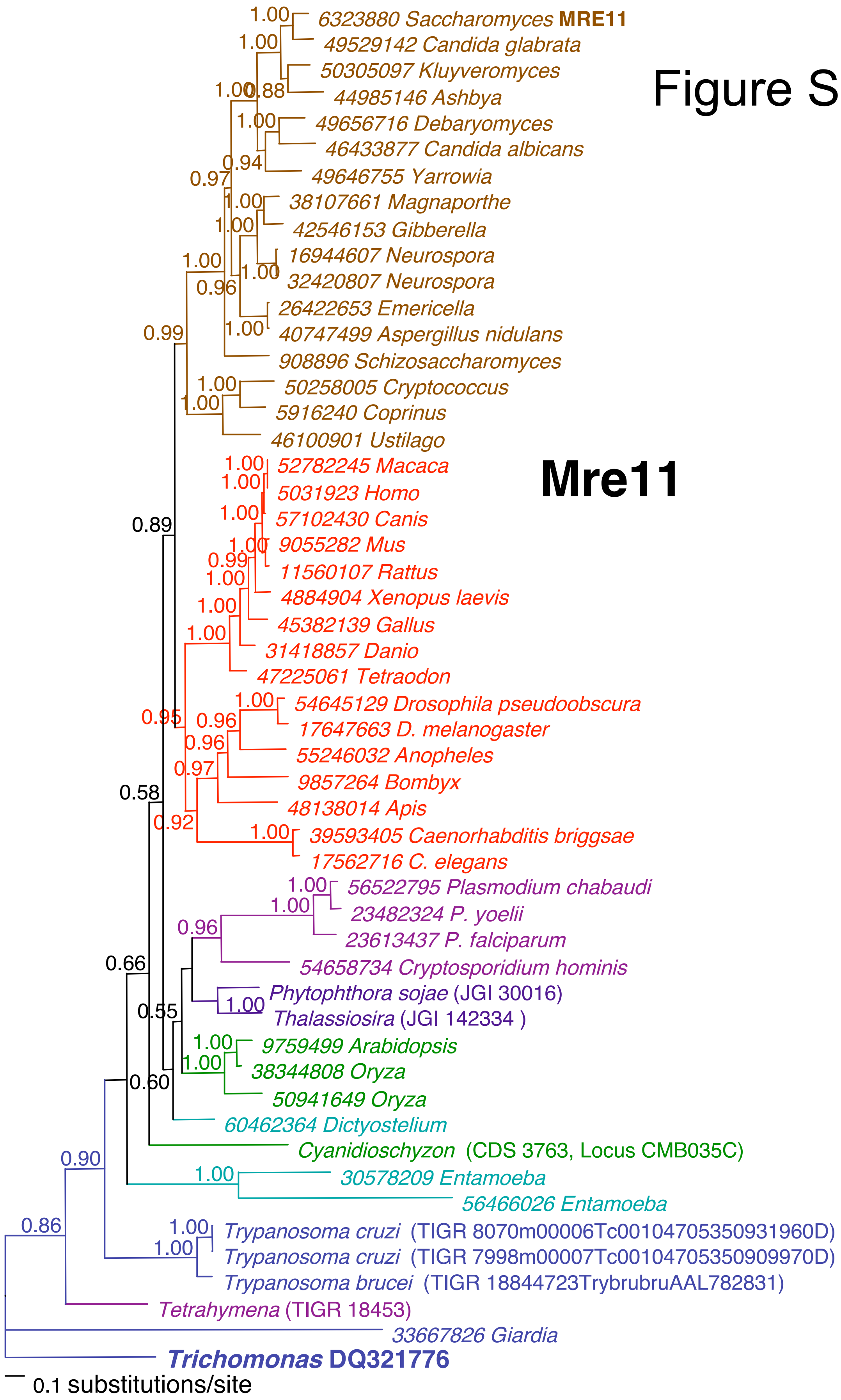
Mre11
6323880 Saccharomyces MRE11
49529142 Candida glabrata
50305097 Kluyveromyces
44985146 Ashbya
49656716 Debaryomyces
46433877 Candida albicans
49646755 Yarrowia
38107661 Magnaporthe
42546153 Gibberella
16944607 Neurospora
32420807 Neurospora
26422653 Emericella
40747499 Aspergillus nidulans
908896 Schizosaccharomyces
50258005 Cryptococcus
5916240 Coprinus
46100901 Ustilago
52782245 Macaca
5031923 Homo
57102430 Canis
9055282 Mus
11560107 Rattus
4884904 Xenopus laevis
45382139 Gallus
31418857 Danio
47225061 Tetraodon
54645129 Drosophila pseudoobscura
17647663 D. melanogaster
55246032 Anopheles
9857264 Bombyx
48138014 Apis
39593405 Caenorhabditis briggsae
17562716 C. elegans
56522795 Plasmodium chabaudi
23482324 P. yoelii
23613437 P. falciparum
54658734 Cryptosporidium hominis
Phytophthora sojae (JGI 30016)
Thalassiosira (JGI 142334)
9759499 Arabidopsis
38344808 Oryza
50941649 Oryza
60462364 Dictyostelium
Cyanidioschyzon (CDS 3763, Locus CMB035C)
30578209 Entamoeba
56466026 Entamoeba
Trypanosoma cruzi (TIGR 8070m00006Tc00104705350931960D)
Trypanosoma cruzi (TIGR 7998m00007Tc00104705350909970D)
Trypanosoma brucei (TIGR 18844723TrybrubruAAL782831)
Tetrahymena (TIGR 18453)
33667826 Giardia
Trichomonas DQ321776
0.1 substitutions/site

Figure S1.10: Rad50 homologs, rooted with the archaeal SbcC homolog outgroup. 413 aligned amino acid sites were analyzed, this consensus topology derived from 970 trees, $\alpha = 1.73$ ($1.48 < \alpha < 1.97$), pI = 0.029 (0.010 < pI < 0.056) and lnL = –39542.71.

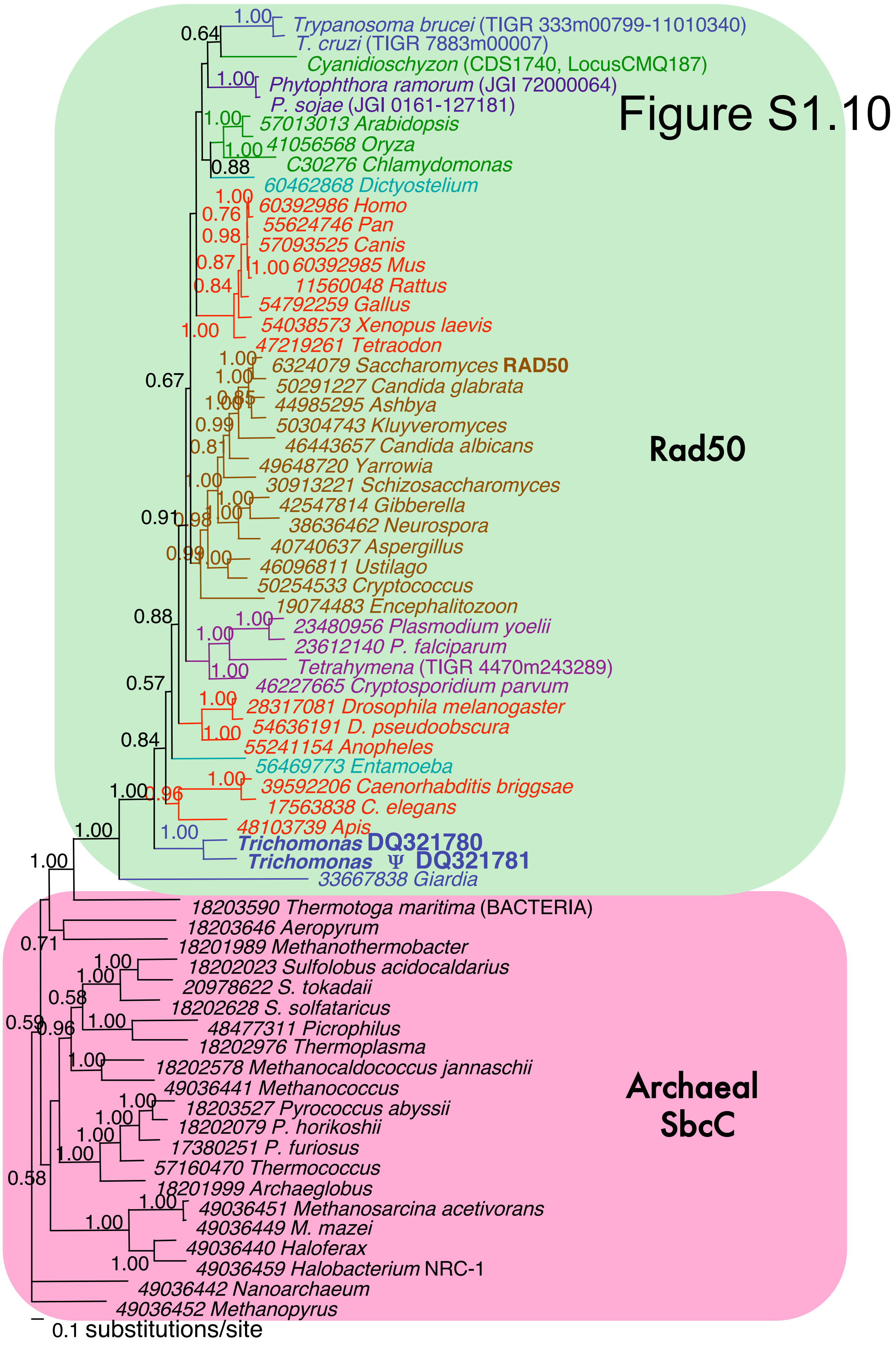

Figure S1.10
Rad50
Archaeal SbcC
Trypanosoma brucei (TIGR 333m00799-11010340)
T. cruzi (TIGR 7883m00007)
Cyanidioschyzon (CDS1740, LocusCMQ187)
Phytophthora ramorum (JGI 72000064)
P. sojae (JGI 0161-127181)
57013013 Arabidopsis
41056568 Oryza
C30276 Chlamydomonas
60462868 Dictyostelium
60392986 Homo
55624746 Pan
57093525 Canis
60392985 Mus
11560048 Rattus
54792259 Gallus
54038573 Xenopus laevis
47219261 Tetraodon
6324079 Saccharomyces RAD50
50291227 Candida glabrata
44985295 Ashbya
50304743 Kluyveromyces
46443657 Candida albicans
49648720 Yarrowia
30913221 Schizosaccharomyces
42547814 Gibberella
38636462 Neurospora
40740637 Aspergillus
46096811 Ustilago
50254533 Cryptococcus
19074483 Encephalitozoon
23480956 Plasmodium yoelii
23612140 P. falciparum
Tetrahymena (TIGR 4470m243289)
46227665 Cryptosporidium parvum
28317081 Drosophila melanogaster
54636191 D. pseudoobscura
55241154 Anopheles
56469773 Entamoeba
39592206 Caenorhabditis briggsae
17563838 C. elegans
48103739 Apis
Trichomonas DQ321780
Trichomonas Ψ DQ321781
33667838 Giardia
18203590 Thermotoga maritima (BACTERIA)
18203646 Aeropyrum
18201989 Methanothermobacter
18202023 Sulfolobus acidocaldarius
20978622 S. tokadaii
18202628 S. solfataricus
48477311 Picrophilus
18202976 Thermoplasma
18202578 Methanocaldococcus jannaschii
49036441 Methanococcus
18203527 Pyrococcus abyssii
18202079 P. horikoshii
17380251 P. furiosus
57160470 Thermococcus
18201999 Archaeglobus
49036451 Methanosarcina acetivorans
49036449 M. mazei
49036440 Haloferax
49036459 Halobacterium NRC-1
49036442 Nanoarchaeum
49036452 Methanopyrus
0.1 substitutions/site

Figure S1.11: Rad50 homologs, unrooted. 600 aligned amino acid sites were analyzed, this consensus topology derived from 980 trees, $\alpha = 1.61$ ($1.41 < \alpha < 1.83$), pI = 0.033 (0.015 < pI < 0.054) and lnL = –35954.03.

Figure S1.11

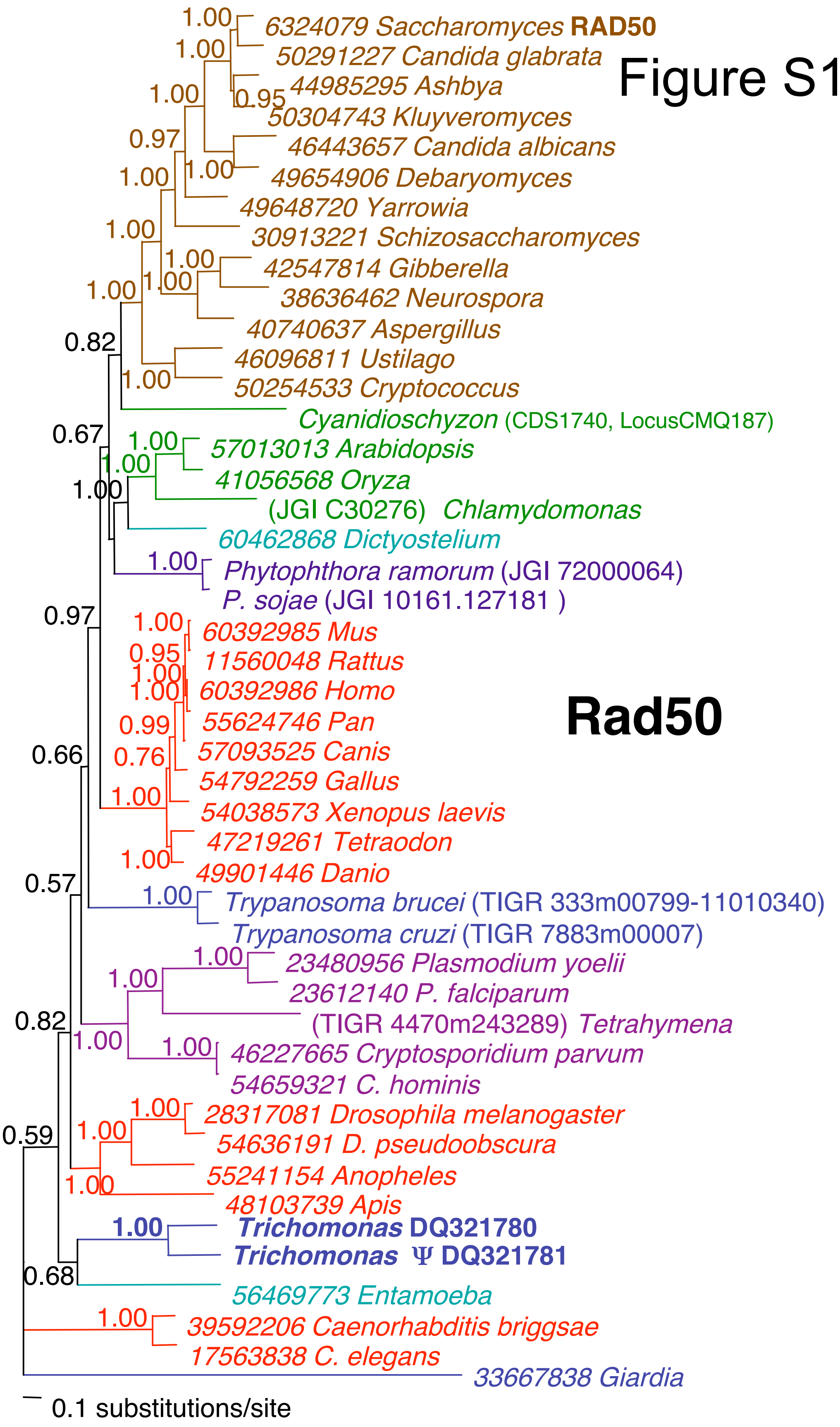

Rad50
1.00
6324079 Saccharomyces RAD50
1.00
50291227 Candida glabrata
1.00
0.95
44985295 Ashbya
50304743 Kluyveromyces
0.97
46443657 Candida albicans
1.00
1.00
49654906 Debaryomyces
49648720 Yarrowia
1.00
30913221 Schizosaccharomyces
1.00
42547814 Gibberella
1.00
1.00
38636462 Neurospora
40740637 Aspergillus
0.82
46096811 Ustilago
1.00
50254533 Cryptococcus
Cyanidioschyzon (CDS1740, LocusCMQ187)
0.67
1.00
57013013 Arabidopsis
1.00
41056568 Oryza
1.00
(JGI C30276) Chlamydomonas
60462868 Dictyostelium
1.00
Phytophthora ramorum (JGI 72000064)
P. sojae (JGI 10161.127181)
0.97
1.00
60392985 Mus
0.95
11560048 Rattus
1.00
1.00
60392986 Homo
0.99
55624746 Pan
0.76
57093525 Canis
0.66
54792259 Gallus
1.00
54038573 Xenopus laevis
47219261 Tetraodon
1.00
49901446 Danio
0.57
1.00
Trypanosoma brucei (TIGR 333m00799-11010340)
Trypanosoma cruzi (TIGR 7883m00007)
1.00
23480956 Plasmodium yoelii
1.00
23612140 P. falciparum
(TIGR 4470m243289) Tetrahymena
0.82
1.00
1.00
46227665 Cryptosporidium parvum
54659321 C. hominis
1.00
28317081 Drosophila melanogaster
1.00
54636191 D. pseudoobscura
0.59
55241154 Anopheles
1.00
48103739 Apis
1.00
Trichomonas DQ321780
Trichomonas Ψ DQ321781
0.68
56469773 Entamoeba
1.00
39592206 Caenorhabditis briggsae
17563838 C. elegans
33667838 Giardia
0.1 substitutions/site

Figure S1.12: Rad1 (MEI-9/ERCC4) homologs, rooted with the archaeal ERCC4 homolog outgroup. 469 aligned amino acid sites were analyzed, this consensus topology derived from 980 trees, $\alpha = 2.59$ ($2.23 < \alpha < 2.99$), pI = 0.0032 (0.0001 < pI < 0.012) and lnL = –31615.54.

Figure S1.12

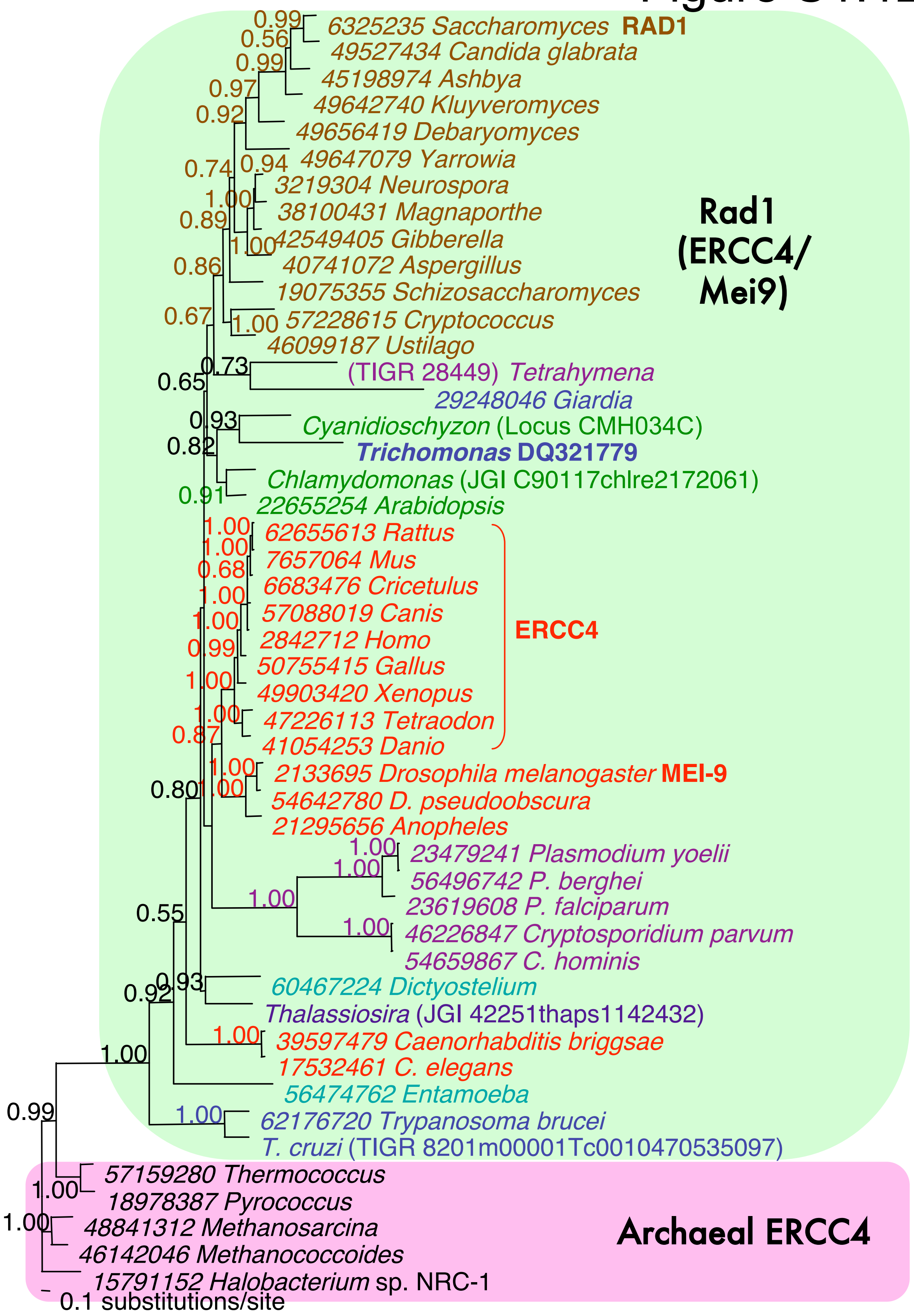

6325235 Saccharomyces RAD1
49527434 Candida glabrata
45198974 Ashbya
49642740 Kluyveromyces
49656419 Debaryomyces
49647079 Yarrowia
3219304 Neurospora
38100431 Magnaporthe
42549405 Gibberella
40741072 Aspergillus
19075355 Schizosaccharomyces
57228615 Cryptococcus
46099187 Ustilago
(TIGR 28449) Tetrahymena
29248046 Giardia
Cyanidioschyzon (Locus CMH034C)
Trichomonas DQ321779
Chlamydomonas (JGI C90117chlre2172061)
22655254 Arabidopsis
62655613 Rattus
7657064 Mus
6683476 Cricetulus
57088019 Canis
2842712 Homo
50755415 Gallus
49903420 Xenopus
47226113 Tetraodon
41054253 Danio
ERCC4
2133695 Drosophila melanogaster MEI-9
54642780 D. pseudoobscura
21295656 Anopheles
23479241 Plasmodium yoelii
56496742 P. berghei
23619608 P. falciparum
46226847 Cryptosporidium parvum
54659867 C. hominis
60467224 Dictyostelium
Thalassiosira (JGI 42251thaps1142432)
39597479 Caenorhabditis briggsae
17532461 C. elegans
56474762 Entamoeba
62176720 Trypanosoma brucei
T. cruzi (TIGR 8201m00001Tc0010470535097)
Rad1 (ERCC4/ Mei9)
57159280 Thermococcus
18978387 Pyrococcus
48841312 Methanosarcina
46142046 Methanococcoides
15791152 Halobacterium sp. NRC-1
Archaeal ERCC4
0.1 substitutions/site

Figure S1.13: Rad1 (MEI-9/ERCC4) homologs, unrooted. 535 aligned amino acid sites were analyzed, this consensus topology derived from 950 trees, $\alpha = 2.28$ ($1.99 < \alpha < 2.61$), $pI = 0.0028$ ($0.0001 < pI < 0.011$) and $\ln L = -32135.51$.

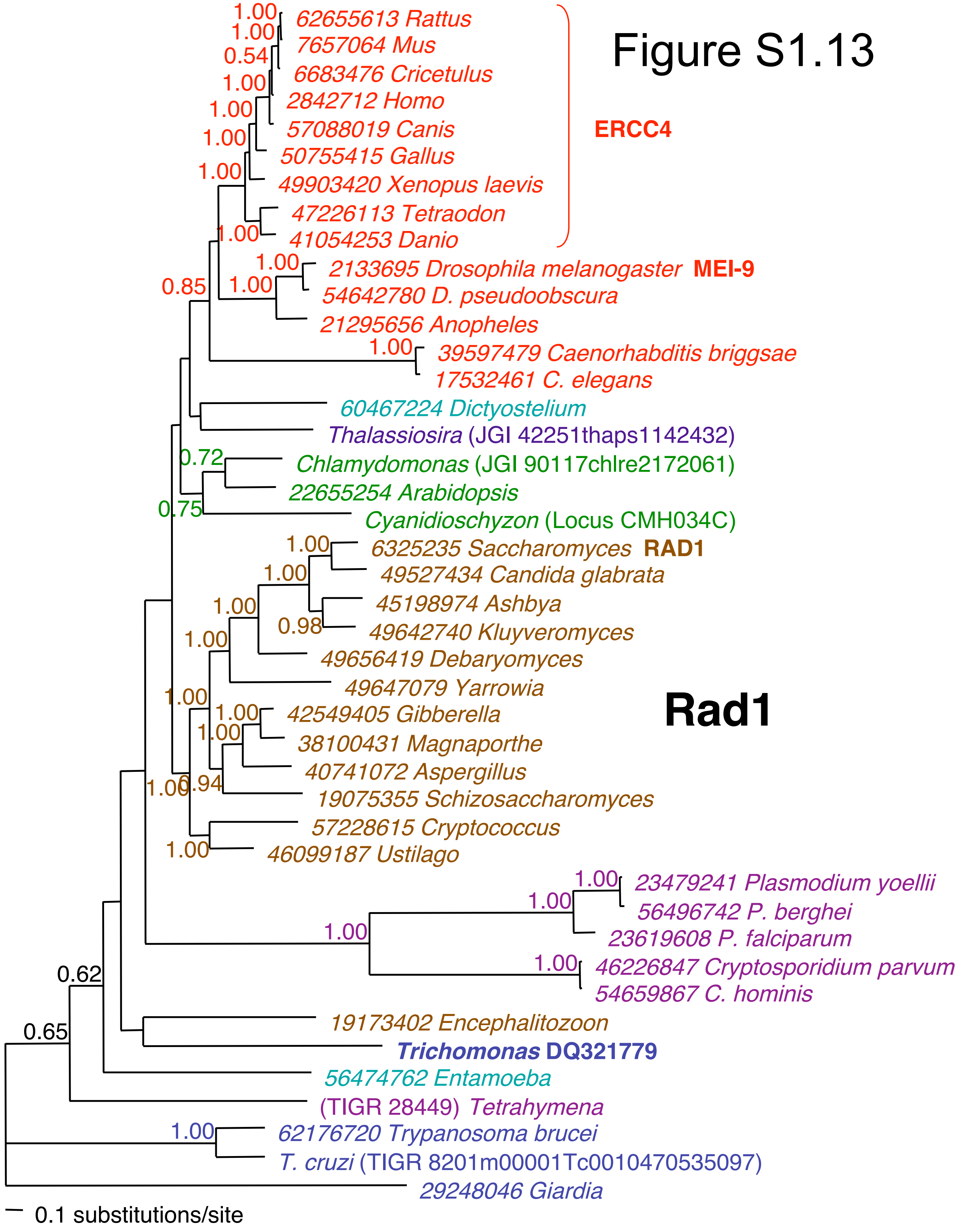
Figure S1.13
62655613 Rattus
7657064 Mus
6683476 Cricetulus
2842712 Homo
57088019 Canis
50755415 Gallus
49903420 Xenopus laevis
47226113 Tetraodon
41054253 Danio
ERCC4
2133695 Drosophila melanogaster MEI-9
54642780 D. pseudoobscura
21295656 Anopheles
39597479 Caenorhabditis briggsae
17532461 C. elegans
60467224 Dictyostelium
Thalassiosira (JGI 42251thaps1142432)
Chlamydomonas (JGI 90117chlre2172061)
22655254 Arabidopsis
Cyanidioschyzon (Locus CMH034C)
6325235 Saccharomyces RAD1
49527434 Candida glabrata
45198974 Ashbya
49642740 Kluyveromyces
49656419 Debaryomyces
49647079 Yarrowia
42549405 Gibberella
38100431 Magnaporthe
40741072 Aspergillus
19075355 Schizosaccharomyces
57228615 Cryptococcus
46099187 Ustilago
Rad1
23479241 Plasmodium yoellii
56496742 P. berghei
23619608 P. falciparum
46226847 Cryptosporidium parvum
54659867 C. hominis
19173402 Encephalitozoon
Trichomonas DQ321779
56474762 Entamoeba
(TIGR 28449) Tetrahymena
62176720 Trypanosoma brucei
T. cruzi (TIGR 8201m00001Tc0010470535097)
29248046 Giardia
0.1 substitutions/site

Figure S1.14: Hop1, rooted with paralogs Rev7 and Mad2, altogether known as HORMA domain proteins. 155 aligned amino acid sites were analyzed, this consensus topology derived from 850 trees, $\alpha = 3.63$ ($2.62 < \alpha < 4.83$), pI = 0.28 (0.17 < pI < 0.39) and lnL = –22876.07. Since REV7 and MAD2 homologs were not found in *T. vaginalis,* only homologs found by PSI-BLAST of the NCBI non-redundant protein database were included, and other protist genome databases were not searched comprehensively for REV7 and MAD2.

Figure S1.14

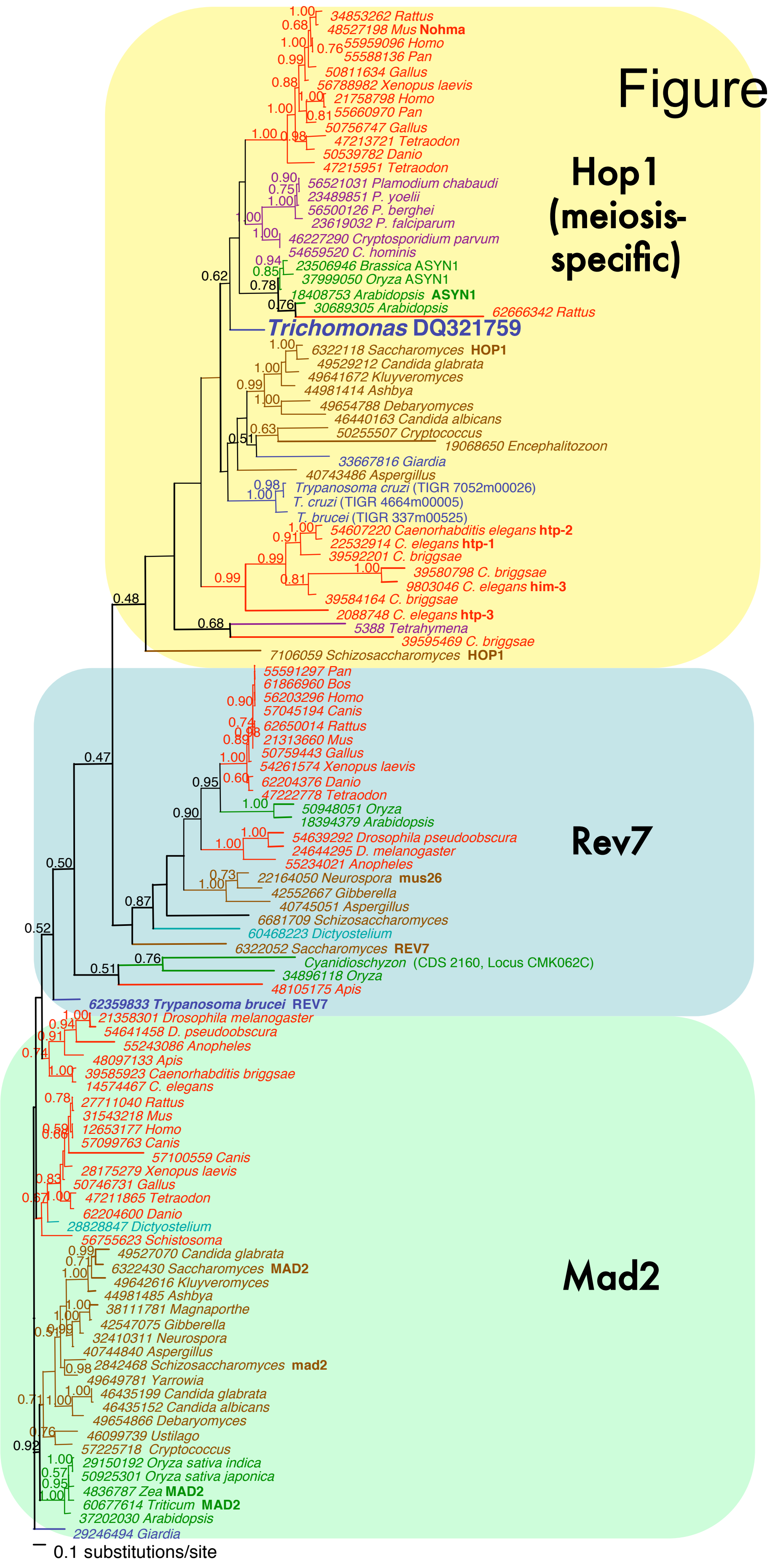

Hop1 (meiosis-specific)
34853262 Rattus
48527198 Mus Nohma
55959096 Homo
55588136 Pan
50811634 Gallus
56788982 Xenopus laevis
21758798 Homo
55660970 Pan
50756747 Gallus
47213721 Tetraodon
50539782 Danio
47215951 Tetraodon
56521031 Plamodium chabaudi
23489851 P. yoelii
56500126 P. berghei
23619032 P. falciparum
46227290 Cryptosporidium parvum
54659520 C. hominis
23506946 Brassica ASYN1
37999050 Oryza ASYN1
18408753 Arabidopsis ASYN1
30689305 Arabidopsis
62666342 Rattus
Trichomonas DQ321759
6322118 Saccharomyces HOP1
49529212 Candida glabrata
49641672 Kluyveromyces
44981414 Ashbya
49654788 Debaryomyces
46440163 Candida albicans
50255507 Cryptococcus
19068650 Encephalitozoon
33667816 Giardia
40743486 Aspergillus
Trypanosoma cruzi (TIGR 7052m00026)
T. cruzi (TIGR 4664m00005)
T. brucei (TIGR 337m00525)
54607220 Caenorhabditis elegans htp-2
22532914 C. elegans htp-1
39592201 C. briggsae
39580798 C. briggsae
9803046 C. elegans him-3
39584164 C. briggsae
2088748 C. elegans htp-3
5388 Tetrahymena
39595469 C. briggsae
7106059 Schizosaccharomyces HOP1
Rev7
55591297 Pan
61866960 Bos
56203296 Homo
57045194 Canis
62650014 Rattus
21313660 Mus
50759443 Gallus
54261574 Xenopus laevis
62204376 Danio
47222778 Tetraodon
50948051 Oryza
18394379 Arabidopsis
54639292 Drosophila pseudoobscura
24644295 D. melanogaster
55234021 Anopheles
22164050 Neurospora mus26
42552667 Gibberella
40745051 Aspergillus
6681709 Schizosaccharomyces
60468223 Dictyostelium
6322052 Saccharomyces REV7
Cyanidioschyzon (CDS 2160, Locus CMK062C)
34896118 Oryza
48105175 Apis
62359833 Trypanosoma brucei REV7
Mad2
21358301 Drosophila melanogaster
54641458 D. pseudoobscura
55243086 Anopheles
48097133 Apis
39585923 Caenorhabditis briggsae
14574467 C. elegans
27711040 Rattus
31543218 Mus
12653177 Homo
57099763 Canis
57100559 Canis
28175279 Xenopus laevis
50746731 Gallus
47211865 Tetraodon
62204600 Danio
28828847 Dictyostelium
56755623 Schistosoma
49527070 Candida glabrata
6322430 Saccharomyces MAD2
49642616 Kluyveromyces
44981485 Ashbya
38111781 Magnaporthe
42547075 Gibberella
32410311 Neurospora
40744840 Aspergillus
2842468 Schizosaccharomyces mad2
49649781 Yarrowia
46435199 Candida glabrata
46435152 Candida albicans
49654866 Debaryomyces
46099739 Ustilago
57225718 Cryptococcus
29150192 Oryza sativa indica
50925301 Oryza sativa japonica
4836787 Zea MAD2
60677614 Triticum MAD2
37202030 Arabidopsis
29246494 Giardia
0.1 substitutions/site

Figure S1.15: Hop1 homologs, rooted with paralog Rev7, with Fungi constrained. 115 aligned amino acid sites were analyzed, this consensus topology derived from 990 trees, $\alpha = 4.31$ ($2.86 < \alpha < 6.43$), pI = 0.034 (0.0008 < pI < 0.11) and lnL = –8966.90.

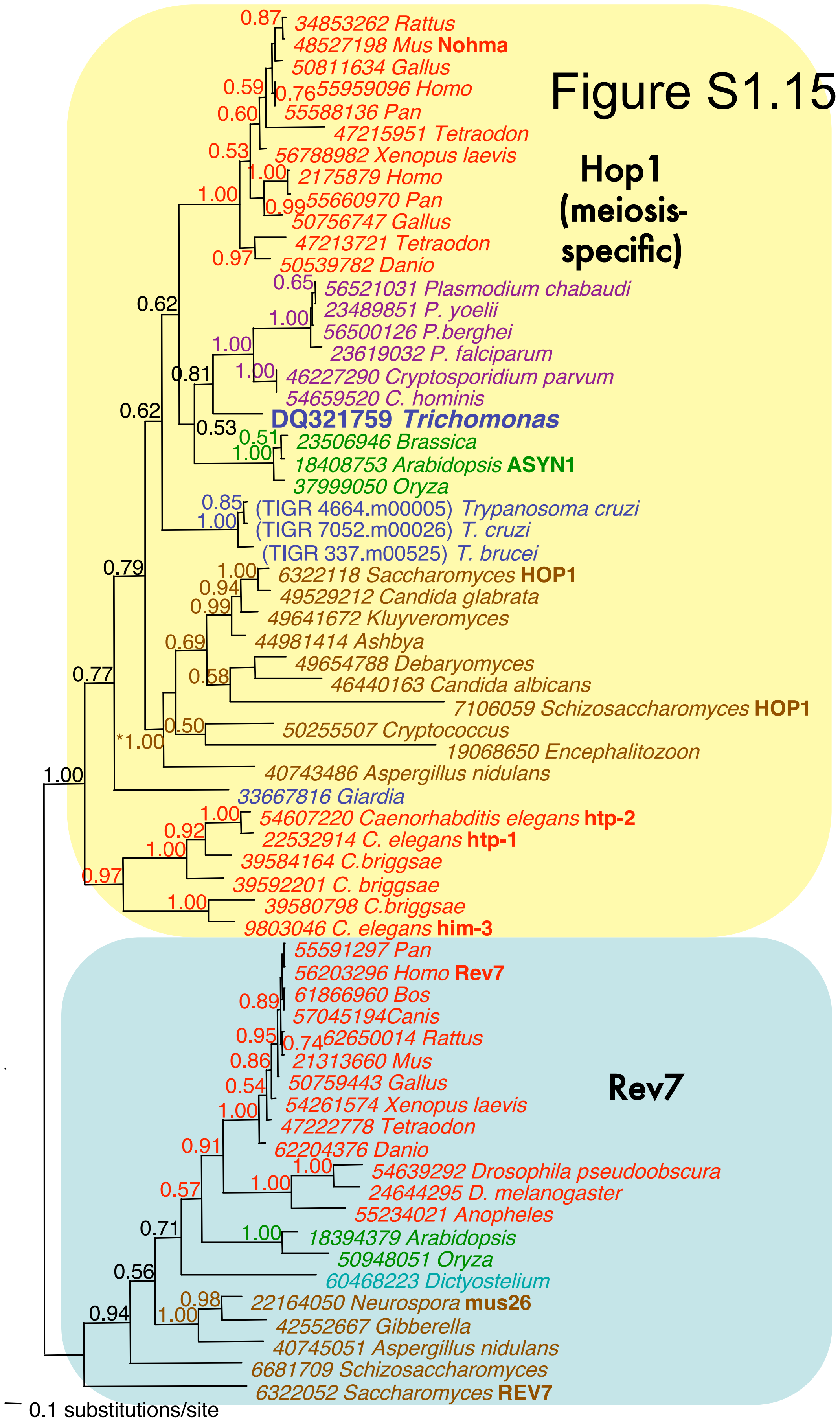

Figure S1.15
Hop1 (meiosis-specific)
Rev7
34853262 Rattus
48527198 Mus Nohma
50811634 Gallus
55959096 Homo
55588136 Pan
47215951 Tetraodon
56788982 Xenopus laevis
2175879 Homo
55660970 Pan
50756747 Gallus
47213721 Tetraodon
50539782 Danio
56521031 Plasmodium chabaudi
23489851 P. yoelii
56500126 P.berghei
23619032 P. falciparum
46227290 Cryptosporidium parvum
54659520 C. hominis
DQ321759 Trichomonas
23506946 Brassica
18408753 Arabidopsis ASYN1
37999050 Oryza
(TIGR 4664.m00005) Trypanosoma cruzi
(TIGR 7052.m00026) T. cruzi
(TIGR 337.m00525) T. brucei
6322118 Saccharomyces HOP1
49529212 Candida glabrata
49641672 Kluyveromyces
44981414 Ashbya
49654788 Debaryomyces
46440163 Candida albicans
7106059 Schizosaccharomyces HOP1
50255507 Cryptococcus
19068650 Encephalitozoon
40743486 Aspergillus nidulans
33667816 Giardia
54607220 Caenorhabditis elegans htp-2
22532914 C. elegans htp-1
39584164 C.briggsae
39592201 C. briggsae
39580798 C.briggsae
9803046 C. elegans him-3
55591297 Pan
56203296 Homo Rev7
61866960 Bos
57045194Canis
62650014 Rattus
21313660 Mus
50759443 Gallus
54261574 Xenopus laevis
47222778 Tetraodon
62204376 Danio
54639292 Drosophila pseudoobscura
24644295 D. melanogaster
55234021 Anopheles
18394379 Arabidopsis
50948051 Oryza
60468223 Dictyostelium
22164050 Neurospora mus26
42552667 Gibberella
40745051 Aspergillus nidulans
6681709 Schizosaccharomyces
6322052 Saccharomyces REV7
0.87
0.59
0.76
0.60
0.53
1.00
0.99
1.00
0.97
0.65
1.00
1.00
1.00
0.62
0.81
0.62
0.53
0.51
1.00
0.85
1.00
0.79
1.00
0.94
0.99
0.69
0.58
0.77
*1.00
0.50
1.00
1.00
0.92
1.00
0.97
1.00
0.89
0.95
0.74
0.86
0.54
1.00
0.91
1.00
1.00
0.57
0.71
1.00
0.56
0.98
0.94
1.00
0.1 substitutions/site

Figure S1.16: Hop1 homologs, unrooted. 188 aligned amino acid sites were analyzed, this consensus topology derived from 940 trees, $\alpha = 3.16$ ($2.29 < \alpha < 4.23$), pI = 0.027 (0.005 < pI < 0.064) and lnL = –9994.27.

Figure S1.16

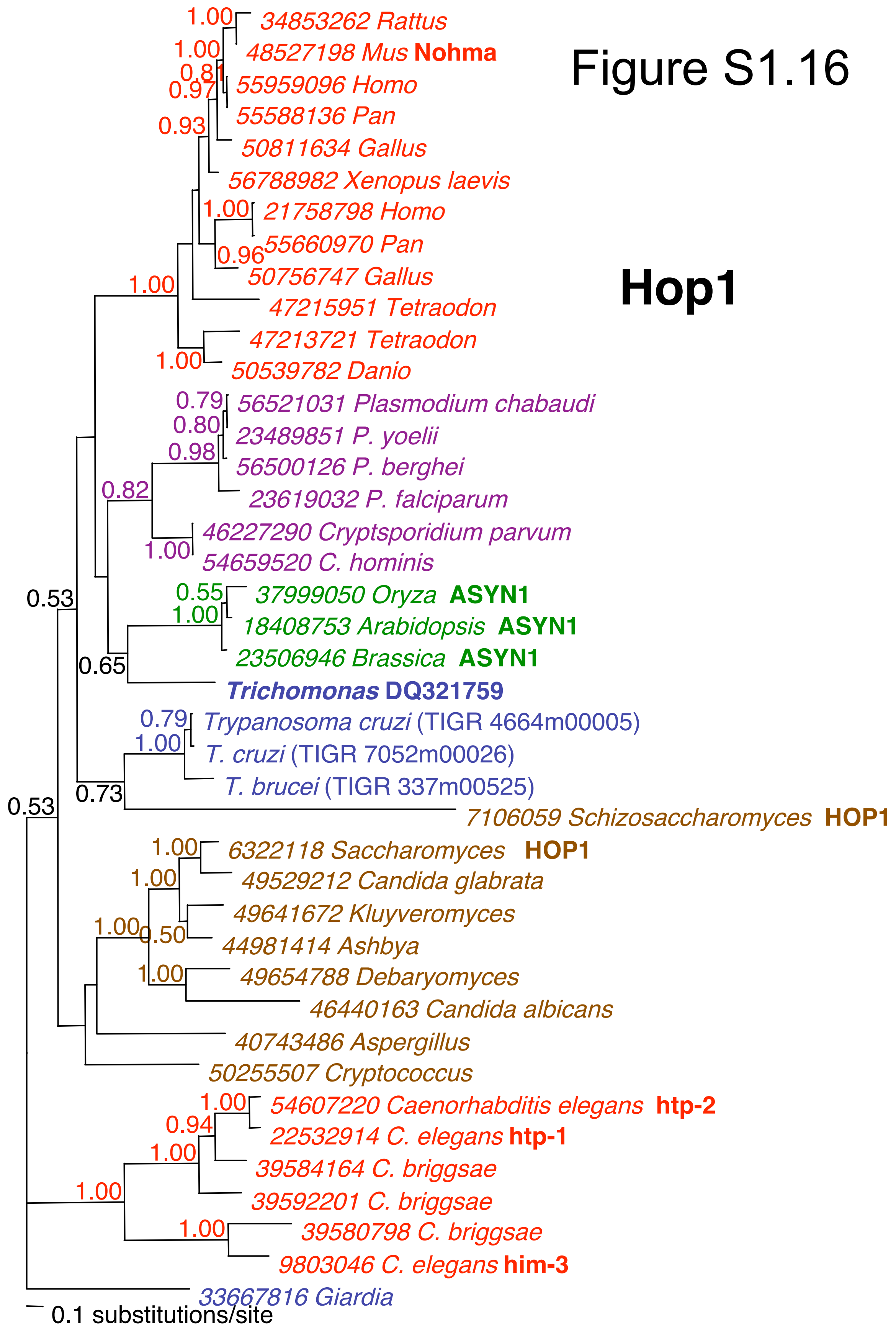

Hop1
1.00
34853262 Rattus
1.00
48527198 Mus Nohma
0.81
0.97
55959096 Homo
55588136 Pan
0.93
50811634 Gallus
56788982 Xenopus laevis
1.00
21758798 Homo
0.96
55660970 Pan
50756747 Gallus
1.00
47215951 Tetraodon
47213721 Tetraodon
1.00
50539782 Danio
0.79
56521031 Plasmodium chabaudi
0.80
23489851 P. yoelii
0.98
56500126 P. berghei
0.82
23619032 P. falciparum
46227290 Cryptsporidium parvum
1.00
54659520 C. hominis
0.55
37999050 Oryza ASYN1
1.00
18408753 Arabidopsis ASYN1
0.53
23506946 Brassica ASYN1
0.65
Trichomonas DQ321759
0.79
Trypanosoma cruzi (TIGR 4664m00005)
1.00
T. cruzi (TIGR 7052m00026)
0.73
T. brucei (TIGR 337m00525)
0.53
7106059 Schizosaccharomyces HOP1
1.00
6322118 Saccharomyces HOP1
1.00
49529212 Candida glabrata
49641672 Kluyveromyces
1.00
0.50
44981414 Ashbya
1.00
49654788 Debaryomyces
46440163 Candida albicans
40743486 Aspergillus
50255507 Cryptococcus
1.00
54607220 Caenorhabditis elegans htp-2
0.94
22532914 C. elegans htp-1
1.00
39584164 C. briggsae
1.00
39592201 C. briggsae
1.00
39580798 C. briggsae
9803046 C. elegans him-3
33667816 Giardia
0.1 substitutions/site

Figure S1.17: Hop2 homologs, **rooted with Mnd1 paralog outgroup.** 153 aligned amino acid sites were analyzed, this consensus topology derived from 950 trees, $\alpha = 4.36$ ($3.02 < \alpha < 5.90$), $pI = 0.13$ ($0.013 < pI < 0.25$) and $\ln L = -16760.34$.

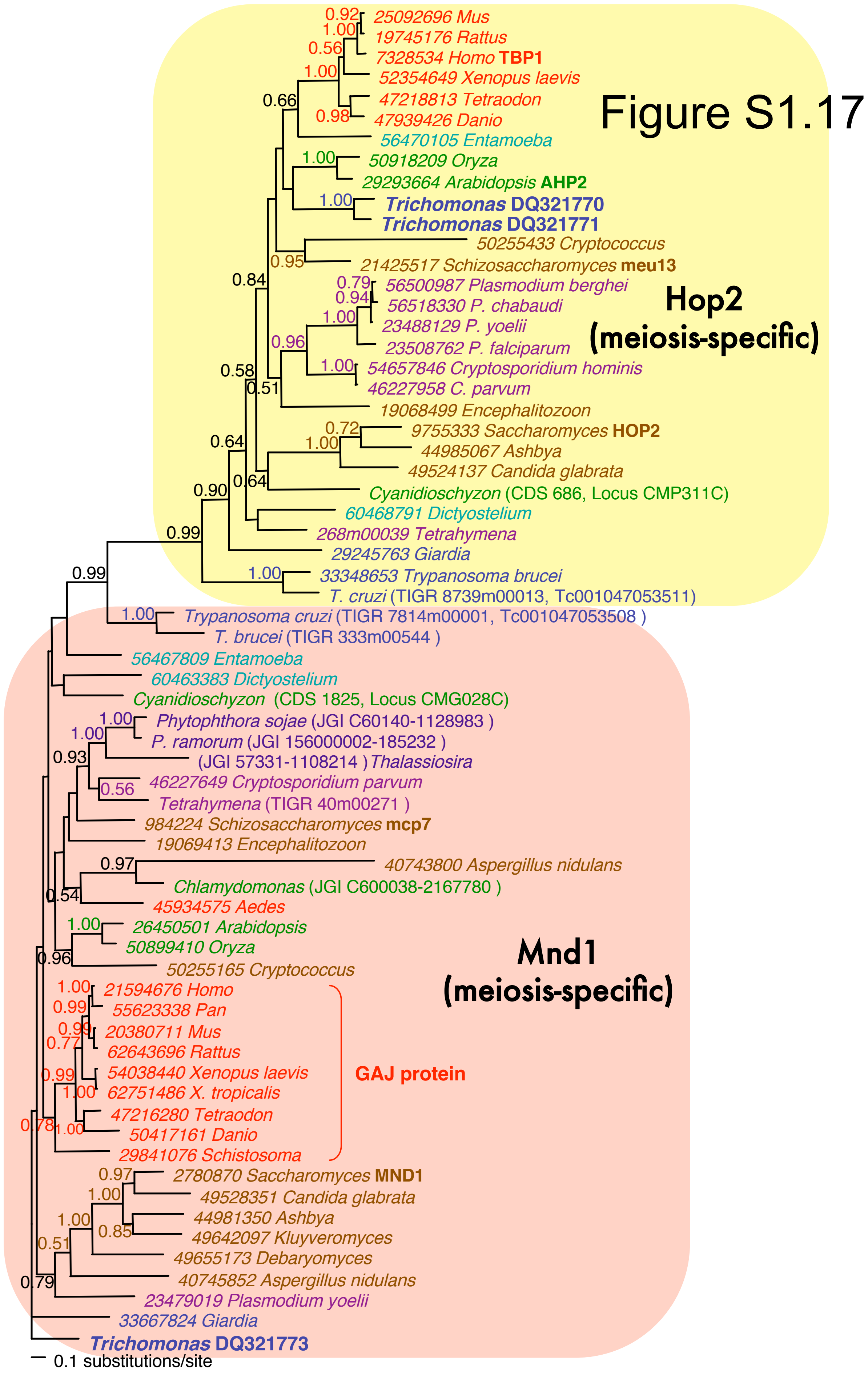

Figure S1.17
Hop2
(meiosis-specific)
Mnd1
(meiosis-specific)
25092696 Mus
19745176 Rattus
7328534 Homo TBP1
52354649 Xenopus laevis
47218813 Tetraodon
47939426 Danio
56470105 Entamoeba
50918209 Oryza
29293664 Arabidopsis AHP2
Trichomonas DQ321770
Trichomonas DQ321771
50255433 Cryptococcus
21425517 Schizosaccharomyces meu13
56500987 Plasmodium berghei
56518330 P. chabaudi
23488129 P. yoelii
23508762 P. falciparum
54657846 Cryptosporidium hominis
46227958 C. parvum
19068499 Encephalitozoon
9755333 Saccharomyces HOP2
44985067 Ashbya
49524137 Candida glabrata
Cyanidioschyzon (CDS 686, Locus CMP311C)
60468791 Dictyostelium
268m00039 Tetrahymena
29245763 Giardia
33348653 Trypanosoma brucei
T. cruzi (TIGR 8739m00013, Tc001047053511)
Trypanosoma cruzi (TIGR 7814m00001, Tc001047053508)
T. brucei (TIGR 333m00544)
56467809 Entamoeba
60463383 Dictyostelium
Cyanidioschyzon (CDS 1825, Locus CMG028C)
Phytophthora sojae (JGI C60140-1128983)
P. ramorum (JGI 156000002-185232)
(JGI 57331-1108214) Thalassiosira
46227649 Cryptosporidium parvum
Tetrahymena (TIGR 40m00271)
984224 Schizosaccharomyces mcp7
19069413 Encephalitozoon
40743800 Aspergillus nidulans
Chlamydomonas (JGI C600038-2167780)
45934575 Aedes
26450501 Arabidopsis
50899410 Oryza
50255165 Cryptococcus
21594676 Homo
55623338 Pan
20380711 Mus
62643696 Rattus
54038440 Xenopus laevis
62751486 X. tropicalis
47216280 Tetraodon
50417161 Danio
29841076 Schistosoma
GAJ protein
2780870 Saccharomyces MND1
49528351 Candida glabrata
44981350 Ashbya
49642097 Kluyveromyces
49655173 Debaryomyces
40745852 Aspergillus nidulans
23479019 Plasmodium yoelii
33667824 Giardia
Trichomonas DQ321773
0.1 substitutions/site

Figure S1.18: Hop2 homologs, unrooted. 167 aligned amino acid sites were analyzed, this consensus topology derived from 950 trees, $\alpha = 3.72$ ($2.66 < \alpha < 5.16$), pI = 0.016 (0.0005 < pI < 0.054) and lnL = –8701.93. Numbers in parentheses represent the percent bootstrap support (if above 50%) estimated with PROML from 100 bootstrap replicates.

Figure S1.18

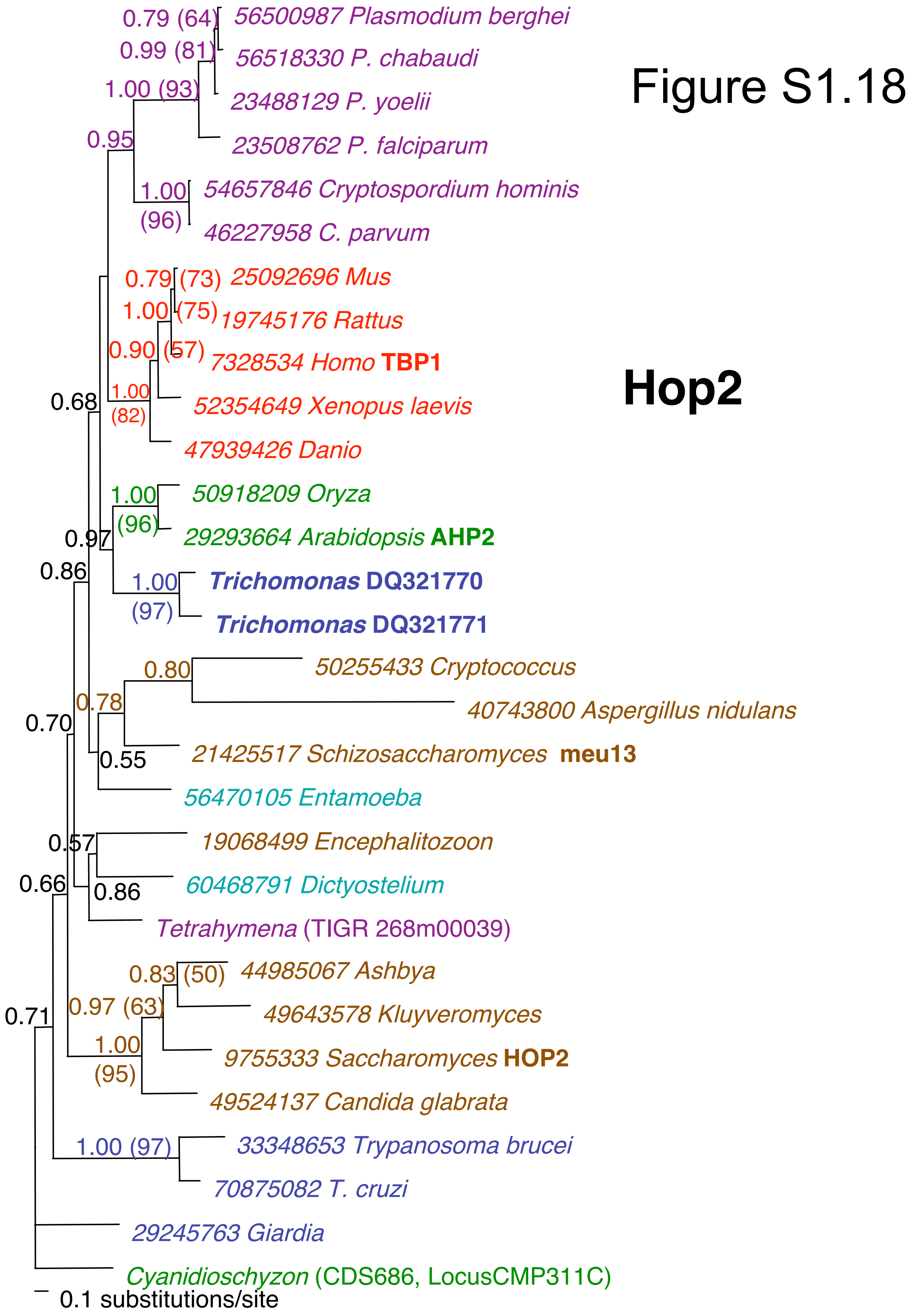
Hop2
0.79 (64)
56500987 Plasmodium berghei
0.99 (81)
56518330 P. chabaudi
1.00 (93)
23488129 P. yoelii
0.95
23508762 P. falciparum
1.00
(96)
54657846 Cryptospordium hominis
46227958 C. parvum
0.79 (73)
25092696 Mus
1.00 (75)
19745176 Rattus
0.90 (57)
7328534 Homo TBP1
1.00
(82)
52354649 Xenopus laevis
0.68
47939426 Danio
1.00
(96)
50918209 Oryza
29293664 Arabidopsis AHP2
0.97
0.86
1.00
(97)
Trichomonas DQ321770
Trichomonas DQ321771
0.80
50255433 Cryptococcus
0.78
40743800 Aspergillus nidulans
0.70
0.55
21425517 Schizosaccharomyces meu13
56470105 Entamoeba
0.57
19068499 Encephalitozoon
0.66
0.86
60468791 Dictyostelium
Tetrahymena (TIGR 268m00039)
0.83 (50)
44985067 Ashbya
0.97 (63)
49643578 Kluyveromyces
0.71
1.00
(95)
9755333 Saccharomyces HOP2
49524137 Candida glabrata
1.00 (97)
33348653 Trypanosoma brucei
70875082 T. cruzi
29245763 Giardia
Cyanidioschyzon (CDS686, LocusCMP311C)
0.1 substitutions/site

Figure S1.19: Mnd1 homologs, unrooted. 202 aligned amino acid sites were analyzed, this consensus topology derived from 900 trees, $\alpha = 2.79$ ($2.15 < \alpha < 3.52$), pI = 0.01 (0.0004 < pI < 0.042) and lnL = –11953.15. Numbers in parentheses represent the percent bootstrap support (if above 50%) estimated with PROML from 100 bootstrap replicates.

Figure S1.19

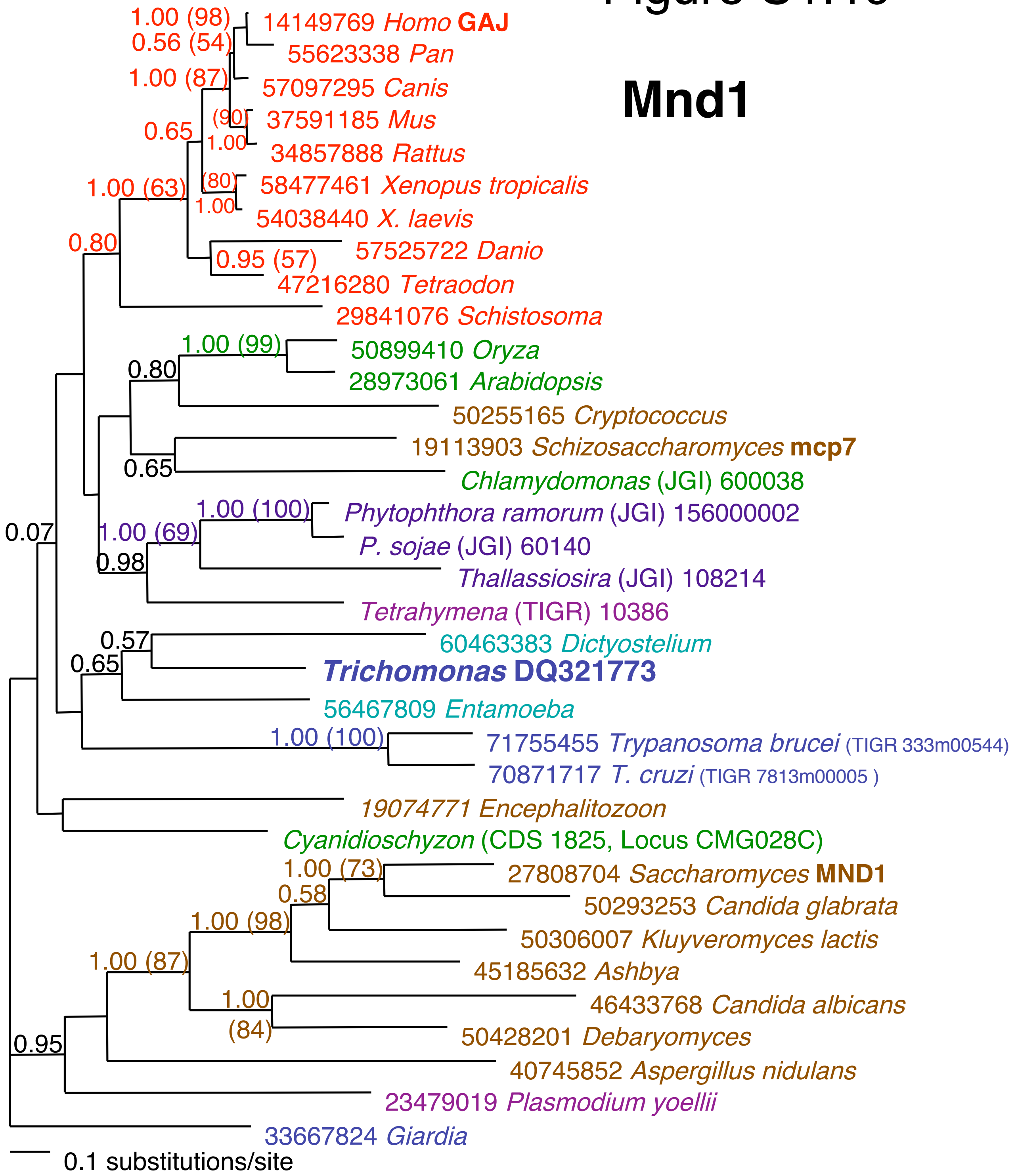

Mnd1
1.00 (98)
0.56 (54)
14149769 Homo GAJ
55623338 Pan
1.00 (87)
57097295 Canis
(90)
1.00
37591185 Mus
34857888 Rattus
0.65
1.00 (63)
(80)
1.00
58477461 Xenopus tropicalis
54038440 X. laevis
0.80
0.95 (57)
57525722 Danio
47216280 Tetraodon
29841076 Schistosoma
1.00 (99)
0.80
50899410 Oryza
28973061 Arabidopsis
50255165 Cryptococcus
19113903 Schizosaccharomyces mcp7
0.65
Chlamydomonas (JGI) 600038
1.00 (100)
Phytophthora ramorum (JGI) 156000002
0.07
1.00 (69)
P. sojae (JGI) 60140
0.98
Thallassiosira (JGI) 108214
Tetrahymena (TIGR) 10386
0.57
60463383 Dictyostelium
0.65
Trichomonas DQ321773
56467809 Entamoeba
1.00 (100)
71755455 Trypanosoma brucei (TIGR 333m00544)
70871717 T. cruzi (TIGR 7813m00005)
19074771 Encephalitozoon
Cyanidioschyzon (CDS 1825, Locus CMG028C)
1.00 (73)
27808704 Saccharomyces MND1
0.58
50293253 Candida glabrata
1.00 (98)
50306007 Kluyveromyces lactis
1.00 (87)
45185632 Ashbya
1.00
46433768 Candida albicans
(84)
50428201 Debaryomyces
0.95
40745852 Aspergillus nidulans
23479019 Plasmodium yoellii
33667824 Giardia
0.1 substitutions/site

Figure S1.20: Rad52 homologs, rooted with Rad59 paralog outgroup. 159 aligned amino acid sites were analyzed, this consensus topology derived from 960 trees, $\alpha = 1.91$ ($1.46 < \alpha < 2.46$), pI = 0.02 (0.0006 < pI < 0.06) and lnL = –9707.78.

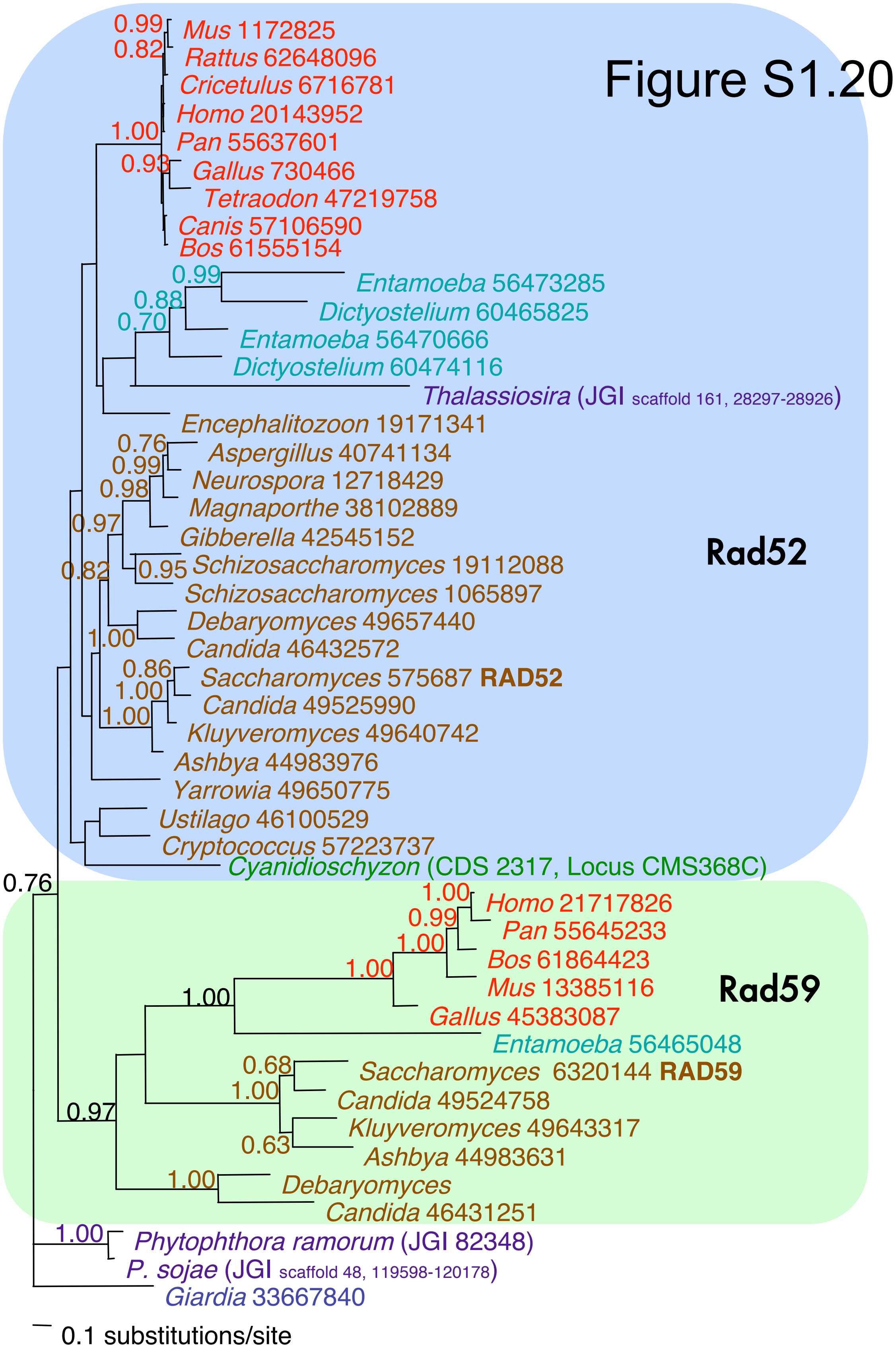

Figure S1.20
Rad52
Rad59
0.99
0.82
Mus 1172825
Rattus 62648096
Cricetulus 6716781
Homo 20143952
1.00
Pan 55637601
0.93
Gallus 730466
Tetraodon 47219758
Canis 57106590
Bos 61555154
0.99
0.88
0.70
Entamoeba 56473285
Dictyostelium 60465825
Entamoeba 56470666
Dictyostelium 60474116
Thalassiosira (JGI scaffold 161, 28297-28926)
Encephalitozoon 19171341
0.76
0.99
0.98
0.97
Aspergillus 40741134
Neurospora 12718429
Magnaporthe 38102889
Gibberella 42545152
0.82
0.95
Schizosaccharomyces 19112088
Schizosaccharomyces 1065897
Debaryomyces 49657440
1.00
Candida 46432572
0.86
1.00
1.00
Saccharomyces 575687 RAD52
Candida 49525990
Kluyveromyces 49640742
Ashbya 44983976
Yarrowia 49650775
Ustilago 46100529
Cryptococcus 57223737
Cyanidioschyzon (CDS 2317, Locus CMS368C)
0.76
1.00
0.99
1.00
1.00
Homo 21717826
Pan 55645233
Bos 61864423
Mus 13385116
1.00
Gallus 45383087
Entamoeba 56465048
0.68
1.00
Saccharomyces 6320144 RAD59
Candida 49524758
0.97
Kluyveromyces 49643317
0.63
Ashbya 44983631
1.00
Debaryomyces
Candida 46431251
1.00
Phytophthora ramorum (JGI 82348)
P. sojae (JGI scaffold 48, 119598-120178)
Giardia 33667840
0.1 substitutions/site

Figure S1.21: RecA homologs, Rad51 and Dmc1, rooted with the archaeal RadA ortholog outgroup. 221 aligned amino acid sites were analyzed, this consensus topology derived from 900 trees, $\alpha = 1.27$ ($0.99 < \alpha < 1.55$), $pI = 0.08$ ($0.04 < pI < 0.13$) and $\ln L = -12602.85$.

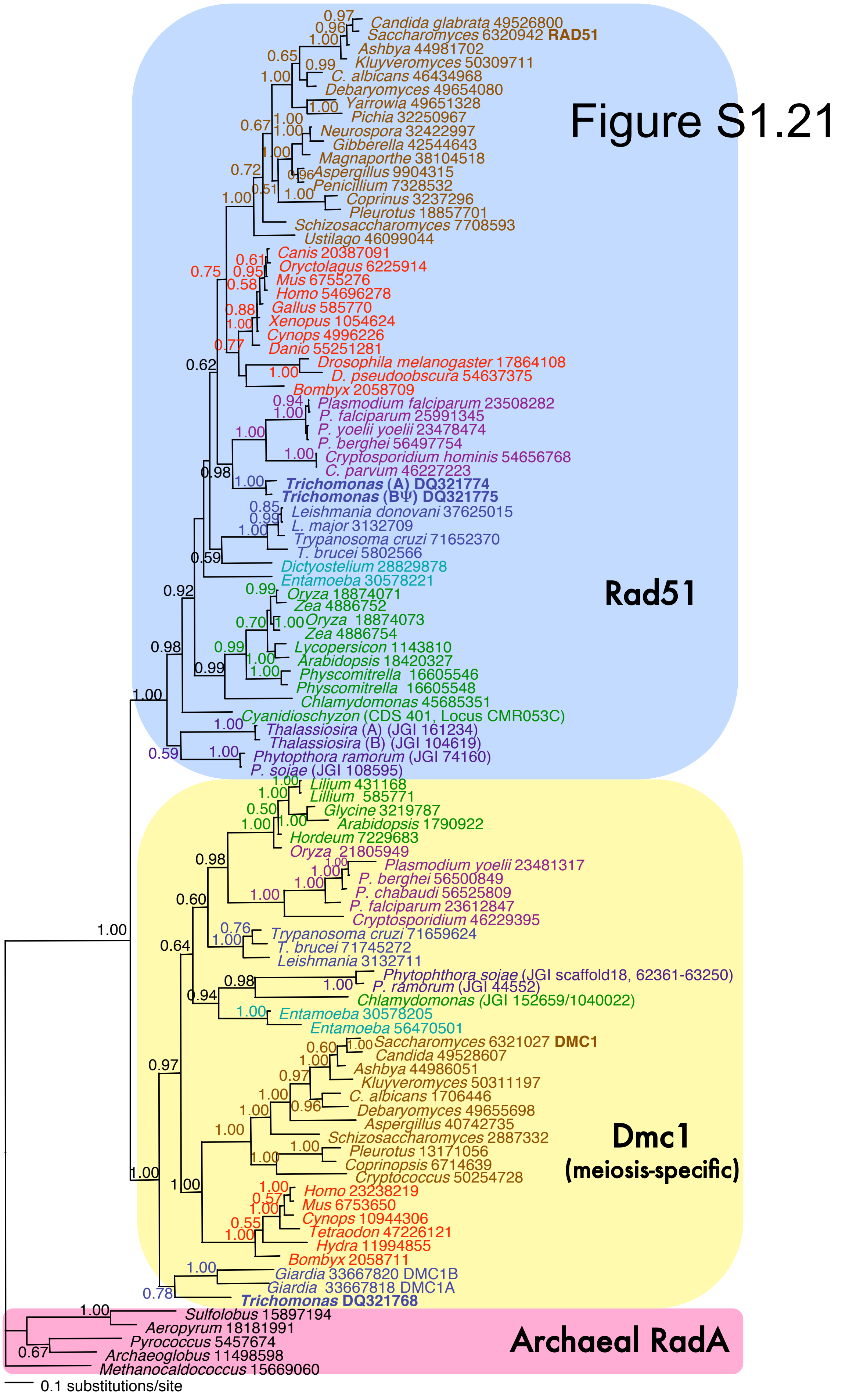

Figure S1.21
Rad51
Candida glabrata 49526800
Saccharomyces 6320942 RAD51
Ashbya 44981702
Kluyveromyces 50309711
C. albicans 46434968
Debaryomyces 49654080
Yarrowia 49651328
Pichia 32250967
Neurospora 32422997
Gibberella 42544643
Magnaporthe 38104518
Aspergillus 9904315
Penicillium 7328532
Coprinus 3237296
Pleurotus 18857701
Schizosaccharomyces 7708593
Ustilago 46099044
Canis 20387091
Oryctolagus 6225914
Mus 6755276
Homo 54696278
Gallus 585770
Xenopus 1054624
Cynops 4996226
Danio 55251281
Drosophila melanogaster 17864108
D. pseudoobscura 54637375
Bombyx 2058709
Plasmodium falciparum 23508282
P. falciparum 25991345
P. yoelii yoelii 23478474
P. berghei 56497754
Cryptosporidium hominis 54656768
C. parvum 46227223
Trichomonas (A) DQ321774
Trichomonas (BΨ) DQ321775
Leishmania donovani 37625015
L. major 3132709
Trypanosoma cruzi 71652370
T. brucei 5802566
Dictyostelium 28829878
Entamoeba 30578221
Oryza 18874071
Zea 4886752
Oryza 18874073
Zea 4886754
Lycopersicon 1143810
Arabidopsis 18420327
Physcomitrella 16605546
Physcomitrella 16605548
Chlamydomonas 45685351
Cyanidioschyzon (CDS 401, Locus CMR053C)
Thalassiosira (A) (JGI 161234)
Thalassiosira (B) (JGI 104619)
Phytopthora ramorum (JGI 74160)
P. sojae (JGI 108595)
Dmc1
(meiosis-specific)
Lilium 431168
Lillium 585771
Glycine 3219787
Arabidopsis 1790922
Hordeum 7229683
Oryza 21805949
Plasmodium yoelii 23481317
P. berghei 56500849
P. chabaudi 56525809
P. falciparum 23612847
Cryptosporidium 46229395
Trypanosoma cruzi 71659624
T. brucei 71745272
Leishmania 3132711
Phytophthora sojae (JGI scaffold18, 62361-63250)
P. ramorum (JGI 44552)
Chlamydomonas (JGI 152659/1040022)
Entamoeba 30578205
Entamoeba 56470501
Saccharomyces 6321027 DMC1
Candida 49528607
Ashbya 44986051
Kluyveromyces 50311197
C. albicans 1706446
Debaryomyces 49655698
Aspergillus 40742735
Schizosaccharomyces 2887332
Pleurotus 13171056
Coprinopsis 6714639
Cryptococcus 50254728
Homo 23238219
Mus 6753650
Cynops 10944306
Tetraodon 47226121
Hydra 11994855
Bombyx 2058711
Giardia 33667820 DMC1B
Giardia 33667818 DMC1A
Trichomonas DQ321768
Archaeal RadA
Sulfolobus 15897194
Aeropyrum 18181991
Pyrococcus 5457674
Archaeoglobus 11498598
Methanocaldococcus 15669060
0.1 substitutions/site

Figure S1.22: Eukaryotic RecA homologs Rad51 and Dmc1, unrooted. 221 aligned amino acid sites were analyzed, this consensus topology derived from 900 trees, $\alpha = 1.20$ ($0.92 < \alpha < 1.52$), $pI = 0.10$ ($0.05 < pI < 0.15$) and $\ln L = -11879.27$.

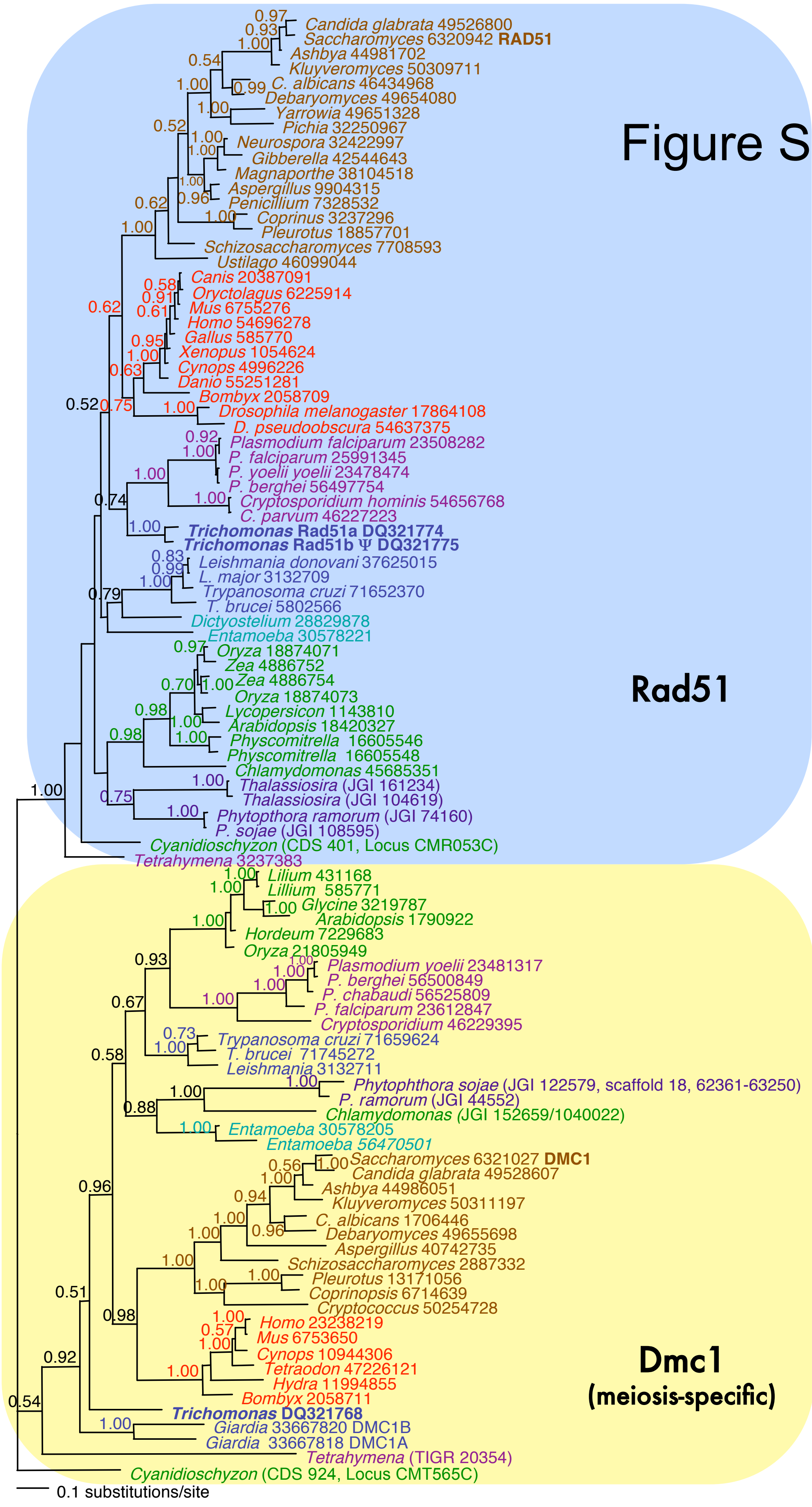
Candida glabrata 49526800
Saccharomyces 6320942 RAD51
Ashbya 44981702
Kluyveromyces 50309711
C. albicans 46434968
Debaryomyces 49654080
Yarrowia 49651328
Pichia 32250967
Neurospora 32422997
Gibberella 42544643
Magnaporthe 38104518
Aspergillus 9904315
Penicillium 7328532
Coprinus 3237296
Pleurotus 18857701
Schizosaccharomyces 7708593
Ustilago 46099044
Canis 20387091
Oryctolagus 6225914
Mus 6755276
Homo 54696278
Gallus 585770
Xenopus 1054624
Cynops 4996226
Danio 55251281
Bombyx 2058709
Drosophila melanogaster 17864108
D. pseudoobscura 54637375
Plasmodium falciparum 23508282
P. falciparum 25991345
P. yoelii yoelii 23478474
P. berghei 56497754
Cryptosporidium hominis 54656768
C. parvum 46227223
Trichomonas Rad51a DQ321774
Trichomonas Rad51b Ψ DQ321775
Leishmania donovani 37625015
L. major 3132709
Trypanosoma cruzi 71652370
T. brucei 5802566
Dictyostelium 28829878
Entamoeba 30578221
Oryza 18874071
Zea 4886752
Zea 4886754
Oryza 18874073
Lycopersicon 1143810
Arabidopsis 18420327
Physcomitrella 16605546
Physcomitrella 16605548
Chlamydomonas 45685351
Thalassiosira (JGI 161234)
Thalassiosira (JGI 104619)
Phytopthora ramorum (JGI 74160)
P. sojae (JGI 108595)
Cyanidioschyzon (CDS 401, Locus CMR053C)
Tetrahymena 3237383
Rad51
Lilium 431168
Lillium 585771
Glycine 3219787
Arabidopsis 1790922
Hordeum 7229683
Oryza 21805949
Plasmodium yoelii 23481317
P. berghei 56500849
P. chabaudi 56525809
P. falciparum 23612847
Cryptosporidium 46229395
Trypanosoma cruzi 71659624
T. brucei 71745272
Leishmania 3132711
Phytophthora sojae (JGI 122579, scaffold 18, 62361-63250)
P. ramorum (JGI 44552)
Chlamydomonas (JGI 152659/1040022)
Entamoeba 30578205
Entamoeba 56470501
Saccharomyces 6321027 DMC1
Candida glabrata 49528607
Ashbya 44986051
Kluyveromyces 50311197
C. albicans 1706446
Debaryomyces 49655698
Aspergillus 40742735
Schizosaccharomyces 2887332
Pleurotus 13171056
Coprinopsis 6714639
Cryptococcus 50254728
Homo 23238219
Mus 6753650
Cynops 10944306
Tetraodon 47226121
Hydra 11994855
Bombyx 2058711
Trichomonas DQ321768
Giardia 33667820 DMC1B
Giardia 33667818 DMC1A
Tetrahymena (TIGR 20354)
Cyanidioschyzon (CDS 924, Locus CMT565C)
Dmc1
(meiosis-specific)
0.1 substitutions/site

Figure S1.22

Figure S1.23: Eukaryotic MutS homologs Msh2, Msh3, Msh4, Msh5 and Msh6, rooted with bacterial MutS ortholog outgroup. 220 aligned amino acid sites were analyzed, this consensus topology derived from 450 trees, $\alpha = 1.16$ ($0.99 < \alpha < 1.33$), $pI = 0.04$ ($0.01 < pI < 0.09$) and $\ln L = -40768.12$.

Figure S1.23

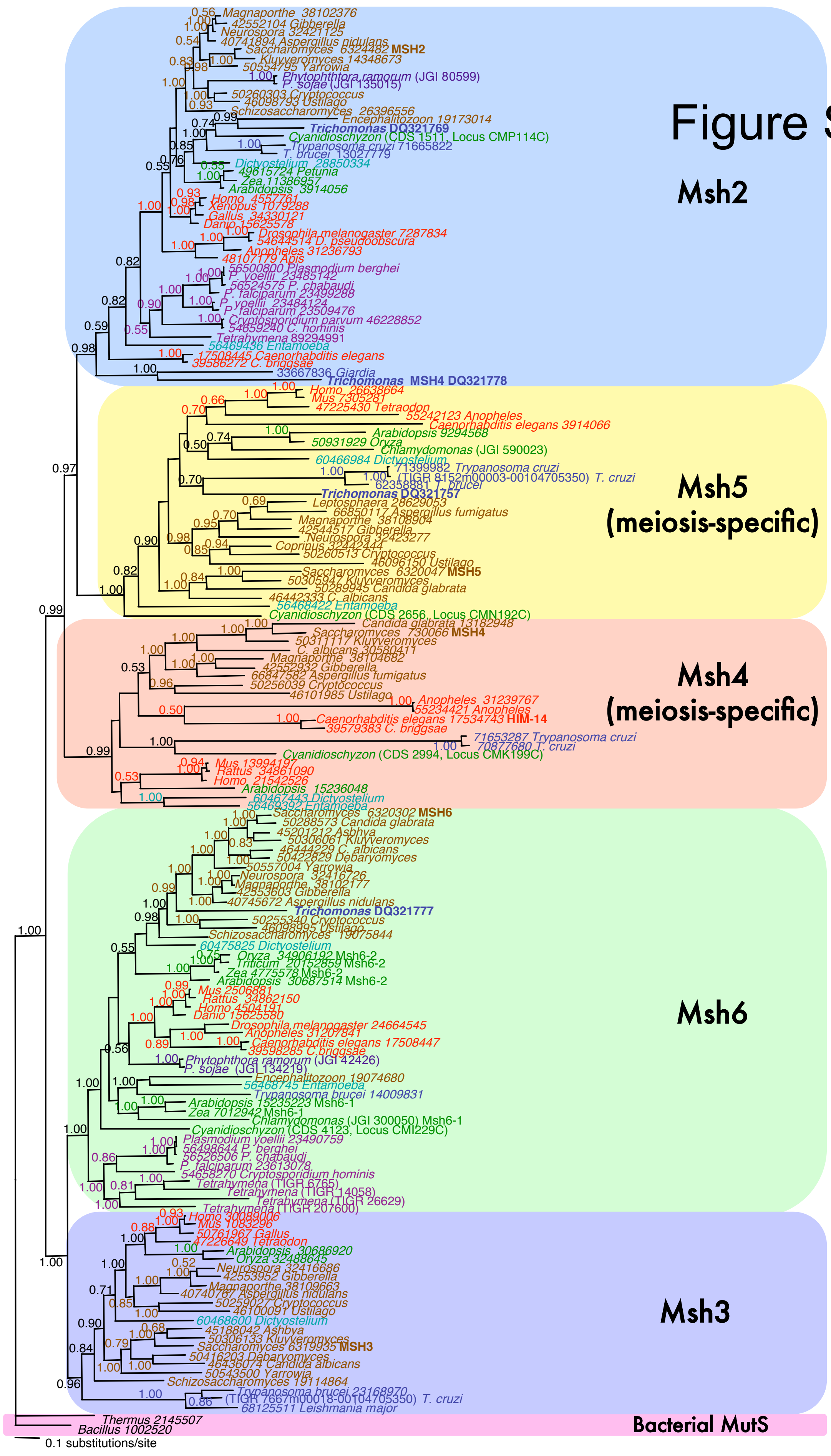

Msh2
Magnaporthe 38102376
42552104 Gibberella
Neurospora 32421125
40741894 Aspergillus nidulans
Saccharomyces 6324482 MSH2
Kluyveromyces 14348673
50554795 Yarrowia
Phytophthora ramorum (JGI 80599)
P. sojae (JGI 135015)
50260303 Cryptococcus
46098793 Ustilago
Schizosaccharomyces 26396556
Encephalitozoon 19173014
Trichomonas DQ321769
Cyanidioschyzon (CDS 1511, Locus CMP114C)
Trypanosoma cruzi 71665822
T. brucei 13027779
Dictyostelium 28850334
49615724 Petunia
Zea 11386957
Arabidopsis 3914056
Homo 4557761
Xenopus 1079288
Gallus 34330121
Danio 15625578
Drosophila melanogaster 7287834
54644514 D. pseudoobscura
Anopheles 31236793
48107179 Apis
56500800 Plasmodium berghei
P. yoelii 23485142
56524575 P. chabaudi
P. falciparum 23499288
P. yoelii 23484124
P. falciparum 23509476
Cryptosporidium parvum 46228852
54659240 C. hominis
Tetrahymena 89294991
56469436 Entamoeba
17508445 Caenorhabditis elegans
39586272 C. briggsae
33667836 Giardia
Trichomonas MSH4 DQ321778
Msh5
(meiosis-specific)
Homo 26638664
Mus 7305281
47225430 Tetraodon
55242123 Anopheles
Caenorhabditis elegans 3914066
Arabidopsis 9294568
50931929 Oryza
Chlamydomonas (JGI 590023)
60466984 Dictyostelium
71399982 Trypanosoma cruzi
(TIGR 8152m00003-00104705350) T. cruzi
62358881 T. brucei
Trichomonas DQ321757
Leptosphaera 28629053
66850117 Aspergillus fumigatus
Magnaporthe 38108904
42544517 Gibberella
Neurospora 32423277
Coprinus 32442444
50260513 Cryptococcus
46096150 Ustilago
Saccharomyces 6320047 MSH5
50305947 Kluyveromyces
50289945 Candida glabrata
46442333 C. albicans
56468422 Entamoeba
Cyanidioschyzon (CDS 2656, Locus CMN192C)
Msh4
(meiosis-specific)
Candida glabrata 13182948
Saccharomyces 730066 MSH4
50311117 Kluyveromyces
C. albicans 30580411
Magnaporthe 38104682
42552932 Gibberella
66847582 Aspergillus fumigatus
50256039 Cryptococcus
46101985 Ustilago
Anopheles 31239767
55234421 Anopheles
Caenorhabditis elegans 17534743 HIM-14
39579383 C. briggsae
71653287 Trypanosoma cruzi
70877680 T. cruzi
Cyanidioschyzon (CDS 2994, Locus CMK199C)
Mus 13994197
Rattus 34861090
Homo 21542526
Arabidopsis 15236048
60467443 Dictyostelium
56469392 Entamoeba
Msh6
Saccharomyces 6320302 MSH6
50288573 Candida glabrata
45201212 Ashbya
50306061 Kluyveromyces
46444229 C. albicans
50422829 Debaryomyces
50557004 Yarrowia
Neurospora 32416726
Magnaporthe 38102177
42553603 Gibberella
40745672 Aspergillus nidulans
Trichomonas DQ321777
50255340 Cryptococcus
46098995 Ustilago
Schizosaccharomyces 19075844
60475825 Dictyostelium
Oryza 34906192 Msh6-2
Triticum 20152859 Msh6-2
Zea 4775578 Msh6-2
Arabidopsis 30687514 Msh6-2
Mus 2506881
Rattus 34862150
Homo 4504191
Danio 15625580
Drosophila melanogaster 24664545
Anopheles 31207841
Caenorhabditis elegans 17508447
39598285 C.briggsae
Phytophthora ramorum (JGI 42426)
P. sojae (JGI 134219)
Encephalitozoon 19074680
56468745 Entamoeba
Trypanosoma brucei 14009831
Arabidopsis 15235223 Msh6-1
Zea 7012942 Msh6-1
Chlamydomonas (JGI 300050) Msh6-1
Cyanidioschyzon (CDS 4123, Locus CMI229C)
Plasmodium yoelii 23490759
56498644 P. berghei
56526506 P. chabaudi
P. falciparum 23613078
54658270 Cryptosporidium hominis
Tetrahymena (TIGR 6765)
Tetrahymena (TIGR 14058)
Tetrahymena (TIGR 26629)
Tetrahymena (TIGR 207600)
Msh3
Homo 30089006
Mus 1083296
50761967 Gallus
47226649 Tetraodon
Arabidopsis 30686920
Oryza 32488845
Neurospora 32416686
42553952 Gibberella
Magnaporthe 38109663
40740787 Aspergillus nidulans
50259027 Cryptococcus
46100091 Ustilago
60468600 Dictyostelium
45188042 Ashbya
50306133 Kluyveromyces
Saccharomyces 6319935 MSH3
50416203 Debaryomyces
46436074 Candida albicans
50543500 Yarrowia
Schizosaccharomyces 19114864
Trypanosoma brucei 23168970
(TIGR 7667m00018-00104705350) T. cruzi
68125511 Leishmania major
Thermus 2145507
Bacillus 1002520
Bacterial MutS
0.1 substitutions/site

Figure S1.24: Meiotic MutS homologs Msh4 and Msh5 arbitrarily rooted with Msh2. 375 aligned amino acid sites were analyzed, this consensus topology derived from 965 trees, $\alpha = 1.66$ ($1.44 < \alpha < 1.88$), pI = 0.03 (0.009 < pI < 0.06) and lnL = –44872.06.

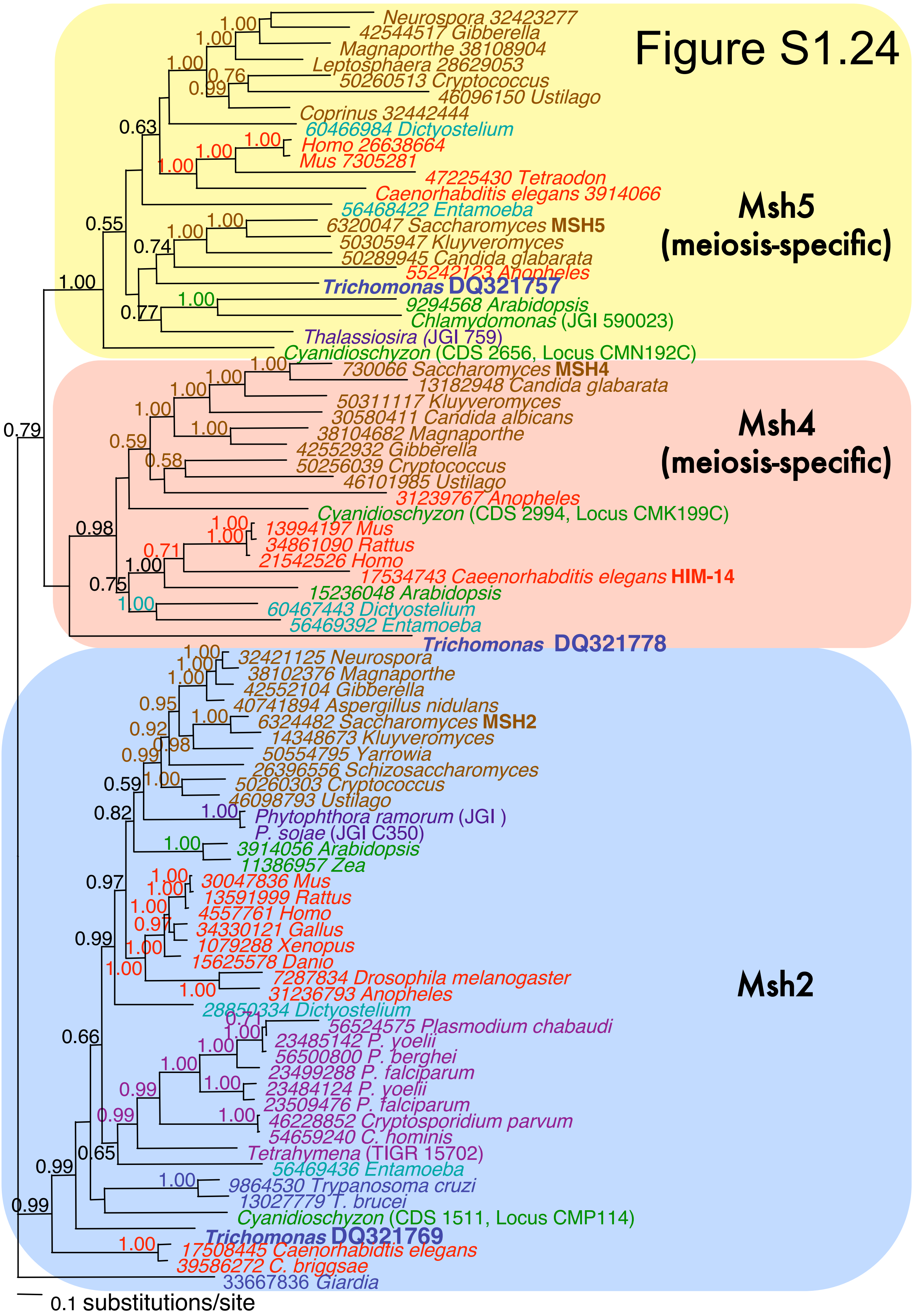
Neurospora 32423277
42544517 Gibberella
Magnaporthe 38108904
Leptosphaera 28629053
50260513 Cryptococcus
46096150 Ustilago
Coprinus 32442444
60466984 Dictyostelium
Homo 26638664
Mus 7305281
47225430 Tetraodon
Caenorhabditis elegans 3914066
56468422 Entamoeba
6320047 Saccharomyces MSH5
50305947 Kluyveromyces
50289945 Candida glabarata
55242123 Anopheles
Trichomonas DQ321757
9294568 Arabidopsis
Chlamydomonas (JGI 590023)
Thalassiosira (JGI 759)
Cyanidioschyzon (CDS 2656, Locus CMN192C)
Msh5
(meiosis-specific)
730066 Saccharomyces MSH4
13182948 Candida glabarata
50311117 Kluyveromyces
30580411 Candida albicans
38104682 Magnaporthe
42552932 Gibberella
50256039 Cryptococcus
46101985 Ustilago
31239767 Anopheles
Cyanidioschyzon (CDS 2994, Locus CMK199C)
13994197 Mus
34861090 Rattus
21542526 Homo
17534743 Caeenorhabditis elegans HIM-14
15236048 Arabidopsis
60467443 Dictyostelium
56469392 Entamoeba
Msh4
(meiosis-specific)
Trichomonas DQ321778
32421125 Neurospora
38102376 Magnaporthe
42552104 Gibberella
40741894 Aspergillus nidulans
6324482 Saccharomyces MSH2
14348673 Kluyveromyces
50554795 Yarrowia
26396556 Schizosaccharomyces
50260303 Cryptococcus
46098793 Ustilago
Phytophthora ramorum (JGI)
P. sojae (JGI C350)
3914056 Arabidopsis
11386957 Zea
30047836 Mus
13591999 Rattus
4557761 Homo
34330121 Gallus
1079288 Xenopus
15625578 Danio
7287834 Drosophila melanogaster
31236793 Anopheles
28850334 Dictyostelium
56524575 Plasmodium chabaudi
23485142 P. yoelii
56500800 P. berghei
23499288 P. falciparum
23484124 P. yoelii
23509476 P. falciparum
46228852 Cryptosporidium parvum
54659240 C. hominis
Tetrahymena (TIGR 15702)
56469436 Entamoeba
9864530 Trypanosoma cruzi
13027779 T. brucei
Cyanidioschyzon (CDS 1511, Locus CMP114)
Trichomonas DQ321769
17508445 Caenorhabditis elegans
39586272 C. briggsae
33667836 Giardia
Msh2
0.1 substitutions/site

Figure S1.24

Figure S1.25: Eukaryotic MutL homologs Mlh1, Mlh2, Mlh3 and Pms1, rooted with prokaryotic MutL ortholog outgroup. 277 aligned amino acid sites were analyzed, this consensus topology derived from 400 trees, $\alpha = 1.39$ ($1.22 < \alpha < 1.58$), $pI = 0.052$ ($0.014 < pI < 0.10$) and $lnL = -51467.18$.

Figure S1.25

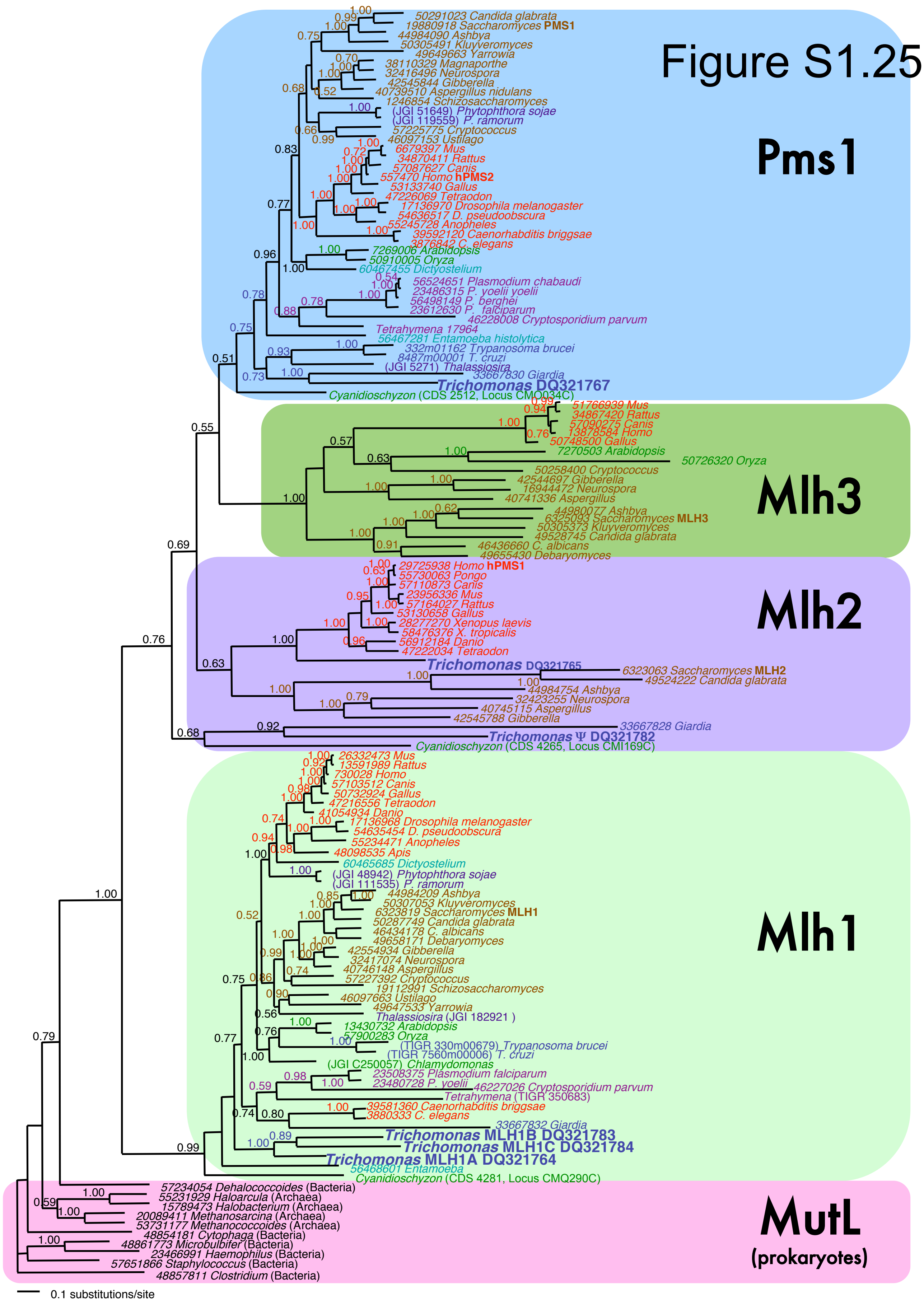

Pms1
50291023 Candida glabrata
19880918 Saccharomyces PMS1
44984090 Ashbya
50305491 Kluyveromyces
49649663 Yarrowia
38110329 Magnaporthe
32416496 Neurospora
42545844 Gibberella
40739510 Aspergillus nidulans
1246854 Schizosaccharomyces
(JGI 51649) Phytophthora sojae
(JGI 119559) P. ramorum
57225775 Cryptococcus
46097153 Ustilago
6679397 Mus
34870411 Rattus
57087627 Canis
557470 Homo hPMS2
53133740 Gallus
47226069 Tetraodon
17136970 Drosophila melanogaster
54636517 D. pseudoobscura
55245728 Anopheles
39592120 Caenorhabditis briggsae
3876842 C. elegans
7269006 Arabidopsis
50910005 Oryza
60467455 Dictyostelium
56524651 Plasmodium chabaudi
23486315 P. yoelii yoelii
56498149 P. berghei
23612630 P. falciparum
46228008 Cryptosporidium parvum
Tetrahymena 17964
56467281 Entamoeba histolytica
332m01162 Trypanosoma brucei
8487m00001 T. cruzi
(JGI 5271) Thalassiosira
33667830 Giardia
Trichomonas DQ321767
Cyanidioschyzon (CDS 2512, Locus CMO034C)
Mlh3
51766939 Mus
34867420 Rattus
57090275 Canis
13878584 Homo
50748500 Gallus
7270503 Arabidopsis
50726320 Oryza
50258400 Cryptococcus
42544697 Gibberella
16944472 Neurospora
40741336 Aspergillus
44980077 Ashbya
6325093 Saccharomyces MLH3
50305373 Kluyveromyces
49528745 Candida glabrata
46436660 C. albicans
49655430 Debaryomyces
Mlh2
29725938 Homo hPMS1
55730063 Pongo
57110873 Canis
23956336 Mus
57164027 Rattus
53130658 Gallus
28277270 Xenopus laevis
58476376 X. tropicalis
56912184 Danio
47222034 Tetraodon
Trichomonas DQ321765
6323063 Saccharomyces MLH2
49524222 Candida glabrata
44984754 Ashbya
32423255 Neurospora
40745115 Aspergillus
42545788 Gibberella
33667828 Giardia
Trichomonas Ψ DQ321782
Cyanidioschyzon (CDS 4265, Locus CMI169C)
Mlh1
26332473 Mus
13591989 Rattus
730028 Homo
57103512 Canis
50732924 Gallus
47216556 Tetraodon
41054934 Danio
17136968 Drosophila melanogaster
54635454 D. pseudoobscura
55234471 Anopheles
48098535 Apis
60465685 Dictyostelium
(JGI 48942) Phytophthora sojae
(JGI 111535) P. ramorum
44984209 Ashbya
50307053 Kluyveromyces
6323819 Saccharomyces MLH1
50287749 Candida glabrata
46434178 C. albicans
49658171 Debaryomyces
42554934 Gibberella
32417074 Neurospora
40746148 Aspergillus
57227392 Cryptococcus
19112991 Schizosaccharomyces
46097663 Ustilago
49647533 Yarrowia
Thalassiosira (JGI 182921)
13430732 Arabidopsis
57900283 Oryza
(TIGR 330m00679) Trypanosoma brucei
(TIGR 7560m00006) T. cruzi
(JGI C250057) Chlamydomonas
23508375 Plasmodium falciparum
23480728 P. yoelii
46227026 Cryptosporidium parvum
Tetrahymena (TIGR 350683)
39581360 Caenorhabditis briggsae
3880333 C. elegans
33667832 Giardia
Trichomonas MLH1B DQ321783
Trichomonas MLH1C DQ321784
Trichomonas MLH1A DQ321764
56468601 Entamoeba
Cyanidioschyzon (CDS 4281, Locus CMQ290C)
MutL
(prokaryotes)
57234054 Dehalococcoides (Bacteria)
55231929 Haloarcula (Archaea)
15789473 Halobacterium (Archaea)
20089411 Methanosarcina (Archaea)
53731177 Methanococcoides (Archaea)
48854181 Cytophaga (Bacteria)
48861773 Microbulbifer (Bacteria)
23466991 Haemophilus (Bacteria)
57651866 Staphylococcus (Bacteria)
48857811 Clostridium (Bacteria)
0.1 substitutions/site

Figure S1.26: MutL homologs Mlh1, Mlh2, and Pms1, arbitrarily rooted with Mlh3. 293 aligned amino acid sites were analyzed, this consensus topology derived from 550 trees, $\alpha = 1.58$ ($1.39 < \alpha < 1.80$), pI = 0.02 (0.0008 < pI < 0.07) and lnL = –52236.93.

Figure S1.26

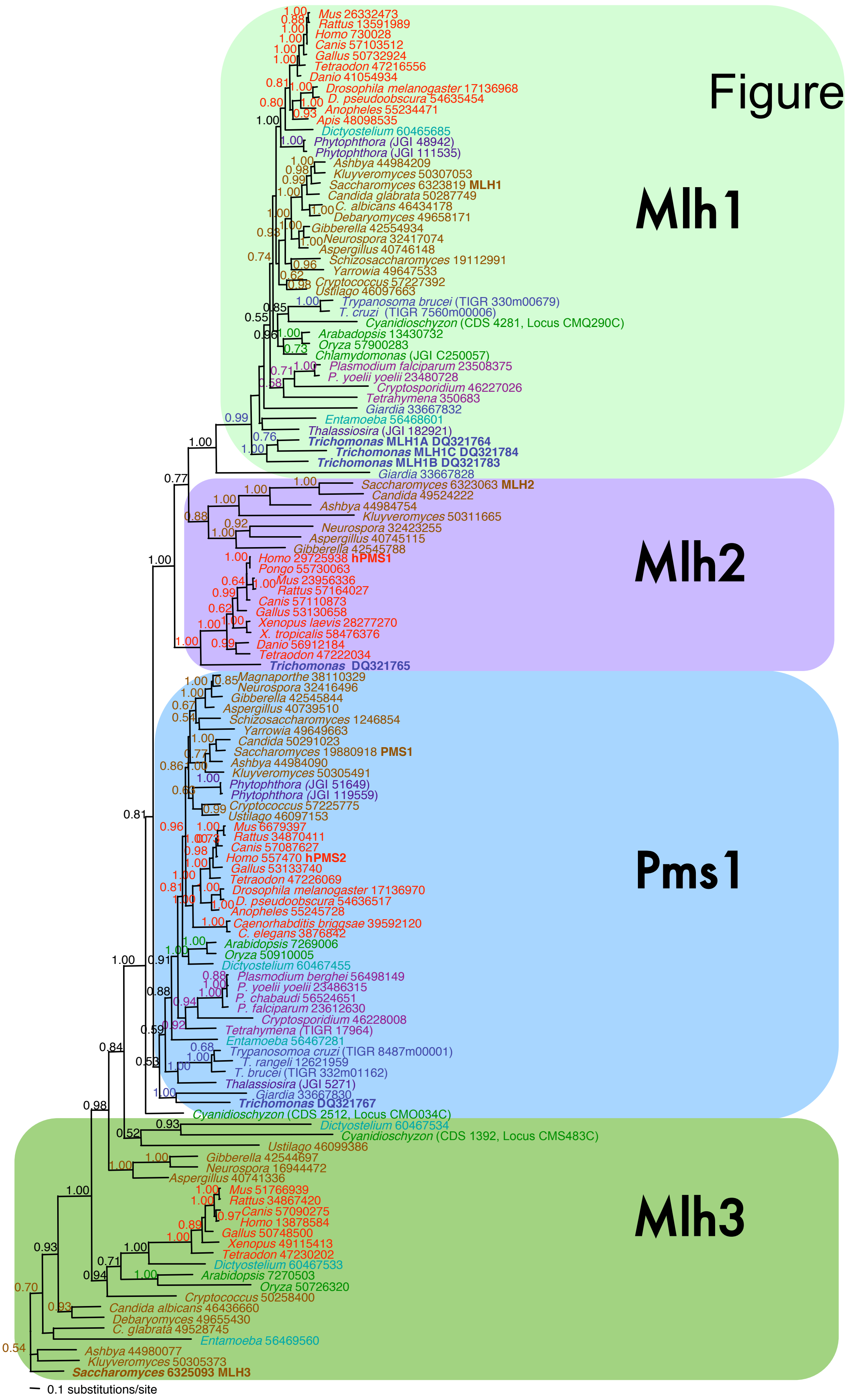

Mlh1
Mlh2
Pms1
Mlh3
Mus 26332473
Rattus 13591989
Homo 730028
Canis 57103512
Gallus 50732924
Tetraodon 47216556
Danio 41054934
Drosophila melanogaster 17136968
D. pseudoobscura 54635454
Anopheles 55234471
Apis 48098535
Dictyostelium 60465685
Phytophthora (JGI 48942)
Phytophthora (JGI 111535)
Ashbya 44984209
Kluyveromyces 50307053
Saccharomyces 6323819 MLH1
Candida glabrata 50287749
C. albicans 46434178
Debaryomyces 49658171
Gibberella 42554934
Neurospora 32417074
Aspergillus 40746148
Schizosaccharomyces 19112991
Yarrowia 49647533
Cryptococcus 57227392
Ustilago 46097663
Trypanosoma brucei (TIGR 330m00679)
T. cruzi (TIGR 7560m00006)
Cyanidioschyzon (CDS 4281, Locus CMQ290C)
Arabadopsis 13430732
Oryza 57900283
Chlamydomonas (JGI C250057)
Plasmodium falciparum 23508375
P. yoelii yoelii 23480728
Cryptosporidium 46227026
Tetrahymena 350683
Giardia 33667832
Entamoeba 56468601
Thalassiosira (JGI 182921)
Trichomonas MLH1A DQ321764
Trichomonas MLH1C DQ321784
Trichomonas MLH1B DQ321783
Giardia 33667828
Saccharomyces 6323063 MLH2
Candida 49524222
Ashbya 44984754
Kluyveromyces 50311665
Neurospora 32423255
Aspergillus 40745115
Gibberella 42545788
Homo 29725938 hPMS1
Pongo 55730063
Mus 23956336
Rattus 57164027
Canis 57110873
Gallus 53130658
Xenopus laevis 28277270
X. tropicalis 58476376
Danio 56912184
Tetraodon 47222034
Trichomonas DQ321765
Magnaporthe 38110329
Neurospora 32416496
Gibberella 42545844
Aspergillus 40739510
Schizosaccharomyces 1246854
Yarrowia 49649663
Candida 50291023
Saccharomyces 19880918 PMS1
Ashbya 44984090
Kluyveromyces 50305491
Phytophthora (JGI 51649)
Phytophthora (JGI 119559)
Cryptococcus 57225775
Ustilago 46097153
Mus 6679397
Rattus 34870411
Canis 57087627
Homo 557470 hPMS2
Gallus 53133740
Tetraodon 47226069
Drosophila melanogaster 17136970
D. pseudoobscura 54636517
Anopheles 55245728
Caenorhabditis briggsae 39592120
C. elegans 3876842
Arabidopsis 7269006
Oryza 50910005
Dictyostelium 60467455
Plasmodium berghei 56498149
P. yoelii yoelii 23486315
P. chabaudi 56524651
P. falciparum 23612630
Cryptosporidium 46228008
Tetrahymena (TIGR 17964)
Entamoeba 56467281
Trypanosomoa cruzi (TIGR 8487m00001)
T. rangeli 12621959
T. brucei (TIGR 332m01162)
Thalassiosira (JGI 5271)
Giardia 33667830
Trichomonas DQ321767
Cyanidioschyzon (CDS 2512, Locus CMO034C)
Dictyostelium 60467534
Cyanidioschyzon (CDS 1392, Locus CMS483C)
Ustilago 46099386
Gibberella 42544697
Neurospora 16944472
Aspergillus 40741336
Mus 51766939
Rattus 34867420
Canis 57090275
Homo 13878584
Gallus 50748500
Xenopus 49115413
Tetraodon 47230202
Dictyostelium 60467533
Arabidopsis 7270503
Oryza 50726320
Cryptococcus 50258400
Candida albicans 46436660
Debaryomyces 49655430
C. glabrata 49528745
Entamoeba 56469560
Ashbya 44980077
Kluyveromyces 50305373
Saccharomyces 6325093 MLH3
0.1 substitutions/site

Figure S1.27: MutL homologs Mlh2 and Pms1, arbitrarily rooted with Mlh1. 226 aligned amino acid sites were analyzed, this consensus topology derived from 800 trees, $\alpha = 1.39$ ($1.19 < \alpha < 1.61$), pI = 0.05 (0.015 < pI < 0.09) and lnL = –29759.05.

Figure S1.27

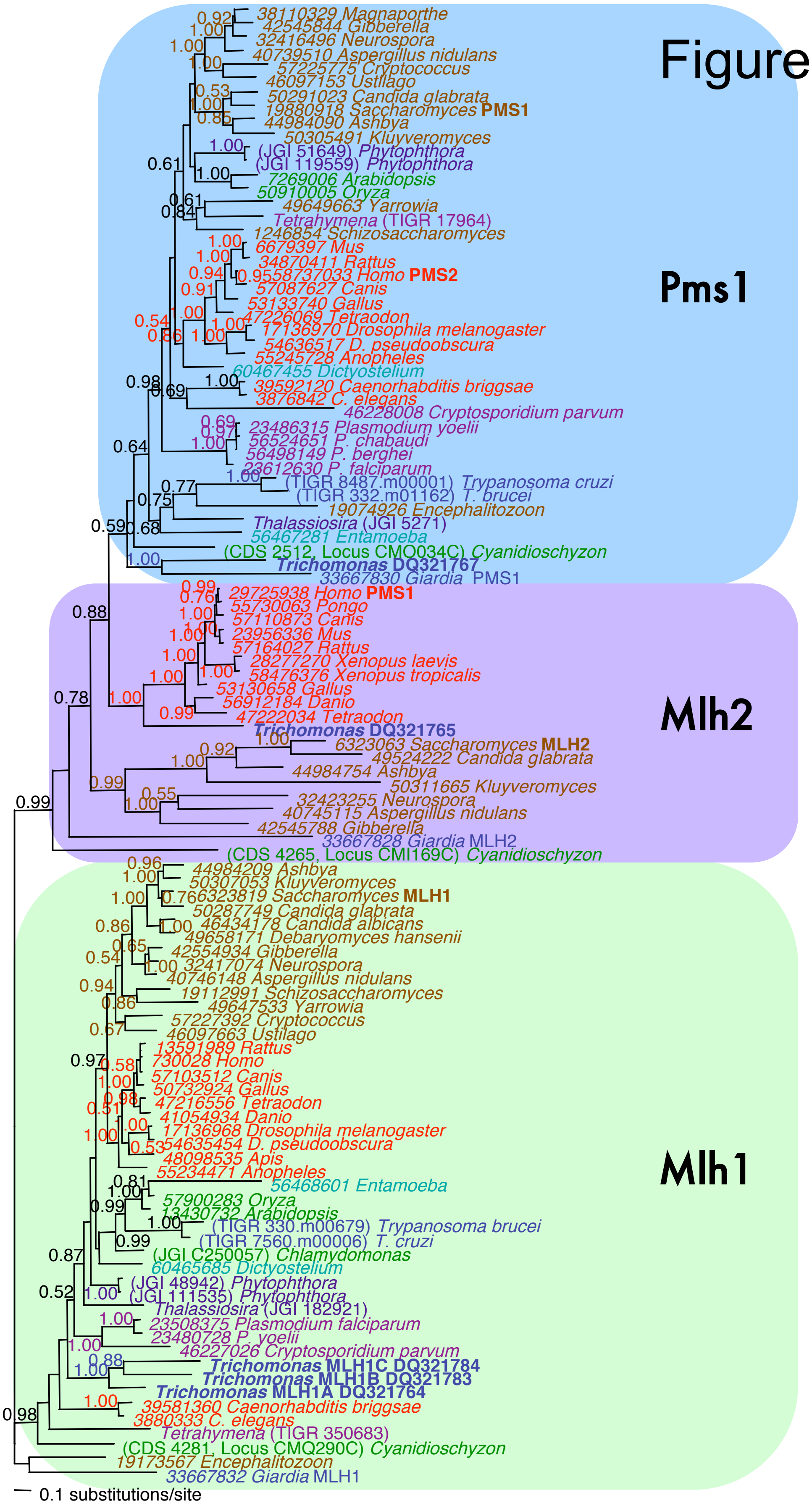

Pms1
Mlh2
Mlh1
38110329 Magnaporthe
42545844 Gibberella
32416496 Neurospora
40739510 Aspergillus nidulans
57225775 Cryptococcus
46097153 Ustilago
50291023 Candida glabrata
19880918 Saccharomyces PMS1
44984090 Ashbya
50305491 Kluyveromyces
(JGI 51649) Phytophthora
(JGI 119559) Phytophthora
7269006 Arabidopsis
50910005 Oryza
49649663 Yarrowia
Tetrahymena (TIGR 17964)
1246854 Schizosaccharomyces
6679397 Mus
34870411 Rattus
0.9558737033 Homo PMS2
57087627 Canis
53133740 Gallus
47226069 Tetraodon
17136970 Drosophila melanogaster
54636517 D. pseudoobscura
55245728 Anopheles
60467455 Dictyostelium
39592120 Caenorhabditis briggsae
3876842 C. elegans
46228008 Cryptosporidium parvum
23486315 Plasmodium yoelii
56524651 P. chabaudi
56498149 P. berghei
23612630 P. falciparum
(TIGR 8487.m00001) Trypanosoma cruzi
(TIGR 332.m01162) T. brucei
19074926 Encephalitozoon
Thalassiosira (JGI 5271)
56467281 Entamoeba
(CDS 2512, Locus CMO034C) Cyanidioschyzon
Trichomonas DQ321767
33667830 Giardia PMS1
29725938 Homo PMS1
55730063 Pongo
57110873 Canis
23956336 Mus
57164027 Rattus
28277270 Xenopus laevis
58476376 Xenopus tropicalis
53130658 Gallus
56912184 Danio
47222034 Tetraodon
Trichomonas DQ321765
6323063 Saccharomyces MLH2
49524222 Candida glabrata
44984754 Ashbya
50311665 Kluyveromyces
32423255 Neurospora
40745115 Aspergillus nidulans
42545788 Gibberella
33667828 Giardia MLH2
(CDS 4265, Locus CMI169C) Cyanidioschyzon
44984209 Ashbya
50307053 Kluyveromyces
0.766323819 Saccharomyces MLH1
50287749 Candida glabrata
46434178 Candida albicans
49658171 Debaryomyces hansenii
42554934 Gibberella
32417074 Neurospora
40746148 Aspergillus nidulans
19112991 Schizosaccharomyces
49647533 Yarrowia
57227392 Cryptococcus
46097663 Ustilago
13591989 Rattus
730028 Homo
57103512 Canis
50732924 Gallus
47216556 Tetraodon
41054934 Danio
17136968 Drosophila melanogaster
54635454 D. pseudoobscura
48098535 Apis
55234471 Anopheles
56468601 Entamoeba
57900283 Oryza
13430732 Arabidopsis
(TIGR 330.m00679) Trypanosoma brucei
(TIGR 7560.m00006) T. cruzi
(JGI C250057) Chlamydomonas
60465685 Dictyostelium
(JGI 48942) Phytophthora
(JGI 111535) Phytophthora
Thalassiosira (JGI 182921)
23508375 Plasmodium falciparum
23480728 P. yoelii
46227026 Cryptosporidium parvum
Trichomonas MLH1C DQ321784
Trichomonas MLH1B DQ321783
Trichomonas MLH1A DQ321764
39581360 Caenorhabditis briggsae
3880333 C. elegans
Tetrahymena (TIGR 350683)
(CDS 4281, Locus CMQ290C) Cyanidioschyzon
19173567 Encephalitozoon
33667832 Giardia MLH1
0.1 substitutions/site

Figure S1.28: Mer3 homologs, rooted with prokaryotic ortholog outgroup. 555 aligned amino acid sites were analyzed, this consensus topology derived from 550 trees, $\alpha = 1.28$ ($1.14 < \alpha < 1.42$), pI = 0.03 (0.01 < pI < 0.05) and lnL = –62607.95. Sequences are identified by either their NCBI GI numbers or alphanumeric UNIPROT accession numbers (www.uniprot.org).

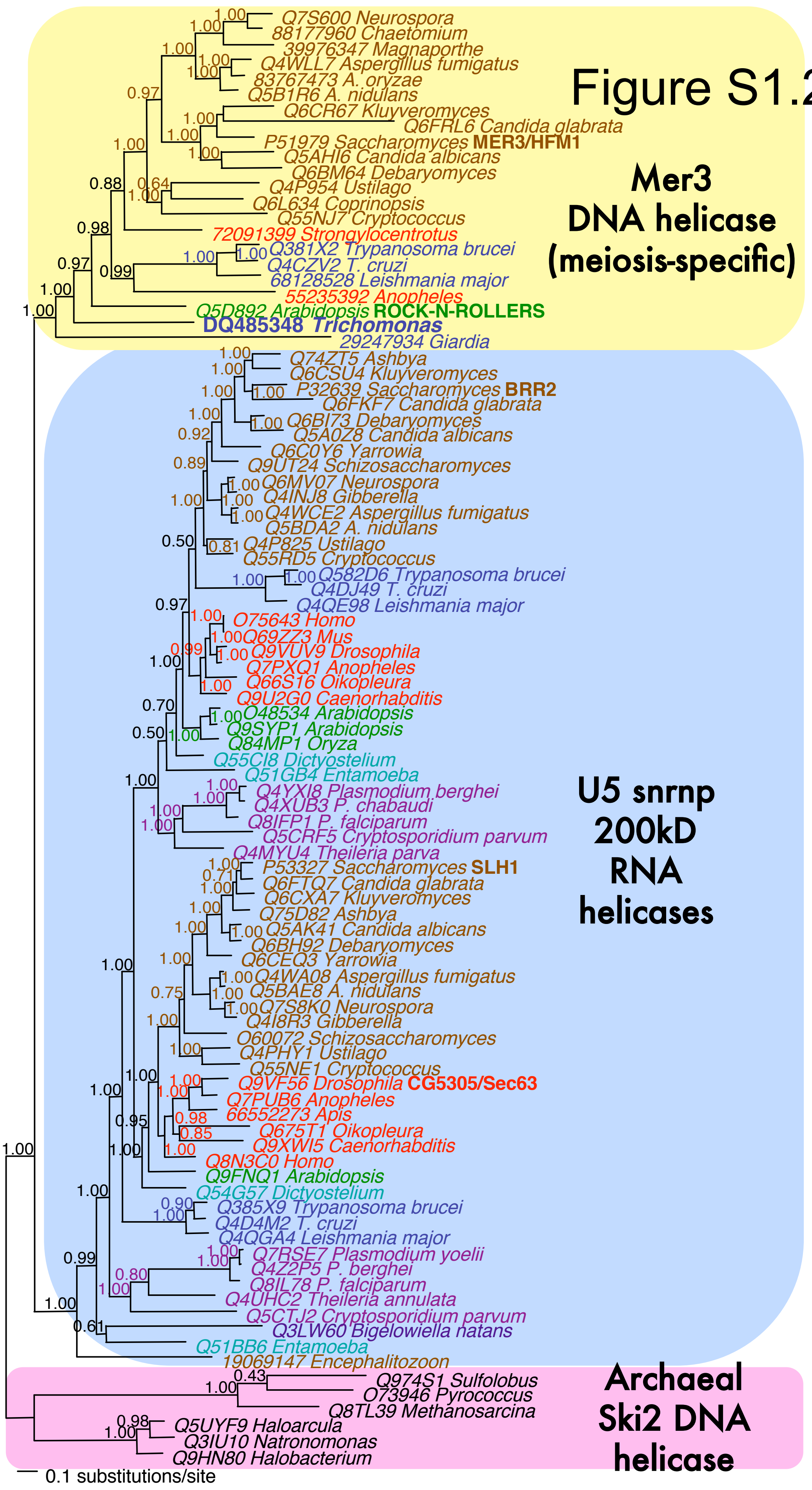

Q7S600 Neurospora
88177960 Chaetomium
39976347 Magnaporthe
Q4WLL7 Aspergillus fumigatus
83767473 A. oryzae
Q5B1R6 A. nidulans
Q6CR67 Kluyveromyces
Q6FRL6 Candida glabrata
P51979 Saccharomyces MER3/HFM1
Q5AHI6 Candida albicans
Q6BM64 Debaryomyces
Q4P954 Ustilago
Q6L634 Coprinopsis
Q55NJ7 Cryptococcus
72091399 Strongylocentrotus
Q381X2 Trypanosoma brucei
Q4CZV2 T. cruzi
68128528 Leishmania major
55235392 Anopheles
Q5D892 Arabidopsis ROCK-N-ROLLERS
DQ485348 Trichomonas
29247934 Giardia
Mer3
DNA helicase
(meiosis-specific)
Q74ZT5 Ashbya
Q6CSU4 Kluyveromyces
P32639 Saccharomyces BRR2
Q6FKF7 Candida glabrata
Q6BI73 Debaryomyces
Q5A0Z8 Candida albicans
Q6C0Y6 Yarrowia
Q9UT24 Schizosaccharomyces
Q6MV07 Neurospora
Q4INJ8 Gibberella
Q4WCE2 Aspergillus fumigatus
Q5BDA2 A. nidulans
Q4P825 Ustilago
Q55RD5 Cryptococcus
Q582D6 Trypanosoma brucei
Q4DJ49 T. cruzi
Q4QE98 Leishmania major
O75643 Homo
Q69ZZ3 Mus
Q9VUV9 Drosophila
Q7PXQ1 Anopheles
Q66S16 Oikopleura
Q9U2G0 Caenorhabditis
O48534 Arabidopsis
Q9SYP1 Arabidopsis
Q84MP1 Oryza
Q55CI8 Dictyostelium
Q51GB4 Entamoeba
Q4YXI8 Plasmodium berghei
Q4XUB3 P. chabaudi
Q8IFP1 P. falciparum
Q5CRF5 Cryptosporidium parvum
Q4MYU4 Theileria parva
P53327 Saccharomyces SLH1
Q6FTQ7 Candida glabrata
Q6CXA7 Kluyveromyces
Q75D82 Ashbya
Q5AK41 Candida albicans
Q6BH92 Debaryomyces
Q6CEQ3 Yarrowia
Q4WA08 Aspergillus fumigatus
Q5BAE8 A. nidulans
Q7S8K0 Neurospora
Q4I8R3 Gibberella
O60072 Schizosaccharomyces
Q4PHY1 Ustilago
Q55NE1 Cryptococcus
Q9VF56 Drosophila CG5305/Sec63
Q7PUB6 Anopheles
66552273 Apis
Q675T1 Oikopleura
Q9XWI5 Caenorhabditis
Q8N3C0 Homo
Q9FNQ1 Arabidopsis
Q54G57 Dictyostelium
Q385X9 Trypanosoma brucei
Q4D4M2 T. cruzi
Q4QGA4 Leishmania major
Q7RSE7 Plasmodium yoelii
Q4Z2P5 P. berghei
Q8IL78 P. falciparum
Q4UHC2 Theileria annulata
Q5CTJ2 Cryptosporidium parvum
Q3LW60 Bigelowiella natans
Q51BB6 Entamoeba
19069147 Encephalitozoon
U5 snrnp
200kD
RNA
helicases
Q974S1 Sulfolobus
O73946 Pyrococcus
Q8TL39 Methanosarcina
Q5UYF9 Haloarcula
Q3IU10 Natronomonas
Q9HN80 Halobacterium
Archaeal
Ski2 DNA
helicase
0.1 substitutions/site

Figure S1.28

Figure S1.29: Eukaryotic SMC homologs Smc1, Smc2, Smc3, Smc4, Smc5 and Smc6, outgroup-rooted with archaeal SMC. 378 aligned amino acid sites were analyzed, this consensus topology derived from 550 trees, $\alpha = 1.39$ ($1.28 < \alpha < 1.51$), pI = 0.02 (0.0007 < pI < 0.07) and lnL = –104724.55. Thickened lines correspond to well-supported nodes with posterior probabilities of 0.95 to 1.00.

Figure S1.29

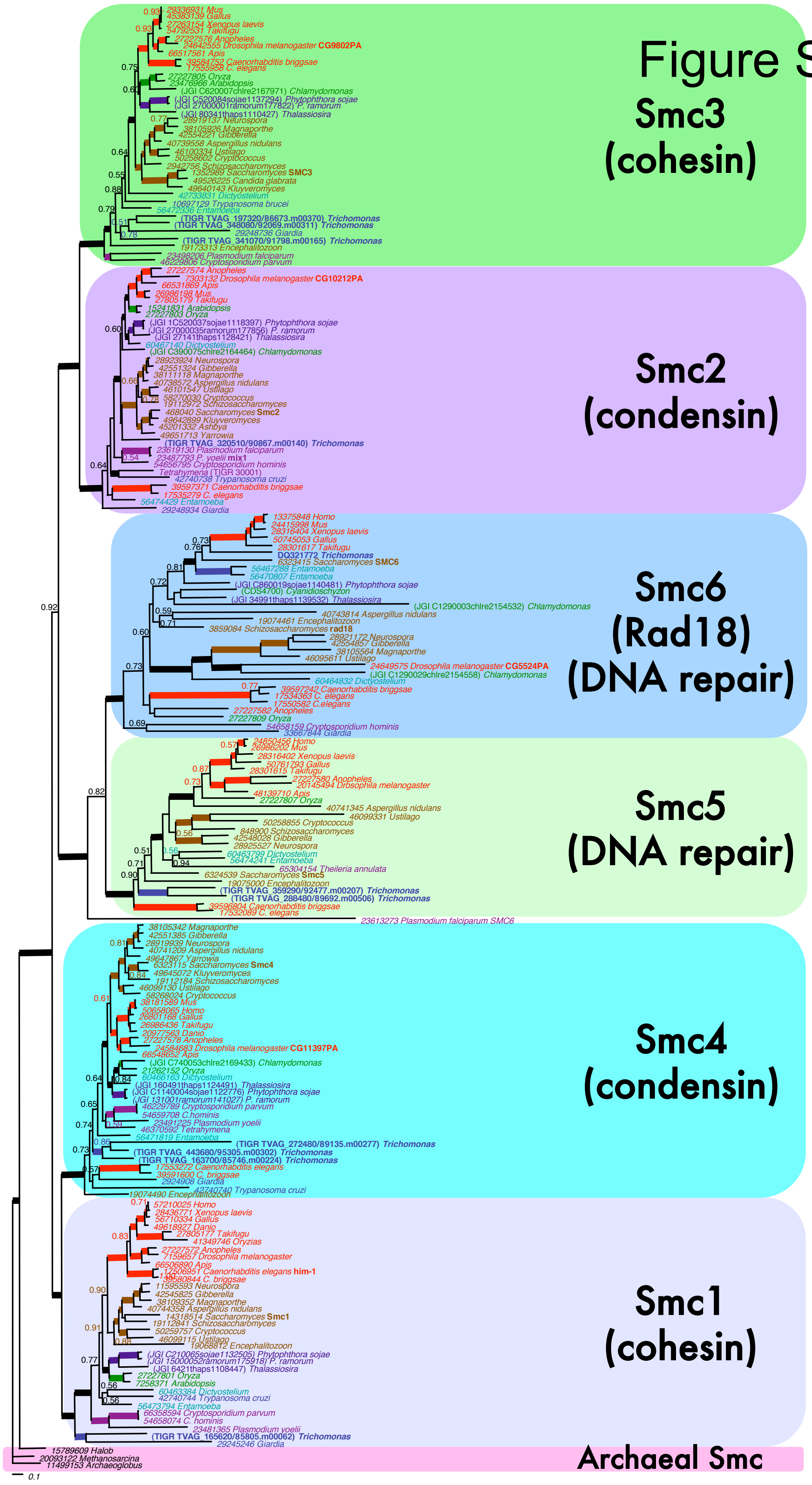

Smc3
(cohesin)
Smc2
(condensin)
Smc6
(Rad18)
(DNA repair)
Smc5
(DNA repair)
Smc4
(condensin)
Smc1
(cohesin)
Archaeal Smc
0.1

Figure S1.30: Rec8 homologs rooted with the paralog outgroup Rad21, with the most rapidly evolving sequences removed. 141 aligned amino acid sites were analyzed, this consensus topology derived from 650 trees, $\alpha = 1.60$ ($1.16 < \alpha < 2.16$), pI = 0.08 (0.005 < pI < 0.19) and lnL = –14945.15.

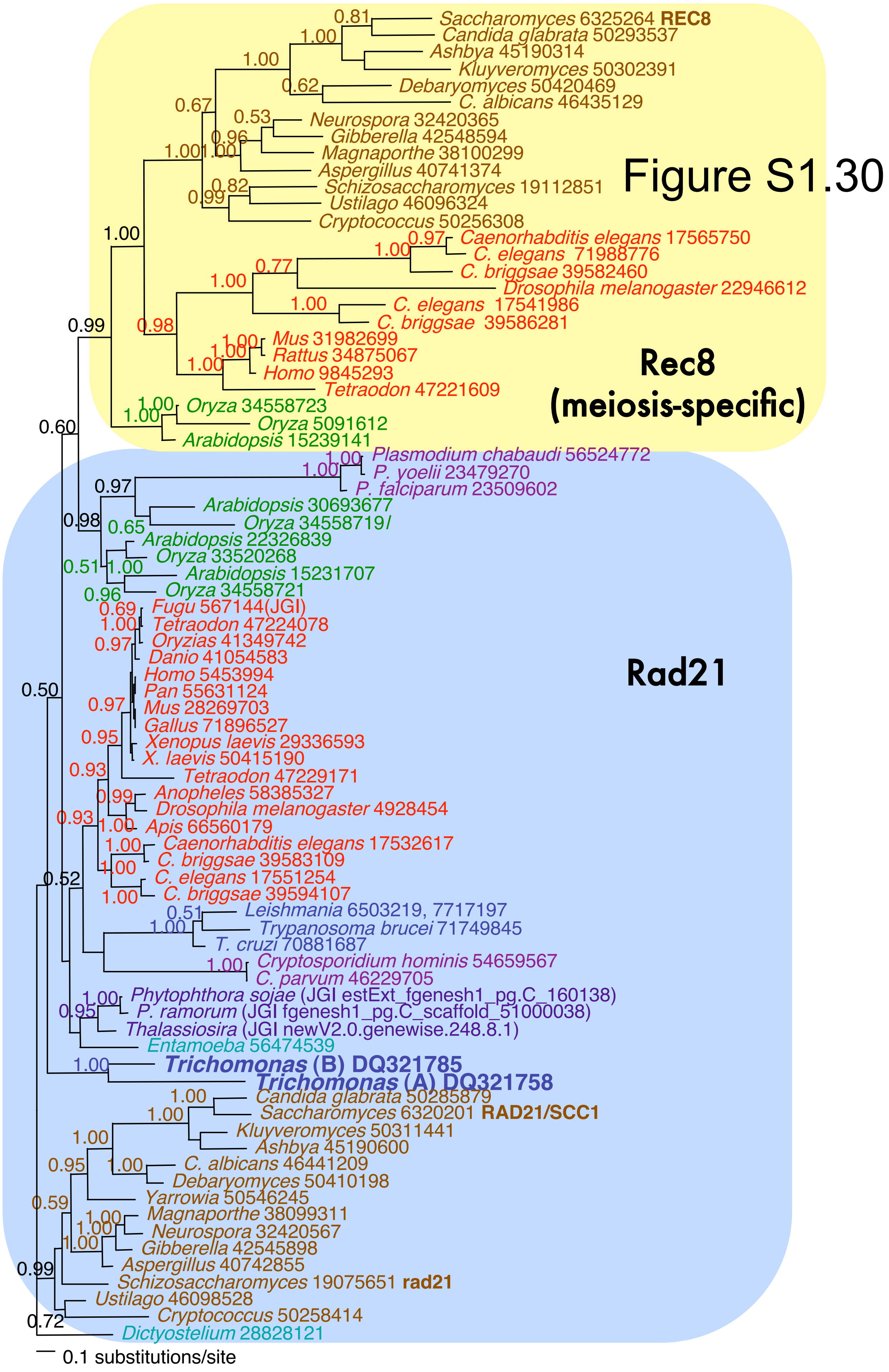
Figure S1.30
Rec8
(meiosis-specific)
Rad21
Saccharomyces 6325264 REC8
Candida glabrata 50293537
Ashbya 45190314
Kluyveromyces 50302391
Debaryomyces 50420469
C. albicans 46435129
Neurospora 32420365
Gibberella 42548594
Magnaporthe 38100299
Aspergillus 40741374
Schizosaccharomyces 19112851
Ustilago 46096324
Cryptococcus 50256308
Caenorhabditis elegans 17565750
C. elegans 71988776
C. briggsae 39582460
Drosophila melanogaster 22946612
C. elegans 17541986
C. briggsae 39586281
Mus 31982699
Rattus 34875067
Homo 9845293
Tetraodon 47221609
Oryza 34558723
Oryza 5091612
Arabidopsis 15239141
Plasmodium chabaudi 56524772
P. yoelii 23479270
P. falciparum 23509602
Arabidopsis 30693677
Oryza 34558719/
Arabidopsis 22326839
Oryza 33520268
Arabidopsis 15231707
Oryza 34558721
Fugu 567144(JGI)
Tetraodon 47224078
Oryzias 41349742
Danio 41054583
Homo 5453994
Pan 55631124
Mus 28269703
Gallus 71896527
Xenopus laevis 29336593
X. laevis 50415190
Tetraodon 47229171
Anopheles 58385327
Drosophila melanogaster 4928454
Apis 66560179
Caenorhabditis elegans 17532617
C. briggsae 39583109
C. elegans 17551254
C. briggsae 39594107
Leishmania 6503219, 7717197
Trypanosoma brucei 71749845
T. cruzi 70881687
Cryptosporidium hominis 54659567
C. parvum 46229705
Phytophthora sojae (JGI estExt_fgenesh1_pg.C_160138)
P. ramorum (JGI fgenesh1_pg.C_scaffold_51000038)
Thalassiosira (JGI newV2.0.genewise.248.8.1)
Entamoeba 56474539
Trichomonas (B) DQ321785
Trichomonas (A) DQ321758
Candida glabrata 50285879
Saccharomyces 6320201 RAD21/SCC1
Kluyveromyces 50311441
Ashbya 45190600
C. albicans 46441209
Debaryomyces 50410198
Yarrowia 50546245
Magnaporthe 38099311
Neurospora 32420567
Gibberella 42545898
Aspergillus 40742855
Schizosaccharomyces 19075651 rad21
Ustilago 46098528
Cryptococcus 50258414
Dictyostelium 28828121
0.1 substitutions/site

Figure S1.31: Rad21 homologs rooted with the paralog outgroup Rec8. 113 aligned amino acid sites were analyzed, this consensus topology derived from 800 trees, $\alpha = 2.02$ ($1.45 < \alpha < 2.70$), $pI = 0.06$ ($0.003 < pI < 0.17$) and $lnL = -12151.78$.

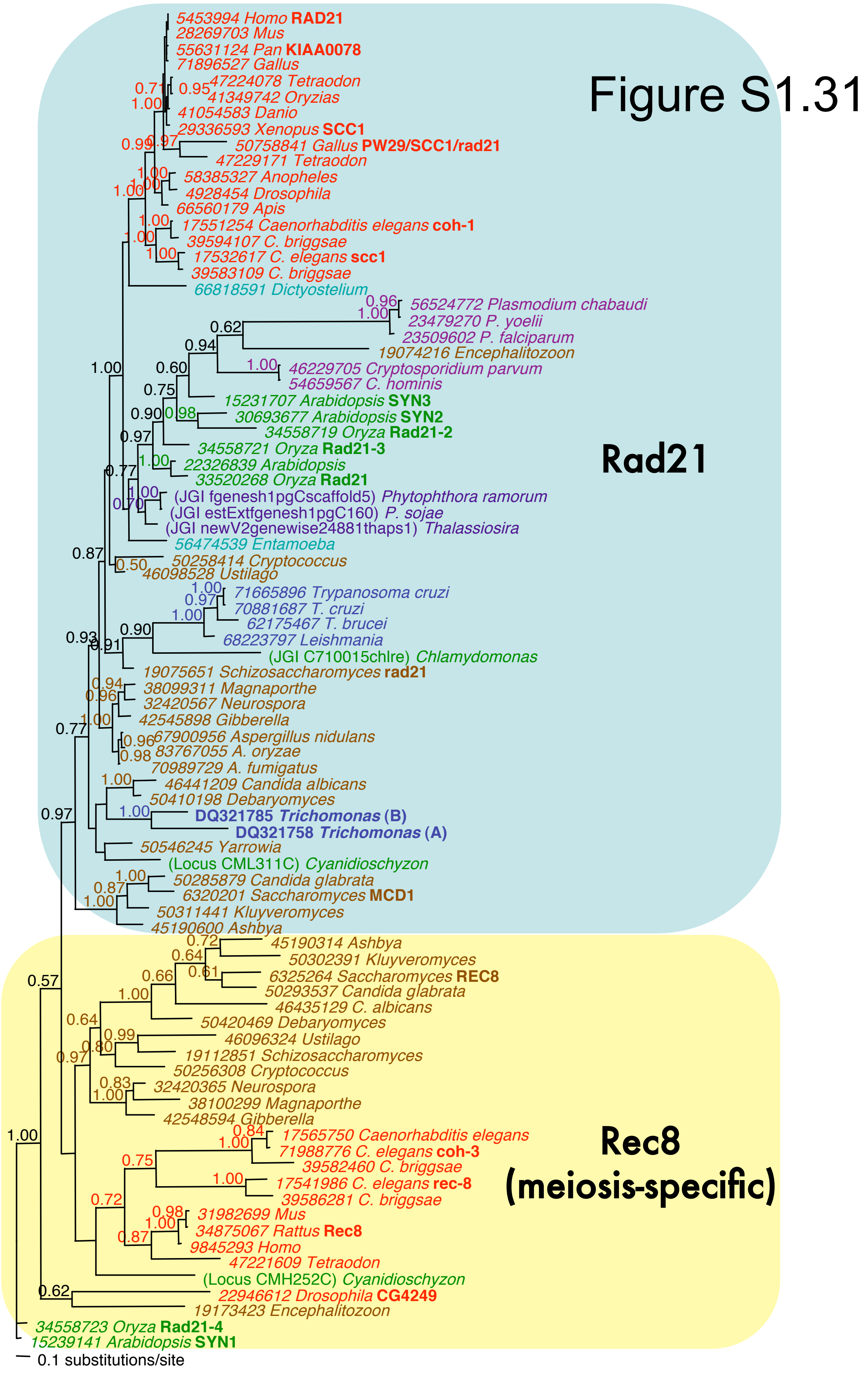
Figure S1.31
Rad21
Rec8
(meiosis-specific)
5453994 Homo RAD21
28269703 Mus
55631124 Pan KIAA0078
71896527 Gallus
47224078 Tetraodon
41349742 Oryzias
41054583 Danio
29336593 Xenopus SCC1
50758841 Gallus PW29/SCC1/rad21
47229171 Tetraodon
58385327 Anopheles
4928454 Drosophila
66560179 Apis
17551254 Caenorhabditis elegans coh-1
39594107 C. briggsae
17532617 C. elegans scc1
39583109 C. briggsae
66818591 Dictyostelium
56524772 Plasmodium chabaudi
23479270 P. yoelii
23509602 P. falciparum
19074216 Encephalitozoon
46229705 Cryptosporidium parvum
54659567 C. hominis
15231707 Arabidopsis SYN3
30693677 Arabidopsis SYN2
34558719 Oryza Rad21-2
34558721 Oryza Rad21-3
22326839 Arabidopsis
33520268 Oryza Rad21
(JGI fgenesh1pgCscaffold5) Phytophthora ramorum
(JGI estExtfgenesh1pgC160) P. sojae
(JGI newV2genewise24881thaps1) Thalassiosira
56474539 Entamoeba
50258414 Cryptococcus
46098528 Ustilago
71665896 Trypanosoma cruzi
70881687 T. cruzi
62175467 T. brucei
68223797 Leishmania
(JGI C710015chlre) Chlamydomonas
19075651 Schizosaccharomyces rad21
38099311 Magnaporthe
32420567 Neurospora
42545898 Gibberella
67900956 Aspergillus nidulans
83767055 A. oryzae
70989729 A. fumigatus
46441209 Candida albicans
50410198 Debaryomyces
DQ321785 Trichomonas (B)
DQ321758 Trichomonas (A)
50546245 Yarrowia
(Locus CML311C) Cyanidioschyzon
50285879 Candida glabrata
6320201 Saccharomyces MCD1
50311441 Kluyveromyces
45190600 Ashbya
45190314 Ashbya
50302391 Kluyveromyces
6325264 Saccharomyces REC8
50293537 Candida glabrata
46435129 C. albicans
50420469 Debaryomyces
46096324 Ustilago
19112851 Schizosaccharomyces
50256308 Cryptococcus
32420365 Neurospora
38100299 Magnaporthe
42548594 Gibberella
17565750 Caenorhabditis elegans
71988776 C. elegans coh-3
39582460 C. briggsae
17541986 C. elegans rec-8
39586281 C. briggsae
31982699 Mus
34875067 Rattus Rec8
9845293 Homo
47221609 Tetraodon
(Locus CMH252C) Cyanidioschyzon
22946612 Drosophila CG4249
19173423 Encephalitozoon
34558723 Oryza Rad21-4
15239141 Arabidopsis SYN1
0.1 substitutions/site

Figure S1.32: Pds5 homologs, unrooted. 770 aligned amino acid sites were analyzed, this consensus topology derived from 800 trees, $\alpha = 4.23$ ($3.72 < \alpha < 4.80$), pI = 0.006 (0.002 < pI < 0.013) and lnL = –53078.71.

Figure S1.32

Pds5

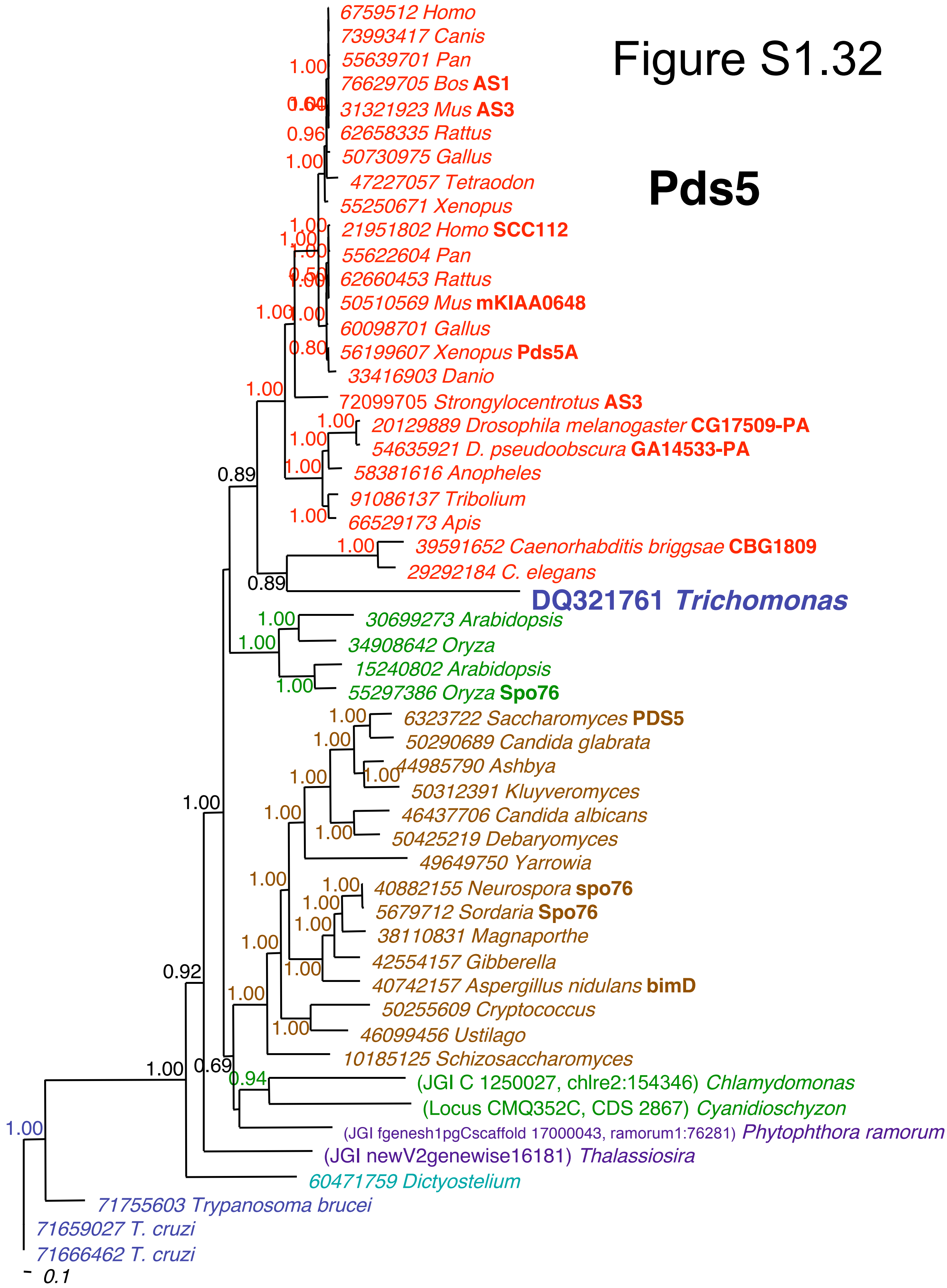

Figure S1.33: Scc3 homologs, unrooted. 308 aligned amino acid sites were analyzed, this consensus topology derived from 900 trees, $\alpha = 3.64$ ($2.96 < \alpha < 4.48$), pI = 0.03 (0.01 < pI < 0.06) and lnL = –22387.72.

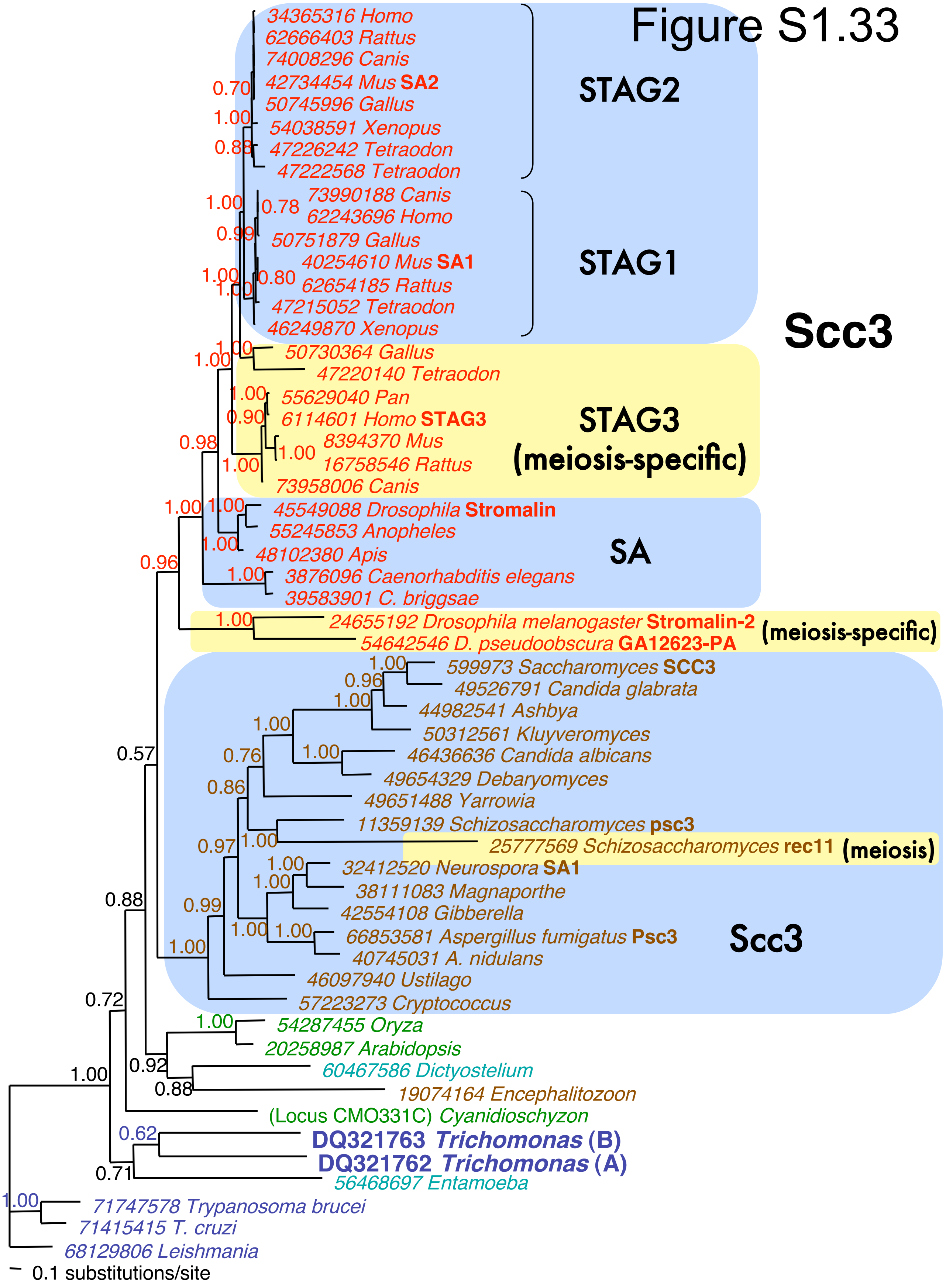

Figure S1.33
STAG2
STAG1
Scc3
STAG3
(meiosis-specific)
SA
(meiosis-specific)
(meiosis)
Scc3
34365316 Homo
62666403 Rattus
74008296 Canis
42734454 Mus SA2
50745996 Gallus
54038591 Xenopus
47226242 Tetraodon
47222568 Tetraodon
73990188 Canis
62243696 Homo
50751879 Gallus
40254610 Mus SA1
62654185 Rattus
47215052 Tetraodon
46249870 Xenopus
50730364 Gallus
47220140 Tetraodon
55629040 Pan
6114601 Homo STAG3
8394370 Mus
16758546 Rattus
73958006 Canis
45549088 Drosophila Stromalin
55245853 Anopheles
48102380 Apis
3876096 Caenorhabditis elegans
39583901 C. briggsae
24655192 Drosophila melanogaster Stromalin-2
54642546 D. pseudoobscura GA12623-PA
599973 Saccharomyces SCC3
49526791 Candida glabrata
44982541 Ashbya
50312561 Kluyveromyces
46436636 Candida albicans
49654329 Debaryomyces
49651488 Yarrowia
11359139 Schizosaccharomyces psc3
25777569 Schizosaccharomyces rec11
32412520 Neurospora SA1
38111083 Magnaporthe
42554108 Gibberella
66853581 Aspergillus fumigatus Psc3
40745031 A. nidulans
46097940 Ustilago
57223273 Cryptococcus
54287455 Oryza
20258987 Arabidopsis
60467586 Dictyostelium
19074164 Encephalitozoon
(Locus CMO331C) Cyanidioschyzon
DQ321763 Trichomonas (B)
DQ321762 Trichomonas (A)
56468697 Entamoeba
71747578 Trypanosoma brucei
71415415 T. cruzi
68129806 Leishmania
0.1 substitutions/site
